# Molecular signatures of neurodegeneration in the cortex of PS1/PS2 double knockout mice

**DOI:** 10.1186/1750-1326-3-14

**Published:** 2008-10-03

**Authors:** Károly Mirnics, Eric M Norstrom, Krassimira Garbett, Se Hoon Choi, Xiaoqiong Zhang, Philip Ebert, Sangram S Sisodia

**Affiliations:** 1 Department of Psychiatry, Vanderbilt University, Nashville, TN37232, USA; 2 Kennedy Center for Research on Human Development, Vanderbilt University, Nashville, TN37232, USA; 3 Department of Neurobiology, University of Chicago, Chicago, IL60637, USA

## Abstract

**Background:**

Familial Alzheimer's disease-linked variants of presenilin (PSEN1 and PSEN2) contribute to the pathophysiology of disease by both gain-of-function and loss-of-function mechanisms. Deletions of *PSEN1 *and *PSEN2 *in the mouse forebrain result in a strong and progressive neurodegenerative phenotype which is characterized by both anatomical and behavioral changes.

**Results:**

To better understand the molecular changes associated with these morphological and behavioral phenotypes, we performed a DNA microarray transcriptome profiling of the hippocampus and the frontal cortex of the *PSEN1/PSEN2 *double knock-out mice and littermate controls at five different ages ranging from 2–8 months. Our data suggest that combined deficiencies of *PSEN1 *and *PSEN2 *results in a progressive, age-dependent transcriptome signature related to neurodegeneration and neuroinflammation. While these events may progress differently in the hippocampus and frontal cortex, the most critical expression signatures are common across the two brain regions, and involve a strong upregulation of *cathepsin *and *complement *system transcripts.

**Conclusion:**

The observed neuroinflammatory expression changes are likely to be causally linked to the neurodegenerative phenotype observed in mice with compound deletions of *PSEN1 *and *PSEN2*. Furthermore, our results suggest that the evaluation of inhibitors of PS/γ-secretase activity for treatment of Alzheimer's Disease must include close monitoring for signs of calpain-cathepsin system activation.

## Background

Presenilins (PS) are highly homologous polytopic membrane proteins that play a critical role in intramembranous processing of amyloid precursor proteins (APP), leading to the production of Aβ peptides (for reviews, see [1-4]). Inheritance of mutations in *PSEN1 *and *PSEN2*, that encode PS1 and PS2, respectively, cause familial forms of Alzheimer's disease (FAD) [5,6], and do so by elevating the ratio of Aβß_42_/Aβ_40 _peptides [7-10]. Hence, it has been argued that FAD-linked PS1 variants cause disease primarily through a gain of function mechanism. However, recent studies have shown that these PS variants also result in a significant loss of *γ*-secretase function that may critically contribute to the pathophysiology of FAD (for a review, see [11]).

Over the last several years we assessed the gene expression profiles of *PSEN1 *knockout mice and *PSEN1 *mutant transgenic animals, and have defined a set of *PSEN1*-dependent genes [12-15]. However, it is important to point out that due to redundant functions and distributions of PSEN1 and PSEN2 [16,17], cortical *PSEN1 *ablation in mice results in only a limited phenotype. In contrast, forebrain *PSEN1/PSEN2 *double KO mice show a strong and progressive neurodegenerative phenotype which is characterized by both anatomical and behavioral changes [11,18]. Although anatomical changes are not apparent at 2 months of age, these mice already show mild memory impairments at this age. The behavioral phenotype progresses with time and the mutant mice begin to exhibit excessive grooming behavior, increased stereotypy in the open field, and reduced latency in the rotarod test at the age of 6 months [18]. The anatomical changes develop over time, and the progressive thinning of cortical layers becomes prominent by the age of 6 months.

To gain a better understanding of presenilin-dependent neurodegeneration in the cortex, we performed an expression profiling of the hippocampus (HC) and frontal cortex (FC) of mice with forebrain ablations of *PSEN1 *and *PSEN2*. We used DNA microarrays to analyze the transcriptome at five developmental time points. We chose to examine the temporal nature of gene expression changes because the *PSEN1 *allele is conditionally deleted by a CamKII-cre allele that promotes recombination of "floxed" loci first in the hippocampus, and then spreads throughout the entire forebrain. We focused our attention on the following questions: 1) what are the gene expression consequences of *PSEN1/PSEN2 *ablation; 2) do the altered genes share common functional characteristics; 3) how does gene expression change over time as a function of *PSEN1/PSEN2 *deletion; 4) are the molecular signatures of neurodegeneration similar in the HC and FC and 5) is presenilin-dependent molecular neurodegeneration progressing similarly in FC and HC?

## Results

**Figure 1:**
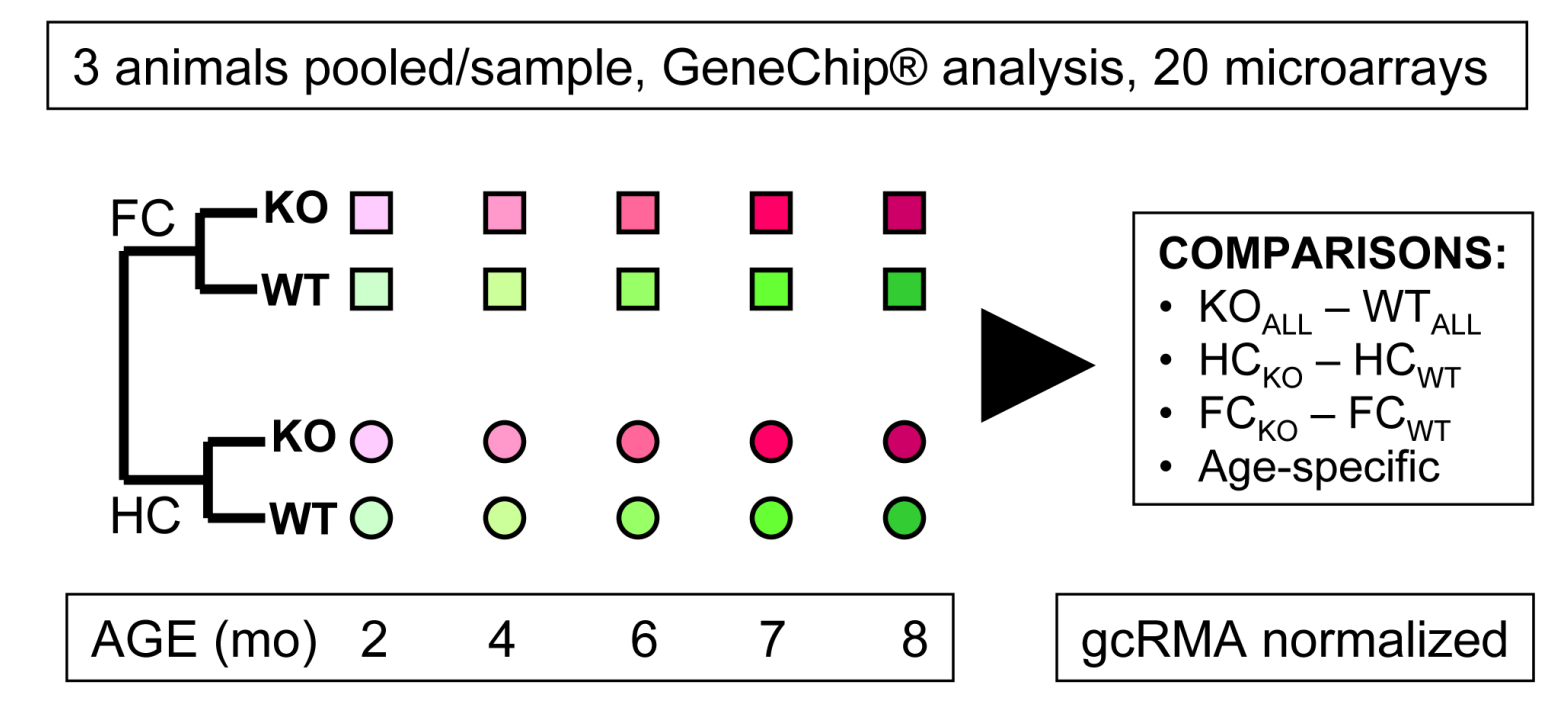
**Experimental Design**. The frontal cortical (FC) and hippocampal (HC) transcriptome of PS1/PS2 double-knockout animals (KO) was compared to that of wild-type controls (WT) using Affymetrix oligonucleotide microarrays. The samples from 5 different ages for each microarray were prepared from a pool of 3 animals. Data were GCOS segmented, gcRMA normalized and compared using several experimental paradigms, all employing pairwise comparison statistics.

The experimental design and analysis strategy are outlined in Figure 1. Briefly, frontal and hippocampal cortices of PSEN1/PSEN2 KO (PSKO) and matched control animals of different postnatal ages were analyzed by MG_430 oligonucleotide arrays by Affymetrix. An RMA-based normalization was followed by a multi-level analysis that compared the samples based on the genotype, age and brain region.

### Gene expression differences regardless of brain region or age

In a genotype-driven analysis we observed a strong transcriptome signature between the PSKO and CNT mice. 113 DNA microarray probesets showed significant gene expression differences (|ALR| > 0.585, p < 0.05). Of these, 41 probes reported underexpression, while 72 showed upregulation in the PSKO animals, suggesting that the removal of the PS genes is characterized by a complex set of transcriptional events (Additional File 1). Two-way hierarchical clustering of the differentially expressed probe sets revealed a separation of samples according to genotype (Additional File 2). As the altered gene expression products performed a wide variety of cellular functions, we subjected this dataset to a pathway analysis based on predefined cellular processes (BioCarta). This analysis revealed that the PSKO animals showed a statistically significant populational downregulation of transcripts related to the GABA pathway and GPCR function, while 12 pathways showed upregulation in the PSKO sample (Table 1). Notably, the increased transcript networks strongly suggest and ongoing inflammation and/or neurodegeneration in the experimental animals (e.g. CALPAIN, TOB1, TGFβ and other related pathways – see Additional File 3 for complete analysis results). This initial analysis was followed by a region*genotype assessment.

**Table 1:** Differentially expressed gene groups between the PSKO and CNT mice regardless of age or brain region.

#	**NAME**	**SIZE**	**ES**	**NES**	***p-val***	**q-val**
1	GABAPATHWAY	11	0.68	1.59	***0.046748***	0.147418

2	AGPCRPATHWAY	10	0.67	1.45	***0.019068***	0.243600

3	RAC1PATHWAY	21	-0.59	-1.30	***0.020121***	0.418927

4	D4GDIPATHWAY	10	-0.68	-1.31	***0.043478***	0.397110

5	ALKPATHWAY	32	-0.55	-1.34	***0.014085***	0.298667

6	NTHIPATHWAY	19	-0.61	-1.35	***0.040936***	0.290866

7	TGFBPATHWAY	13	-0.67	-1.42	***0.010142***	0.163844

8	MCALPAINPATHWAY	22	-0.53	-1.44	***0.005848***	0.179323

9	INTRINSICPATHWAY	20	-0.58	-1.45	***0.020534***	0.177983

10	LAIRPATHWAY	10	-0.67	-1.45	***0.046185***	0.198237

11	VITCBPATHWAY	10	-0.51	-1.45	***0.038776***	0.228195

12	TOB1PATHWAY	14	-0.73	-1.63	***0.004082***	0.087297

13	EXTRINSICPATHWAY	12	-0.71	-1.69	***0.014614***	0.082402

14	NKCELLSPATHWAY	15	-0.50	-1.70	***0.025490***	0.138997

The whole dataset was subjected to pathway analysis with BioCarta using GSEA. Significantly enriched pathways are presented in either direction (positive NES – enriched in CNT; negative NES – enriched in PSKO). *Abbreviations: *SIZE- number of non-redundant probes in a pathway; ES – enrichment score; NES – normalized enrichment score; p-val – groupwise p value; q-val – groupwise false discovery ratio estimate. **Note **that only 2 pathways were enriched in the CNT mice (GABA and AGPCR), while the PSKO mice samples were enriched in 12 pathways.

### Gene expression differences in the frontal cortex (FC)

**Figure 2:**
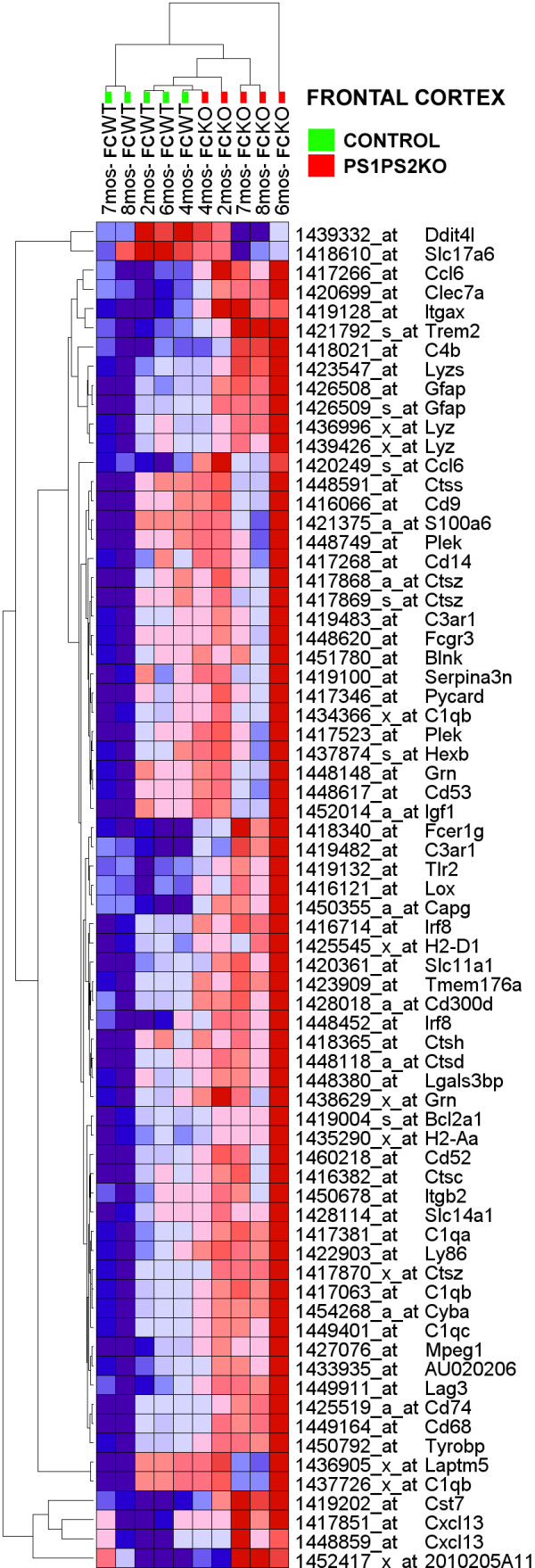
**Hierarchical clustering of genes that show differential expression in the FC of PSKO and CNT mice**. The numerical data are presented in Additional File 4. Gene probes are clustered in rows (denoted by symbols and probe identifiers), microarrays are clustered in columns (denoted by sample identifiers). Each pixel represents a single, color-coded gene expression value form FC form a PSKO or CNT mouse sample at five developmental ages. Shades of red correspond to the magnitude of expression increase, the intensity of blue corresponds to the magnitude of the reduction in gene transcripts. Note that the frontal cortical PSKO and CNT samples, regardless of developmental age, cluster on different ends of the dendrogram.

In the region*genotype assessment the FC of PSKO and CNT mice reported 73 differentially expressed probesets (|ALR| > 0.585, p < 0.05; Additional File 4). Importantly, the distribution of these expression changes was highly skewed. Of the 73 probesets 70 reported an upregulation (96%), while only 2 probesets were downregulated (4%). The clustering of these data also separated the FC samples according to their proper genotype (Figure 2).

### Gene expression differences in the hippocampus (HC)

**Figure 3:**
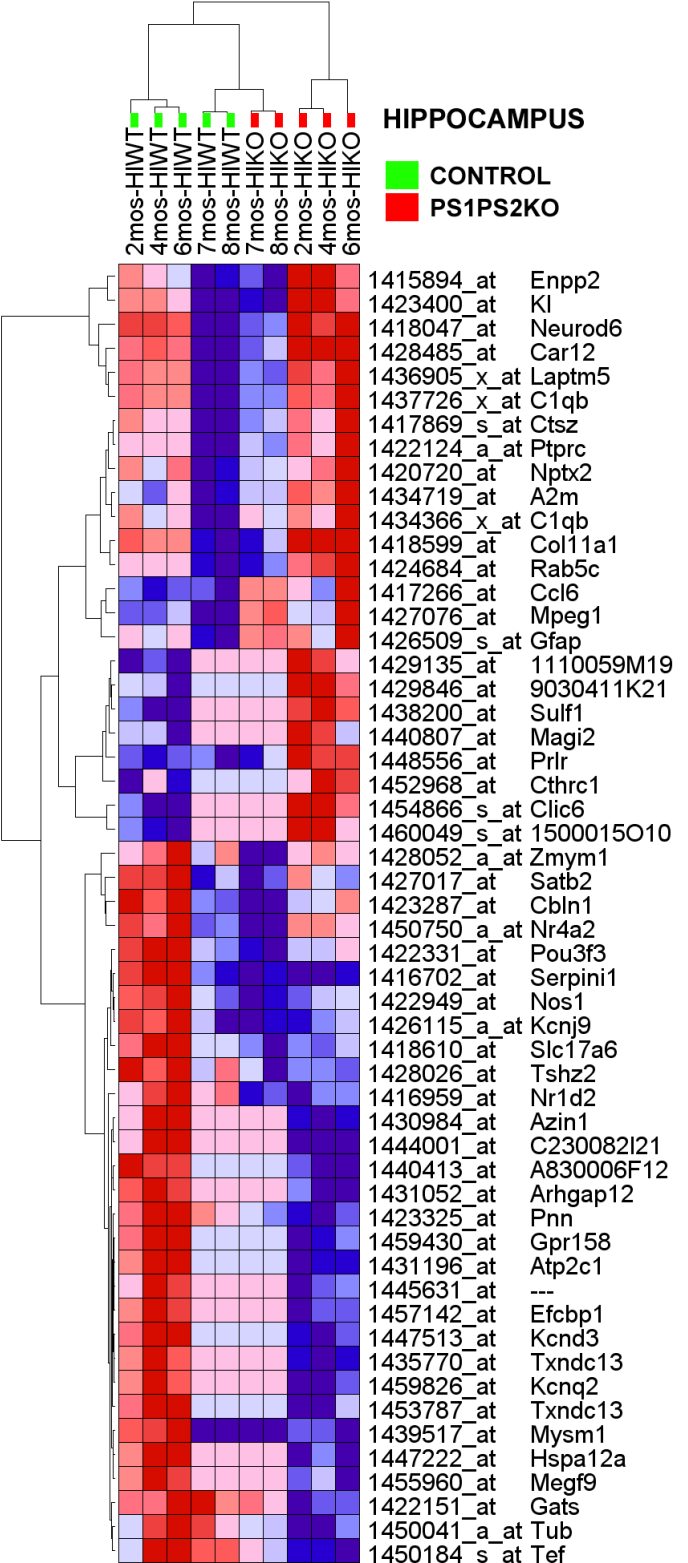
**Hierarchical clustering of genes that show differential expression in the HC of PSKO and CNT mice**. The numerical data are presented in Additional File 5. Gene probes are clustered in rows (denoted by symbols and probe identifiers), while microarrays are clustered in columns (denoted by sample identifiers). Figure layout similar to that in *Figure 2*. Note that the frontal cortical PSKO and CNT samples, regardless of developmental age, cluster on different ends of the dendrogram.

In the region*genotype assessment of the HC of PSKO and CNT mice, we did not observe a dominant downregulation of transcripts. Rather, the HC of PSKO and CNT mice reported an opposite pattern of that seen in the FC: of the 137 probesets reporting an expression difference in HC only 37 (26%) reported an upregulation while 101 (74%) probesets showed an underexpression in the experimental animals (Additional File 5). Notably, the distribution of the HC expression changes was also highly skewed, but in the opposite direction from that observed in the FC. Finally, the clustering of these data in the vertical dimension also separated the HC of the PSKO samples from those of matched controls (Figure 3).

### Gene expression similarities and differences in the FC and HC of PSKO mice

**Figure 4:**
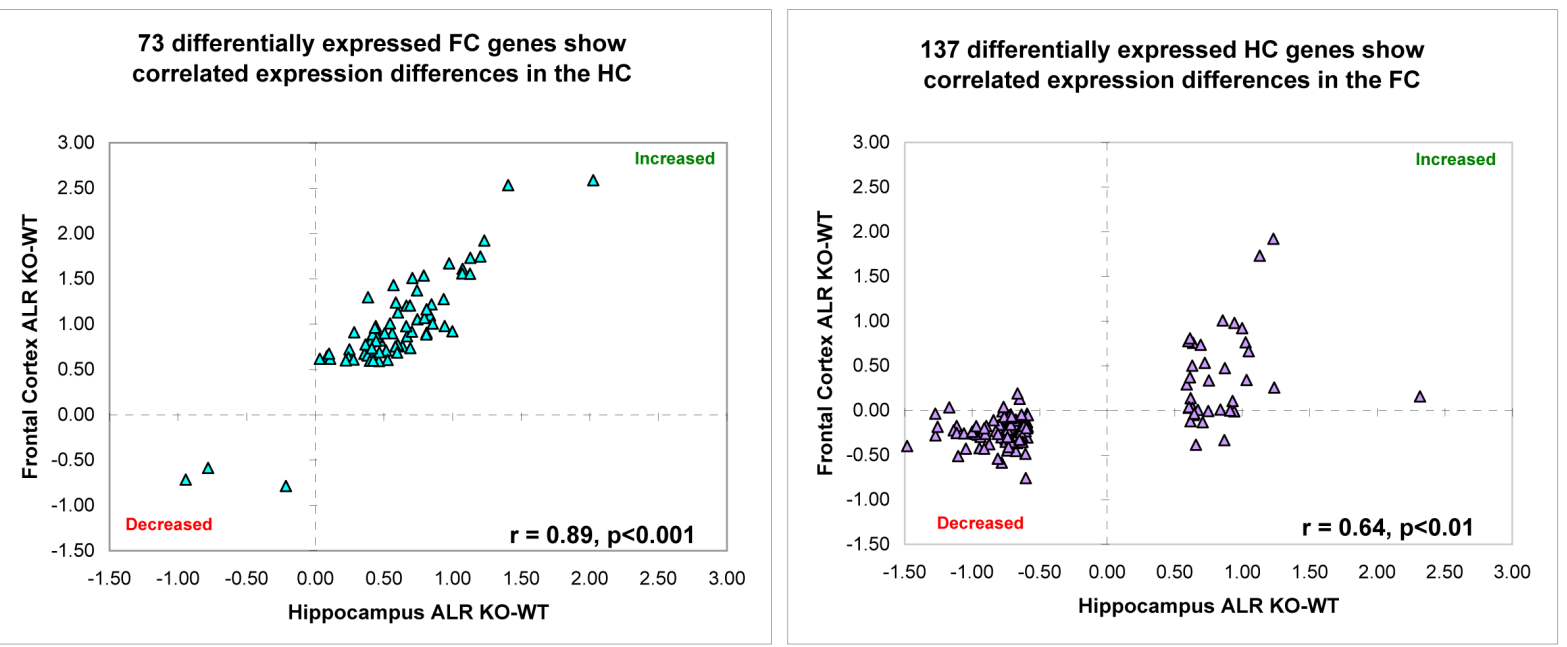
**HC and FC expression changes are highly correlated**. Both plots denote the ALR_PSKO-CNT _in the HC (X axis) and FC (Y axis). Each blue diamond represents a single, differentially expressed gene probe. Red dashed line denotes linear regression of the dataset. A. PSKO FC samples reported 70 genes differentially expressed form the FC of CNTs. These genes also showed a highly correlated expression pattern in the HC comparison (Pearson r = 0.89, p < 0.001) B. PSKO HC samples reported 53 genes differentially expressed form the HC of CNTs. These genes also showed a highly correlated expression pattern in the FC comparison (Pearson r = 0.67, p < 0.005).

Although the most significant changes were different in the FC and HC, the overall gene expression patters were very much alike across the two brain regions. The magnitude of change between the PSKO and WT mice (ALR) of the 73 genes probesets that were found differentially expressed in the FC were very highly correlated with the gene expression changes observed in the HC (r = 0.89, p < 0.001) (Figure 4A). Similarly, the 137 gene probesets that showed differential expression in the HC of PSKO and WT mice also showed a correlation with the expression changes for same genes in the FC (Figure 4B). This correlation of the *HC to FC *correlation was somewhat weaker (r = 0.64, p < 0.01) than that observed for the *FC to HC *differentially expressed probesets.

**Figure 5:**
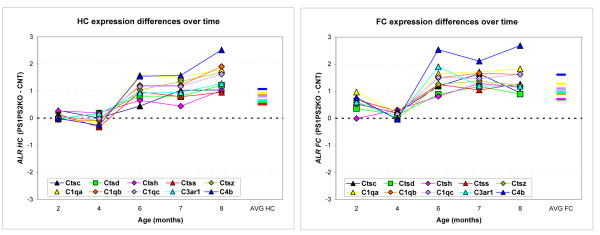
**PS ablation-dependent expression changes in *cathepsin *and *complement *transcripts as a function of postnatal age**. The expression changes of 5 cathepsin (*Ctsc*, *Ctsd*, *Ctsh*, *Ctss *and *Ctsz*) and 5 complement system transcripts (*C1qa*, *C1qb*, *C1qc*, *C3ar1 *and *C4b*) were examined in more detail. In both graphs X axis denotes age of mice, while Y axis denotes pairwise expression difference between the PSKO mice and matched WT samples. Each color line represents change in expression over time for a single gene. A) FC. B) HC. Note the similarity of the age-dependent transcript increase in the PSKO animals across the two brain regions.

**Figure 6:**
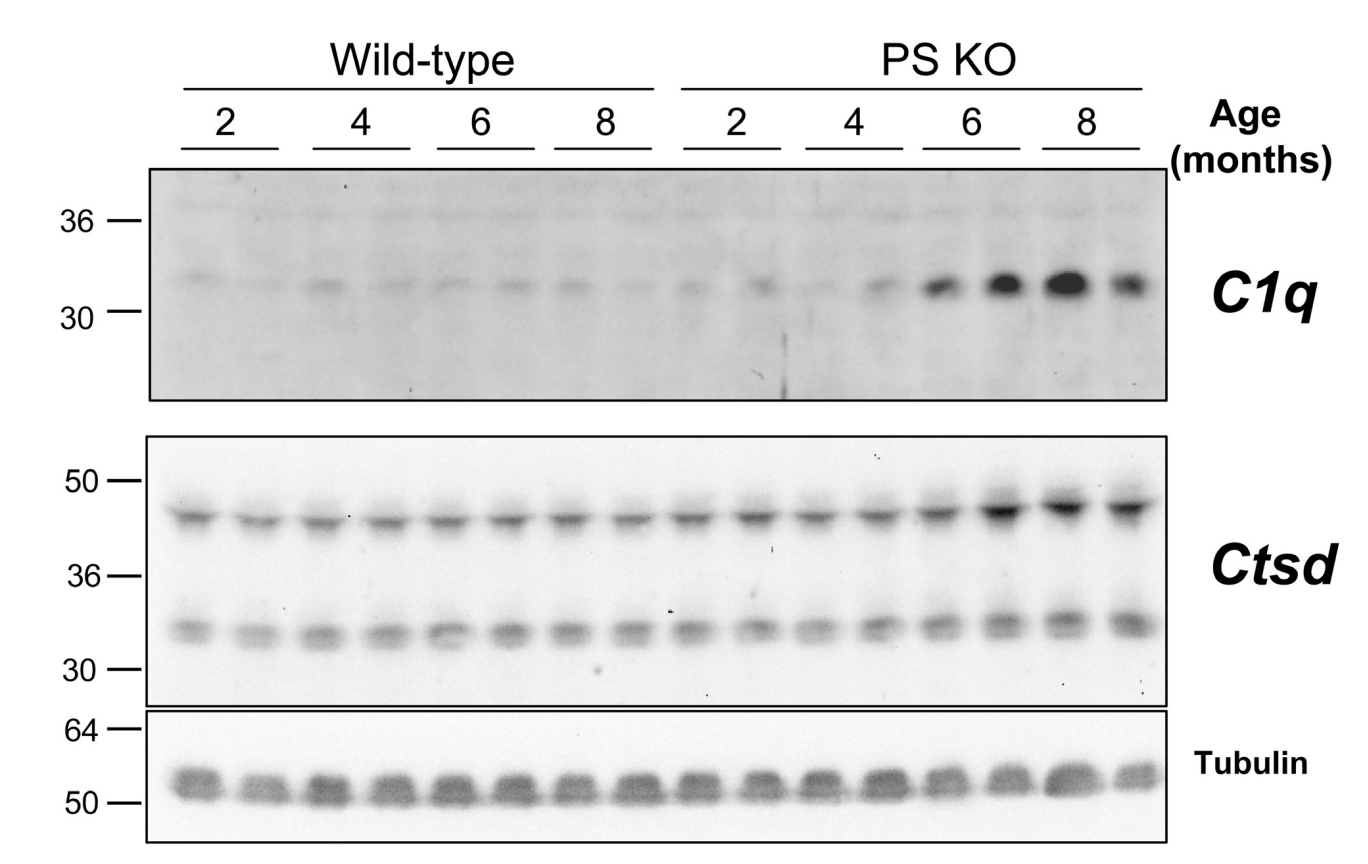
**Age-dependent upregulation of *C1q *and *Ctsd *in PS knockout mice**. The same nitrocellulose membrane was re-probed for *Ctsd*, *C1q*, and Tubulin. Note a progressive, age-dependent upregulation of *C1q *and *Ctsd *in the KO mice but not the matched wild-type controls. Tubulin was used as a loading control. Molecular weights of *Ctsd *species correlate best to active intermediate (48 kD) and mature heavy chain (34 kD).

Finally, we observed two important gene families that were differentially expressed both in the FC and the HC (Figure 5). Five members of the *cathepsin *and five members of the *complement *gene families showed a progressive, age-dependent overexpression in the PSKO mice. The progression of this overexpression was similar in time course and magnitude across the FC and HC. Furthermore, in addition to the increased transcript levels, two investigated genes, Ctsd and C1q showed the predicted, progressive induction in protein expression on a Western blot in a new set of experimental and control animals (Figure 6).

To determine if this was a true upregulation in the PSKO samples or it represented a failure to downregulate expression levels as part of normal development, we examined the normal developmental expression levels of these genes in the CNT mice (Additional File 6). As the expression levels of all these genes were stable in the tested age range (2–8 months) in both the FC and HC, we can conclude that the observed overexpression is indeed a result of an active transcriptional upregulation in the PSKO brain samples.

## Discussion

Over the past few years, we have used gene microarray strategies to examine transcript levels in brains of mice with conditionally inactivated *PSEN1 *(*cPS1 KO*) alleles. While these studies have proven informative, redundant function and distribution of *PSEN1 *and *PSEN2 *in hippocampus and cortex [16,17] has limited the interpretation of our findings. Indeed, while *cPS1KO *mice fail to exhibit any neuroanatomical or behavioral alterations [19,20], *cPS1KO *mice on a *PSEN2 KO *background show a strong and progressive neurodegenerative phenotype that is characterized by both anatomical and behavioral changes [11,18]. These animals do not exhibit anatomical changes at 2 months of age, yet show mild memory impairments at this age. The behavioral phenotypes progress over time and are paralleled by considerable cortical atrophy and neurodegeneration. In the present study, we performed expression profiling in order to develop an understanding of the mechanism(s) that might mediate these morphological and behavioral observations, and we now offer several novel insights. First, we show that simultaneous ablation of *PSEN1 *and *PSEN2 *leads to a wide variety of gene expression changes that progress over time. Second, the frontal cortex and hippocampus are both affected in PSKO brains, albeit by the notable difference that gene expression changes in the FC are characterized by elevated transcript levels, while the HC is mostly characterized by reduced transcript levels. Third, the transcriptome changes suggest a progressive neurodegenerative process with strong immune system activation. Fourth, based on the BioCarta pathway analysis, it appears that GABA-ergic neurons may be a preferential early target of the neurodegenerative events. Fifth, the *Complement *and *Cathepsin *systems appear to be critical contributors to neurodegeneration in both the hippocampus and frontal cortex.

One of the most intriguing findings of the manuscript is that transcript repressions dominate in the FC, while transcript inductions are the hallmark of the HC tissue. Although the most prominent presenilin deletion induced transcript changes may be different between these two brain regions, the deletion signature of the FC expression change was strong in HC and vice versa. We interpret these findings that the neurodegenerative events in FC lag behind of those in HC, suggesting that the early stages of degeneration are characterized by a strong, widespread repression of mRNA species. However, as the pathophysiological process proceeds, in addition to the transcript repression, a *de novo *inflammation-related transcript induction occurs, which becomes a dominant feature of a gene expression profile in the HC. Alternatively, the differential cytoarchitecture and molecular composition of FC and HC provide a differential framework for the adaptational responses of these two brain regions, and results in a different gene expression profile.

Presenilins associate with Aph1, Pen2 and Nicastrin in a large complex that mediates intramembranous, "*γ*-secretase" [1] proteolysis of a variety of type-I membrane proteins, including Notch, APP, E-cadherins, N-cadherins, and p75NTR, amongst others (reviewed by [21]). For most of these proteins, *γ*-secretase-mediated processing results in the generation of intracellular derivatives, termed ICDs, that have been shown to initiate complex signaling cascades, including those involved in cell adhesion, lateral inhibition of cell fate decisions, neurotrophin signaling and cell differentiation [22,23]. It has also become increasingly evident that PS have functions that go beyond their role as the catalytic entity of *γ*-secretase activity. For example, PS located in the endoplasmic reticulum (ER) and Golgi apparatus regulate calcium homeostasis [24], findings that may be equally relevant for neurodegeneration observed in the PSKO mice. Not surprisingly, with increasing age, the PSKO mice develop striking neurodegeneration of the cerebral cortex and worsening impairments of memory and synaptic function [18]. However, it is important to point out that gene expression changes arise before noticeable anatomical neurodegeneration. The progressive gene expression changes that we observed in both hippocampus and frontal cortex also appear to suggest that the cortical GABA-ergic interneurons may be one of the early targets of neurodegeneration mediated by the ablation of *PSEN *genes. As the GABA-ergic system has a strong influence on cognitive behaviors [25,26], the expression changes in interneurons are likely to be related to the altered cognitive processes reported in these mice. On the other hand, interneurons have been shown to exhibit enhanced vulnerability to various insults [27]. These events likely result in necrosis-induced microglial activation, accompanied by overexpression of the complement system transcripts, leading to neuroinfammatory processes [28], thus further accelerating the neurodegenerative processes.

The mechanism(s) by which ablation of *PSEN *genes leads to neurodegeneration are not fully understood, but likely involve several converging pathways. In this regard, our dataset is highly informative: first, we observe a strong and progressive increase in expression of transcripts encoding molecules in the calpain-cathepsin system. These results confirm and expand the previously reported upregulation of Cathepsin S in these mice [29]. Both calpains and cathepsins belong to the papain superfamily of cysteine proteases that are primarily localized in lysosomes and are critical for intracellular protein catabolism. Calpain-mediated extralysosomal release of cathepsins is a critical event in necrosis, and cathepsin inhibitors show strong neuroprotection in models of acute neuronal insults [30,31]. Thus, we propose that neurodegeneration in these mice is a result of a progressive neuronal calpain-cathepsin system activation over time. How does loss of PS function lead to induction of the calpain-cathepsin system? We offer two proposals that are not mutually exclusive. In the first, we suggest that in the absence of PS/γ-secretase activity, extremely high levels of membrane-tethered stubs derived from type 1 membrane proteins hyperaccumulate in endosomal-lysosomal compartments, hence "saturating" those proteases that would otherwise be necessary for maintaining intracellular homeostasis. In this setting, the induction of the calpain-cathepsin system would thus reflect a compensatory mechanism. This hypothesis is consistent with the model that a function of PS/γ-secretase is to serve as a membrane-bound proteosome that promotes intramembranous proteolysis in order to enhance clearance of membrane-tethered stubs derived from ectodomain shedding of type 1 membrane proteins [32]. Indeed, we have shown that cultured mammalian cells expressing dominant-negative PS1 variants harboring mutations at the critical aspartate 257 and 385 residues necessary for γ-secretase activity, exhibit unusual accumulations of distended intramembranous organelles resembling multivesicular bodies that are immunoreactive with antibodies specific for membrane-tethered stubs [33]. The second model to explain our findings is in the absence of PS expression, the cell surface levels of receptors and ion channels in either pre- or postsynaptic membranes is altered in manner that gives rise to inappropriate signaling events and alterations in calcium homeostasis. In support of this notion, we [33] and others [34] have shown that cells that express dominant-negative forms of PS1 or that lack *PSEN *alleles, respectively, exhibit elevated levels of a variety of type I membrane proteins and their membrane-tethered stubs.

Inhibition of PS/γ-secretase activity has been debated as a promising therapeutic strategy for treatment of Alzheimer's Disease [35,36]. In this context, our results offer a cautionary note and suggest that the preclinical evaluation of such agents should include close monitoring for signs of calpain-cathepsin system activation, and development of a peripheral tissue assays on the complement-cathepsin system expression as informative tools for assessment of potential adverse effects that may also be present is the CNS.

## Methods

### A. Experimental animals

To generate forebrain-specific PS conditional double knockout (PSKO) mice, we crossed floxed PS1 (fPS1), αCaMKII-Cre transgenic [19,37,38] and PS2-/- [39] mice together to obtain fPS1/fPS1;αCaMKII-Cre;PS2-/- mice. All experimental procedures were reviewed and approved by Institutional Animal Care and Use Committee (IACUC) of the University of Chicago (Protocol #s 71434 and 70958).

### B. Sample preparation and hybridization

Hippocampi and frontal cortices were rapidly dissected and frozen on dry ice and stored at -80°C until RNA isolation. Total RNA was isolated using the Trizol reagent. RNA quality was assessed using the Agilent Bioanalyzer. All samples reported an RNA Integrity Number (RIN) > 8.0. Reverse transcription, in vitro transcription and fragmentation were performed according to manufacturer's recommendation . FC and HC samples for microarray analysis were generated by pooling RNA from 3 animals from appropriate genotypes and ages. Samples were hybridized onto MG_430 mouse Affymetrix GeneChips. Microarrays were considered for use only if the average 3':5' ratio for GAPDH and actin did not exceed 1:1.2. Segmentation of scanned microarray images was performed by MAS5. Determination of expression levels and scaling were performed using gcRMA in GenePattern [40].

### C. Data analysis

#### Identification of differentially expressed genes

i) Comparison of all KO animals to matched WT regardless of age or brain region. We identified genes as differentially expressed in the PSKO samples showed a pairwise difference > 50% (|ALR| > 0.585) at p < 0.05 to the matched WT controls. In this comparison a matched pairwise comparison eliminates the effects that are due to robust differences in the developmental age and brain region.

ii) Comparison of all HC_KO _animals to matched HC_WT_. We identified genes as differentially expressed in the HC_KO _samples showed a pairwise difference > 50% (|ALR| > 0.585) at p < 0.05 in the matched WT controls. In this comparison a matched pairwise comparison eliminated the effects due to developmental age.

iii) Comparison of all FC_KO _animals to matched FC_WT_. The comparison was performed in a similar fashion to that described for the HC samples.

##### Pathway analyses

These analyses were performed on the whole dataset using GSEA 2.0 [41] and the preset pathways from BioCarta (ref). Enrichment in a pathway was considered significant if it reached a p < 0.01 at a q < 0.05 (FDR < 5%).

##### Correlations

Correlations were calculated using Pearson *r *value for the log_2 _ratios between the two compared conditions.

##### Clustering

Two-way clustering (sample and gene vectors) was performed on gcRMA generated log2-transformed expression levels using Euclidian distance in GSEA.

##### Data sharing

The gcRMA normalized microarray dataset, together with the previous PS1 KO and TG datasets [12-15] is available for download from . Forthermore, the data will be deposiet in a MIAME/MGED format into the NCBI Gene Expression Omnibus database at the time of publication.

### D. Western blots

A new set of control and experimental animals was used in this study. 100 ug of cortical brain homogenate was run on 12% SDS-PAGE under reducing conditions, transferred to nitrocellulose and probed for C1q (1:800). Membranes were stripped and re-probed for Cathepsin D (1:10,000) and tubulin (loading control). For each, membranes were blocked with 5% milk in tris-buffered saline with 0.1% tween-20 (TBST), incubated with primary antibody overnight at 4°C, washed and incubated in HRP-conjugated secondary antibody for 1 hour at room temperature. Blots were exposed using ECL substrate and a Chemidoc XRS imager (Bio-Rad). Cathepsin D antibody [42] was obtained from Dr. Ralph Nixon (New York University), while C1q antibody [43,44] was provided by Dr. Andrea J. Tenner (U of California at Irvine). Molecular weights of Ctsd species correlate best to active intermediate (48 kD) and mature heavy chain (34 kD) [45].

## Competing interests

SSS discloses that he is a paid Consultant of Neuropharma, Inc., Torrey Pines Therapeutics and Eisai Research Labs Inc, but does not a shareholder in any company that is a maker or owner of a FDA-regulated drug or device.

## Authors' contributions

All authors read and approved the final manuscript. KM and SSS designed the study and supervised all steps of the experiments. KM wrote the initial draft of the manuscript. KG carried out the molecular biology part of the studies, SHC and XZ generated the animals and harvested the brain tissue, PE and KM performed the statistical analyses, while EMN performed the Western blotting for C1q and Ctsd.

## Supplementary Material

Additional file 1 **Gene expression differences between PSKO and CNT mice regardless of age or brain region**. A gene probe was differentially expressed if it reported an > 50% change (|ALR| > 0.585) at a pairwise t-test p < 0.05 between all the PSKO and CNT samples. 72 genes were upregulated, while 41 genes showed reduction in the PSKO samples. The probes from this list are clustered in Additional File 2. Click here for file

Additional file 2 **Hierarchical clustering of genes that show differential expression between PSKO and CNT mice regardless of age or brain region**. The numerical data are presented in Additional File 1. Gene probes are clustered in rows (denoted by symbols and probe identifiers), microarrays are clustered in columns (denoted by sample identifiers). Each pixel represents a single, color-coded gene expression value form FC or HC form a PSKO or CNT mouse sample at five developmental ages. Shades of red correspond to the magnitude of expression increase; the intensity of blue corresponds to the magnitude of the reduction in gene transcripts. Note that the PSKO and CNT samples, regardless of developmental age or brain region, cluster on different ends of the dendrogram. Click here for file

Additional file 3 **Detailed information about the enriched pathways in the PSKO or CNTR mice**. This file is a composite of GSEA outputs of BioCarta analysis for individual pathways that showed enrichment in either the PSKO or CNTR mice. For detailed description of output format, consult Click here for file

Additional file 4 **Gene expression differences in the frontal cortex (FC) of PSKO and CNT mice**. A gene probe was differentially expressed if it reported > 50% change (|ALR| > 0.585) at a pairwise t-test p < 0.05 between the FC of the PSKO and CNT samples. 70 genes were upregulated, while only 3 genes showed reduction in the PSKO samples. The probes from this list are clustered in Figure 2. Click here for file

Additional file 5 **Gene expression differences in the hippocampus (HC) of PSKO and CNT mice**. A gene probe was differentially expressed if it reported > 50% change (|ALR| > 0.585) at a pairwise t-test p < 0.05 between the HC of the PSKO and CNT samples. 36 genes were upregulated, while 101 genes showed reduction in the PSKO samples. The probes from this list are clustered in Figure 3. Click here for file

Additional file 6 **Developmental expression of *Cathepsin *and *Complement *family members in FC and HC**. X axis denotes postnatal age, Y axis represent RMA-normalized log2 expression, each colored bar corresponds to a different gene probeset. Note that in both the FC and HC the expression of these genes is relatively steady during postnatal development. Click here for file
