# Supplementary material for: Molecular signatures of neurodegeneration in the cortex of PS1/PS2 double knockout mice
Source: Mol Neurodegener. 2008 Oct 3;3:14. doi: 10.1186/1750-1326-3-14 (PMC2569036; doi:10.1186/1750-1326-3-14)
Supplement: Additional file 1 — Gene expression differences between PSKO and CNT mice regardless of age or brain region. A gene probe was differentially expressed if it reported an > 50% change (|ALR| > 0.585) at a pairwise t-test p < 0.05 between all the PSKO and CNT samples. 72 genes were upregulated, while 41 genes showed reduction in the PSKO samples. The probes from this list are clustered in Additional File 2. [file 1750-1326-3-14-S1.pdf]

SUPPLEMENTAL MATERIAL 1. Differences between PS1PS2KO and CNT mice regardless of age or brain region (>50%, p<0.05)

Probe Set	Gene Title	Symbol	Unigene	NCBI ID	allWT	allKO	ALR all	ALR HC	ALR FC	pVal ALL	PrPval HC	PrPval FC
1417851_at	chemokine (C-X-C motif) ligand 13	Cxcl13	Mm.10116.1	AF030636	5.13	7.43	2.31	2.02	2.59	0.00452	0.13134	0.02250
1419202_at	cystatin F (leukocystatin)	Cst7	Mm.12965.1	NM_009977	4.75	6.72	1.97	1.41	2.53	0.00143	0.08791	0.00992
1417266_at	chemokine (C-C motif) ligand 6	Ccl6	Mm.137.1	BC002073	5.26	6.84	1.58	1.23	1.92	0.00025	0.04007	0.00373
1446181_at	expressed sequence C85699	C85699	Mm.172989.1	BG067253	4.13	5.62	1.49	1.96	1.01	0.01975	0.13849	0.06535
1420699_at	C-type lectin domain family 7, member a	Clec7a	Mm.132943.1	NM_020008	5.08	6.56	1.47	1.20	1.75	0.00100	0.06092	0.01402
1426509_s_at	glial fibrillary acidic protein	Gfap	Mm.1239.1	BB183081	8.93	10.36	1.43	1.13	1.73	0.00124	0.04247	0.02356
1418021_at	complement component 4B	C4b	Mm.16106.1	NM_009780	8.13	9.47	1.34	1.07	1.61	0.00507	0.11087	0.03941
1450792_at	TYRO protein tyrosine kinase binding protein	Tyrobp	Mm.46301.1	NM_011662	7.77	9.11	1.34	1.13	1.56	0.00088	0.06521	0.01056
1426508_at	glial fibrillary acidic protein	Gfap	Mm.1239.1	BB183081	9.28	10.60	1.32	0.98	1.67	0.00260	0.05941	0.03135
1448859_at	chemokine (C-X-C motif) ligand 13	Cxcl13	Mm.10116.1	AF030636	6.10	7.41	1.32	1.07	1.56	0.00426	0.13192	0.02315
1436996_x_at	lysozyme	Lyz	Mm.45436.2	AV066625	6.72	7.88	1.16	0.79	1.54	0.00803	0.15150	0.03980
1423547_at	lysozyme	Lyzs	Mm.45436.1	AW208566	7.05	8.16	1.11	0.71	1.51	0.00851	0.12182	0.04666
1417381_at	complement component 1, q subcomponent, alpha	C1qa	Mm.370.1	NM_007572	8.73	9.84	1.11	0.94	1.28	0.00180	0.09314	0.01369
1439426_x_at	lysozyme	Lyz	Mm.45436.3	AV058500	4.69	5.75	1.06	0.74	1.37	0.00311	0.07813	0.02788
1449164_at	CD68 antigen	Cd68	Mm.15819.1	BC021637	6.28	7.31	1.03	0.85	1.22	0.00342	0.09923	0.02970
1419128_at	integrin alpha X	Itgax	Mm.22378.1	NM_021334	5.02	6.02	1.00	0.57	1.43	0.00244	0.12644	0.00716
1460218_at	CD52 antigen	Cd52	Mm.24130.1	NM_013706	5.62	6.61	0.99	0.81	1.16	0.00108	0.07929	0.00912
1417063_at	complement component 1, q subcomponent, beta	C1qb	Mm.2570.1	NM_009777	8.45	9.41	0.97	0.84	1.10	0.00297	0.10032	0.02402
1437726_x_at	complement component 1, q subcomponent, beta	C1qb	Mm.2570.3	BB111335	5.79	6.75	0.96	0.94	0.98	0.00025	0.02432	0.01219
1452968_at	collagen triple helix repeat containing 1	Cthrc1	Mm.41556.1	AK003674	5.37	6.33	0.96	1.00	0.92	0.00030	0.02034	0.04006
1425519_a_at	CD74 antigen (invariant polypeptide of MHC class II)	Cd74	Mm.7043.1	BC003476	6.49	7.44	0.95	0.69	1.21	0.00850	0.16952	0.03817
1421792_s_at	triggering receptor expressed on myeloid cells 2	Trem2	Mm.195828.1	NM_031254	6.61	7.54	0.93	0.66	1.20	0.00288	0.12005	0.01573
1427076_at	macrophage expressed gene 1	Mpeg1	Mm.3999.1	L20315	7.17	8.10	0.93	0.85	1.01	0.00038	0.03121	0.01347
1449401_at	complement component 1, q subcomponent, C	C1qc	Mm.3453.1	NM_007574	8.45	9.38	0.93	0.79	1.07	0.00185	0.07523	0.02206
1449911_at	lymphocyte-activation gene 3	Lag3	Mm.4528.1	NM_008479	5.71	6.62	0.91	0.59	1.24	0.00267	0.12388	0.01152
1433935_at	expressed sequence AU020206	AU020206	Mm.200422.1	BI151331	5.24	6.14	0.90	0.74	1.05	0.00219	0.06900	0.02895
1429846_at	RIKEN cDNA 9030411K21 gene	9030411K21R	Mm.100469.1	BM250766	4.19	5.08	0.89	1.03	0.76	0.00464	0.02851	0.15935
1422903_at	lymphocyte antigen 86	Ly86	Mm.2639.1	NM_010745	7.44	8.31	0.87	0.60	1.13	0.00215	0.09559	0.01465
1417868_a_at	cathepsin Z	Ctsz	Mm.156919.1	NM_022325	7.44	8.30	0.86	0.81	0.90	0.00256	0.08309	0.02592
1440807_at	Membrane associated guanylate kinase 2	Magi2	Mm.132480.1	BB337886	4.17	5.03	0.86	1.05	0.66	0.03780	0.02008	0.40748
1451780_at	B-cell linker	Blnk	Mm.9749.1	AF068182	6.66	7.50	0.85	0.81	0.89	0.00379	0.08357	0.04229
1419483_at	complement component 3a receptor 1	C3ar1	Mm.2408.1	NM_009779	5.32	6.14	0.82	0.66	0.98	0.00251	0.05516	0.03805
1417870_x_at	cathepsin Z	Ctsz	Mm.156919.1	NM_022325	7.84	8.65	0.81	0.70	0.92	0.00369	0.11343	0.02486
1434819_at	beta galactoside alpha 2,6 sialyltransferase 2	St6gal2	Mm.83662.1	BB709312	6.17	6.98	0.81	1.17	0.45	0.03765	0.15352	0.06530
1457797_at	expressed sequence AI605517	AI605517	Mm.37805.1	AV340788	6.70	7.51	0.81	0.79	0.82	0.04990	0.24921	0.24132
1445111_at	---	AW548720	Mm.215518.1	AW548720	4.69	5.49	0.80	0.92	0.68	0.01468	0.12396	0.15998
1427351_s_at	immunoglobulin heavy chain 6 (heavy chain of IgM)	Igh-6	Mm.218842.1	BB226392	7.32	8.12	0.80	0.85	0.74	0.01885	0.14780	0.10140
1435290_x_at	histocompatibility 2, class II antigen A, alpha	H2-Aa	Mm.175310.2	BE688749	5.98	6.75	0.78	0.54	1.01	0.02613	0.33849	0.03616
1430096_at	RIKEN cDNA 2900017F05 gene	2900017F05R	Mm.17681.1	AK013544	5.28	6.05	0.78	1.23	0.32	0.03778	0.06260	0.34964
1416066_at	CD9 antigen	Cd9	Mm.2956.1	NM_007657	8.45	9.22	0.77	0.67	0.87	0.00160	0.06631	0.02275
1438200_at	sulfatase 1	Sulf1	Mm.45563.1	BB065799	6.33	7.08	0.75	1.24	0.26	0.02865	0.01863	0.28295
1416382_at	cathepsin C	Ctsc	Mm.684.1	NM_009982	6.44	7.17	0.73	0.56	0.90	0.00157	0.05315	0.02338
1442241_at	Serine/arginine-rich protein specific kinase 2	Srpk2	Mm.82655.1	BE979921	6.19	6.90	0.71	0.82	0.61	0.01075	0.11007	0.13599
1436905_x_at	lysosomal-associated protein transmembrane 5	Laptm5	Mm.4554.2	BB218107	5.76	6.48	0.71	0.69	0.73	0.00014	0.01123	0.01530
1428114_at	solute carrier family 14 (urea transporter), member 1	Slc14a1	Mm.33832.1	AW556396	6.06	6.76	0.71	0.44	0.98	0.00222	0.08049	0.01390
1422124_a_at	protein tyrosine phosphatase, receptor type, C	Ptprc	Mm.143846.1	NM_011210	4.94	5.65	0.71	0.61	0.81	0.00493	0.04972	0.07405
1448591_at	cathepsin S	Ctss	Mm.3619.1	NM_021281	9.00	9.70	0.70	0.50	0.90	0.00214	0.12149	0.00945
1454268_a_at	cytochrome b-245, alpha polypeptide	Cyba	Mm.448.2	AK018713	6.32	7.02	0.70	0.43	0.96	0.00208	0.06983	0.01453
1417869_s_at	cathepsin Z	Ctsz	Mm.156919.1	NM_022325	5.76	6.46	0.70	0.64	0.76	0.00157	0.04719	0.03422

1453620_at	cortactin binding protein 2	Cttnbp2	Mm.133159.1	AW488544	**7.67**	**8.36**	**0.69**	**0.81**	**0.57**	*0.02683*	*0.14380*	*0.23360*
1416714_at	interferon regulatory factor 8	Irf8	Mm.3182.1	BG069095	**5.45**	**6.14**	**0.69**	**0.44**	**0.94**	*0.00365*	*0.10040*	*0.02244*
1434366_x_at	complement component 1, q subcomponent, beta	C1qb	Mm.2570.2	AW227993	**6.71**	**7.40**	**0.69**	**0.60**	**0.78**	*0.00113*	*0.03548*	*0.03044*
1429135_at	RIKEN cDNA 1110059M19 gene	1110059M19R	Mm.23496.1	AV015858	**5.41**	**6.09**	**0.69**	**1.03**	**0.34**	*0.02029*	*0.03778*	*0.25963*
1436107_at	LSM8 homolog, U6 small nuclear RNA associated	Lsm8	Mm.133137.1	AV167707	**5.03**	**5.71**	**0.68**	**0.79**	**0.57**	*0.04282*	*0.21509*	*0.23001*
1420697_at	solute carrier family 15, member 3	Slc15a3	Mm.218870.1	NM_023044	**4.39**	**5.07**	**0.68**	**0.49**	**0.87**	*0.00648*	*0.04774*	*0.06500*
1446772_at	---	BB453864	Mm.215970.1	BB453864	**4.12**	**4.79**	**0.67**	**0.87**	**0.47**	*0.00352*	*0.01143*	*0.12967*
1437874_s_at	hexosaminidase B	Hexb	Mm.219675.2	AV225808	**8.62**	**9.29**	**0.67**	**0.58**	**0.75**	*0.00171*	*0.07442*	*0.02038*
1426808_at	lectin, galactose binding, soluble 3	Lgals3	Mm.2970.1	X16834	**4.99**	**5.65**	**0.66**	**0.48**	**0.83**	*0.00605*	*0.00033*	*0.08579*
1419004_s_at	B-cell leukemia/lymphoma 2 related protein A1	Bcl2a1	Mm.196731.1	L16462	**5.69**	**6.34**	**0.65**	**0.45**	**0.86**	*0.00561*	*0.08845*	*0.04303*
1419132_at	toll-like receptor 2	Tlr2	Mm.87596.1	NM_011905	**5.11**	**5.76**	**0.65**	**0.42**	**0.89**	*0.00867*	*0.11926*	*0.04823*
1418340_at	Fc receptor, IgE, high affinity I, gamma polypeptide	Fcer1g	Mm.22673.1	NM_010185	**6.88**	**7.52**	**0.65**	**0.47**	**0.82**	*0.00215*	*0.07978*	*0.02018*
1448118_a_at	cathepsin D	Ctsd	Mm.2147.1	NM_009983	**10.56**	**11.20**	**0.64**	**0.60**	**0.69**	*0.00147*	*0.05952*	*0.02417*
1427329_a_at	immunoglobulin heavy chain 6 (heavy chain of IgM)	Igh-6	Mm.28362.2	AI326478	**5.94**	**6.58**	**0.64**	**0.65**	**0.62**	*0.01051*	*0.12194*	*0.07176*
1440637_at	Intersectin 1 (SH3 domain protein 1A)	Itsn1	Mm.103075.1	BG074656	**6.12**	**6.76**	**0.63**	**0.75**	**0.52**	*0.01805*	*0.11570*	*0.20112*
1450678_at	integrin beta 2	Itgb2	Mm.1137.1	NM_008404	**5.90**	**6.53**	**0.63**	**0.44**	**0.82**	*0.00512*	*0.13998*	*0.02652*
1456440_s_at	ST8 a-N-acetyl-neuraminide alpha-2,8-sialyltransf 6	St8sia6	Mm.24267.2	AV375081	**5.33**	**5.96**	**0.63**	**0.72**	**0.53**	*0.02041*	*0.00816*	*0.31737*
1456655_at	16 days neonate cerebellum cDNA	BM231698	Mm.69077.1	BM231698	**6.96**	**7.57**	**0.62**	**0.68**	**0.56**	*0.01113*	*0.06220*	*0.20169*
1448748_at	pleckstrin	Plek	Mm.98232.1	AF181829	**6.29**	**6.90**	**0.61**	**0.54**	**0.69**	*0.00499*	*0.08747*	*0.05320*
1418365_at	cathepsin H	Ctsh	Mm.2277.1	NM_007801	**6.45**	**7.06**	**0.61**	**0.52**	**0.71**	*0.00174*	*0.02609*	*0.04492*
1417268_at	CD14 antigen	Cd14	Mm.3460.1	NM_009841	**5.83**	**6.43**	**0.61**	**0.40**	**0.81**	*0.00008*	*0.01818*	*0.00027*
1419482_at	complement component 3a receptor 1	C3ar1	Mm.2408.1	NM_009779	**5.31**	**5.91**	**0.60**	**0.28**	**0.91**	*0.02177*	*0.34027*	*0.03940*
1418989_at	cathepsin E	Ctse	Mm.33671.1	NM_007799	**4.96**	**5.55**	**0.59**	**0.30**	**0.89**	*0.04693*	*0.37190*	*0.09581*
1450249_s_at	kinesin family member 5	Kif5	Mm.30355.1	NM_008447	**9.84**	**9.26**	**-0.59**	**-0.74**	**-0.43**	*0.00679*	*0.07550*	*0.02867*
1430144_at	RIKEN cDNA 5830411G16 gene	5830411G16R	Mm.105343.1	BB815600	**5.84**	**5.25**	**-0.59**	**-0.73**	**-0.45**	*0.00053*	*0.01204*	*0.00490*
1445061_at	Protocadherin 9	Pcdh9	Mm.155985.1	BM941356	**5.38**	**4.78**	**-0.59**	**-0.74**	**-0.45**	*0.00315*	*0.02633*	*0.10991*
1445631_at	---	BE865226	Mm.156043.1	BE865226	**7.89**	**7.29**	**-0.60**	**-0.99**	**-0.22**	*0.02380*	*0.01433*	*0.20587*
1445081_at	RIKEN cDNA A930041I02 gene	A930041I02R	Mm.204836.1	BB335888	**4.92**	**4.31**	**-0.61**	**-0.82**	**-0.40**	*0.04799*	*0.09968*	*0.41230*
1428484_at	oxysterol binding protein-like 3	Osbpl3	Mm.31024.1	AK004768	**7.69**	**7.08**	**-0.61**	**-0.75**	**-0.47**	*0.02787*	*0.11734*	*0.27315*
1430597_at	janus kinase and microtubule interacting protein 1	Jakmip1	Mm.159667.1	BB206614	**6.40**	**5.78**	**-0.61**	**-0.77**	**-0.45**	*0.01141*	*0.07705*	*0.16765*
1457142_at	EF hand calcium binding protein 1	Efcbp1	Mm.131089.1	BB667731	**6.88**	**6.26**	**-0.61**	**-0.99**	**-0.23**	*0.01915*	*0.01058*	*0.11194*
1440318_at	WD repeat domain 35	Wdr35	Mm.131476.1	BB475816	**6.73**	**6.12**	**-0.62**	**-0.96**	**-0.27**	*0.02358*	*0.04541*	*0.20028*
1458314_at	Solute carrier family 1 (glial-glutamate transporter), 2	Slc1a2	Mm.101872.1	AW491006	**5.17**	**4.55**	**-0.62**	**-0.31**	**-0.93**	*0.02922*	*0.20100*	*0.08620*
1443597_at	cofactor required for Sp1 transcriptional activation 2	Crsp2	Mm.187323.1	BB463128	**5.88**	**5.27**	**-0.62**	**-0.94**	**-0.30**	*0.01610*	*0.01351*	*0.25133*
1447513_at	potassium voltage-gated channel, Shal, member 3	Kcnd3	Mm.54226.1	BE957136	**5.42**	**4.80**	**-0.62**	**-0.98**	**-0.26**	*0.02069*	*0.02902*	*0.17797*
1459826_at	Potassium voltage-gated channel, subfamily Q, 2	Kcnq2	Mm.131914.1	BB264132	**6.53**	**5.91**	**-0.62**	**-1.00**	**-0.24**	*0.02561*	*0.03962*	*0.11719*
1457756_at	zinc finger protein 192	Zfp192	Mm.151515.1	BB483373	**5.15**	**4.53**	**-0.62**	**-0.70**	**-0.54**	*0.00573*	*0.11085*	*0.05755*
1429517_at	zinc finger, FYVE domain containing 20	Zfyve20	Mm.26129.1	BC017622	**5.66**	**5.04**	**-0.63**	**-0.87**	**-0.38**	*0.00844*	*0.00014*	*0.23081*
1431371_at	RIKEN cDNA 9030411M13 gene	9030411M13R	Mm.97440.1	BQ228391	**5.19**	**4.56**	**-0.63**	**-0.75**	**-0.51**	*0.00613*	*0.08545*	*0.08782*
1431196_at	ATPase, Ca++-sequestering	Atp2c1	Mm.153302.1	BG296252	**7.44**	**6.80**	**-0.64**	**-1.11**	**-0.18**	*0.03415*	*0.01496*	*0.20644*
1452418_at	RIKEN cDNA 1200016E24 gene	1200016E24R	Mm.171545.1	BF719154	**8.44**	**7.79**	**-0.65**	**-0.70**	**-0.59**	*0.00925*	*0.07114*	*0.11799*
1455960_at	multiple EGF-like-domains 9	Megf9	Mm.28686.3	BB483642	**7.16**	**6.51**	**-0.66**	**-1.06**	**-0.26**	*0.02330*	*0.03328*	*0.10127*
1417432_a_at	guanine nucleotide binding protein, beta 1	Gnb1	Mm.2344.1	NM_008142	**9.30**	**8.64**	**-0.66**	**-0.63**	**-0.69**	*0.00689*	*0.09981*	*0.06402*
1445473_at	DIRAS family, GTP-binding RAS-like 2	Diras2	Mm.123719.1	BB229114	**8.68**	**8.01**	**-0.67**	**-0.91**	**-0.43**	*0.01493*	*0.04776*	*0.24360*
1459317_at	ankyrin 2, brain	Ank2	Mm.208496.1	BB280924	**6.36**	**5.68**	**-0.68**	**-0.81**	**-0.54**	*0.00157*	*0.03391*	*0.05471*
1435770_at	thioredoxin domain containing 13	Txndc13	Mm.44400.1	BB215068	**8.05**	**7.37**	**-0.68**	**-1.14**	**-0.22**	*0.03786*	*0.04575*	*0.26496*
1444139_at	DNA-damage-inducible transcript 4-like	Ddit4l	Mm.205420.1	BG797099	**7.15**	**6.47**	**-0.68**	**-0.60**	**-0.76**	*0.00392*	*0.03772*	*0.10333*
1418610_at	solute carrier family 17, member 6	Slc17a6	Mm.70945.1	NM_080853	**6.52**	**5.84**	**-0.68**	**-0.78**	**-0.59**	*0.00014*	*0.01017*	*0.01269*
1447222_at	heat shock protein 12A	Hspa12a	Mm.86619.1	AI854582	**7.00**	**6.31**	**-0.68**	**-1.11**	**-0.25**	*0.01935*	*0.01092*	*0.05581*
1456827_at	expressed sequence AA987161	AA987161	Mm.138283.1	BB131790	**5.13**	**4.45**	**-0.68**	**-0.95**	**-0.42**	*0.00508*	*0.02914*	*0.04150*
1437257_at	WD repeat domain 47	Wdr47	Mm.125895.1	BB344753	**7.67**	**6.97**	**-0.70**	**-1.15**	**-0.24**	*0.04503*	*0.08890*	*0.05026*
1444141_at	sorting nexin 13	Snx13	Mm.213991.1	BB828008	**5.53**	**4.83**	**-0.71**	**-1.11**	**-0.30**	*0.04571*	*0.11210*	*0.21738*

1439517_at	myb-like, SWIRM and MPN domains 1	Mysm1	Mm.202463.1	BB660695	**6.16**	**5.42**	**-0.74**	**-1.05**	**-0.43**	*0.00762*	*0.03639*	*0.04338*
1454696_at	guanine nucleotide binding protein, beta 1	Gnb1	Mm.200776.1	BG071068	**11.05**	**10.31**	**-0.75**	**-0.52**	**-0.97**	*0.00583*	*0.06708*	*0.05214*
1453787_at	thioredoxin domain containing 13	Txndc13	Mm.156901.1	AK015667	**8.39**	**7.61**	**-0.78**	**-1.27**	**-0.28**	*0.02433*	*0.02602*	*0.07444*
1431254_at	kelch repeat and BTB (POZ) domain containing 11	Kbtbd11	Mm.153428.1	BG807055	**6.93**	**6.15**	**-0.78**	**-0.97**	**-0.59**	*0.01026*	*0.07136*	*0.16856*
1446652_at	RALBP1 associated protein 2	Reps2	Mm.151793.1	BB547008	**6.67**	**5.88**	**-0.79**	**-1.08**	**-0.50**	*0.00899*	*0.07116*	*0.02248*
1444298_at	RIKEN cDNA A130090K04 gene	A130090K04R	Mm.122667.1	BB703415	**6.60**	**5.81**	**-0.79**	**-1.06**	**-0.52**	*0.01443*	*0.07289*	*0.18248*
1459430_at	G protein-coupled receptor 158	Gpr158	Mm.209128.1	BB429778	**7.42**	**6.62**	**-0.81**	**-1.10**	**-0.51**	*0.00407*	*0.00783*	*0.10519*
1444001_at	RIKEN cDNA C230082I21 gene	C230082I21R	Mm.39545.1	AW488249	**8.99**	**8.05**	**-0.94**	**-1.48**	**-0.40**	*0.02240*	*0.04672*	*0.06925*
1432646_a_at	predicted gene, EG667653	EG667653	Mm.15985.3	BE859789	**8.89**	**7.91**	**-0.97**	**-1.48**	**-0.47**	*0.01130*	*0.05039*	*0.00798*
1431214_at	hypothetical gene LOC433762	LOC433762	Mm.157900.1	BG297038	**8.49**	**7.46**	**-1.03**	**-1.13**	**-0.93**	*0.00466*	*0.07314*	*0.05397*
1456911_at	CLIP associating protein 2	Clasp2	Mm.214961.1	BB831639	**7.42**	**6.30**	**-1.13**	**-1.25**	**-1.00**	*0.03548*	*0.15935*	*0.26848*
1431213_a_at	hypothetical gene LOC433762	LOC433762	Mm.157900.1	BG297038	**9.44**	**8.27**	**-1.17**	**-1.38**	**-0.96**	*0.00474*	*0.06365*	*0.05443*
