# Supplementary figures and images for: Molecular signatures of neurodegeneration in the cortex of PS1/PS2 double knockout mice
Source: Mol Neurodegener. 2008 Oct 3;3:14. doi: 10.1186/1750-1326-3-14 (PMC2569036; doi:10.1186/1750-1326-3-14)

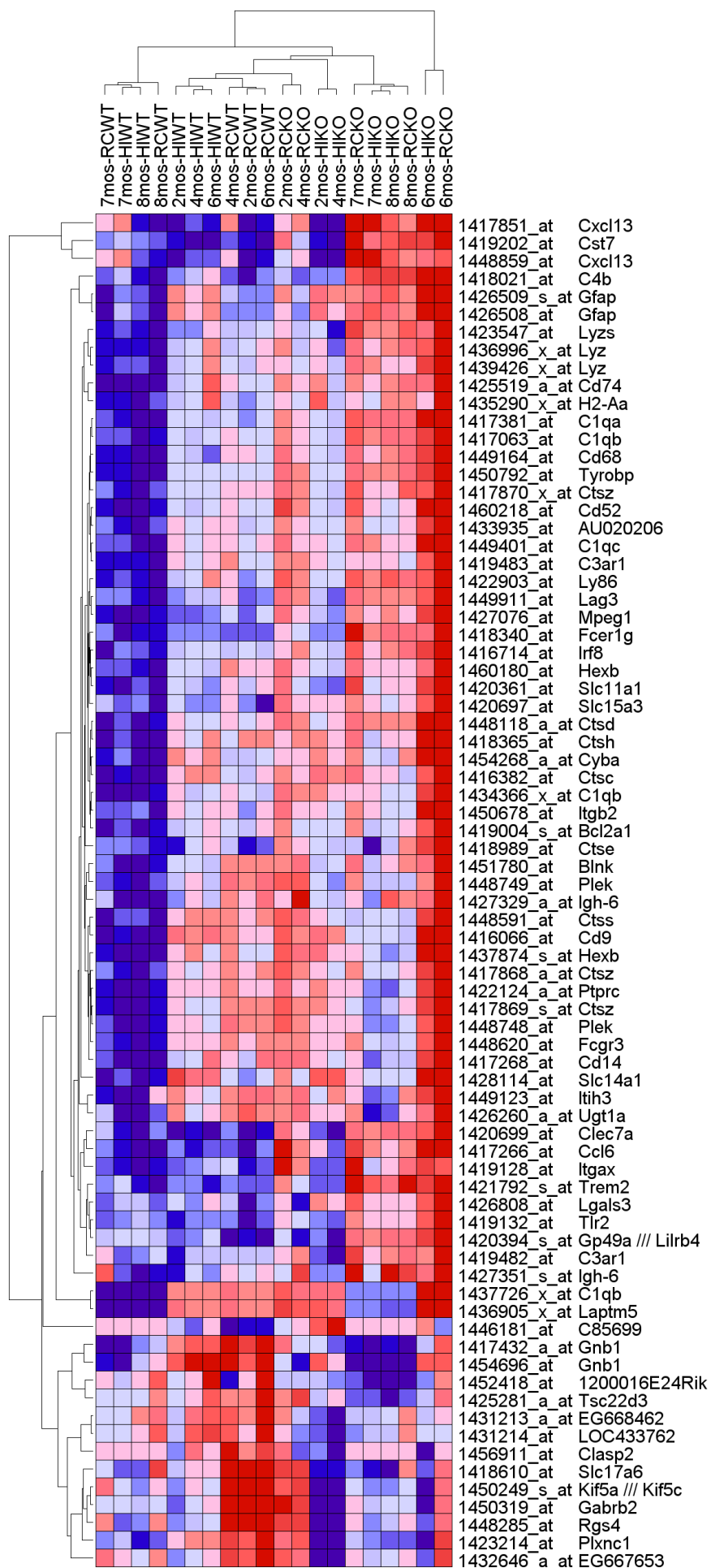

Supplement: Additional file 2 — Hierarchical clustering of genes that show differential expression between PSKO and CNT mice regardless of age or brain region. The numerical data are presented in Additional File 1. Gene probes are clustered in rows (denoted by symbols and probe identifiers), microarrays are clustered in columns (denoted by sample identifiers). Each pixel represents a single, color-coded gene expression value form FC or HC form a PSKO or CNT mouse sample at five developmental ages. Shades of red correspond to the magnitude of expression increase; the intensity of blue corresponds to the magnitude of the reduction in gene transcripts. Note that the PSKO and CNT samples, regardless of developmental age or brain region, cluster on different ends of the dendrogram. [file 1750-1326-3-14-S2.pdf]

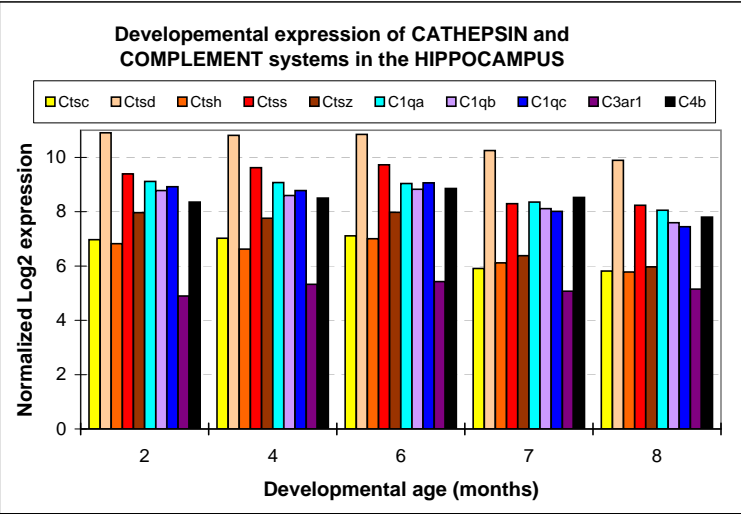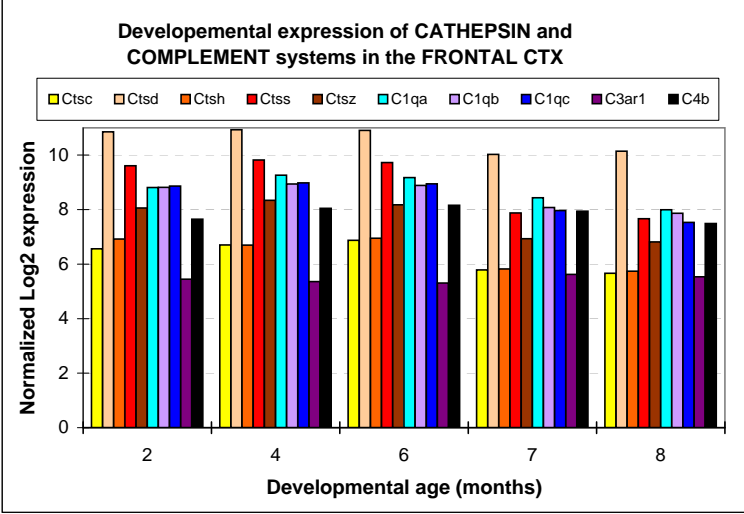

Supplement: Additional file 6 — Developmental expression of Cathepsin and Complement family members in FC and HC. X axis denotes postnatal age, Y axis represent RMA-normalized log2 expression, each colored bar corresponds to a different gene probeset. Note that in both the FC and HC the expression of these genes is relatively steady during postnatal development. [file 1750-1326-3-14-S6.pdf]
