# Supplementary material for: Molecular signatures of neurodegeneration in the cortex of PS1/PS2 double knockout mice
Source: Mol Neurodegener. 2008 Oct 3;3:14. doi: 10.1186/1750-1326-3-14 (PMC2569036; doi:10.1186/1750-1326-3-14)
Supplement: Additional file 3 — Detailed information about the enriched pathways in the PSKO or CNTR mice. This file is a composite of GSEA outputs of BioCarta analysis for individual pathways that showed enrichment in either the PSKO or CNTR mice. For detailed description of output format, consult [file 1750-1326-3-14-S3.pdf]

Table: Gene sets enriched in phenotype **Wild-type (10 samples)** **[plain text format]**

	GS follow link to MSigDB	GS DETAILS	SIZE	ES	NES	NOM p-val	FDR q-val	FWER p-val	RANK AT MAX	LEADING EDGE
1	GABAPATHWAY	Details ...	11	0.68	1.59	0.047	0.147	0.179	1904	tags=36%, list=9%, signal=40%
2	AGPCRPATHWAY	Details ...	10	0.67	1.45	0.019	0.244	0.379	2908	tags=30%, list=13%, signal=35%
3	IL12PATHWAY	Details ...	18	0.37	1.22	0.148	1.000	0.919	2760	tags=33%, list=13%, signal=38%
4	MPRPATHWAY	Details ...	19	0.46	1.11	0.355	1.000	0.991	951	tags=11%, list=4%, signal=11%
5	CHREBPPATHWAY	Details ...	15	0.42	1.08	0.393	1.000	0.993	2908	tags=20%, list=13%, signal=23%
6	GCRPATHWAY	Details ...	16	0.46	1.08	0.397	1.000	0.993	3535	tags=25%, list=16%, signal=30%
7	EDG1PATHWAY	Details ...	23	0.48	1.01	0.626	1.000	0.998	3535	tags=30%, list=16%, signal=36%
8	IL3PATHWAY	Details ...	14	0.30	0.98	0.434	1.000	0.999	3750	tags=29%, list=17%, signal=34%
9	CCR3PATHWAY	Details ...	20	0.34	0.94	0.677	1.000	1.000	30	tags=5%, list=0%, signal=5%
10	NO2IL12PATHWAY	Details ...	13	0.39	0.93	0.621	1.000	1.000	3797	tags=38%, list=17%, signal=47%
11	RBPATHWAY	Details ...	11	0.44	0.93	0.691	1.000	1.000	1457	tags=18%, list=7%, signal=19%
12	BIOPEPTIDESPATHWAY	Details ...	37	0.30	0.89	0.673	1.000	1.000	5454	tags=32%, list=25%, signal=43%

13	NOS1PATHWAY	Details ...	19	0.38	0.88	0.726	1.000	1.000	2542	tags=16%, list=12%, signal=18%
14	PAR1PATHWAY	Details ...	18	0.38	0.88	0.687	1.000	1.000	3535	tags=22%, list=16%, signal=26%
15	ACHPATHWAY	Details ...	13	0.41	0.88	0.715	1.000	1.000	1083	tags=15%, list=5%, signal=16%
16	CACAMPATHWAY	Details ...	13	0.49	0.88	0.788	1.000	1.000	5454	tags=38%, list=25%, signal=51%
17	ATRBRCAPATHWAY	Details ...	19	0.36	0.85	0.704	1.000	1.000	3631	tags=32%, list=17%, signal=38%
18	DREAMPATHWAY	Details ...	11	0.41	0.83	0.729	1.000	1.000	1783	tags=18%, list=8%, signal=20%
19	ACTINYPATHWAY	Details ...	16	0.34	0.80	0.752	1.000	1.000	2693	tags=19%, list=12%, signal=21%
20	SPPAPATHWAY	Details ...	19	0.32	0.74	0.762	1.000	1.000	30	tags=5%, list=0%, signal=5%
21	HCMVPATHWAY		14	0.38	0.72	0.780	1.000	1.000	13530	tags=100%, list=62%, signal=262%
22	P53HYPOXIAPATHWAY		18	0.29	0.68	0.902	1.000	1.000	2752	tags=22%, list=13%, signal=25%
23	CD40PATHWAY		12	0.32	0.64	0.883	1.000	1.000	6302	tags=50%, list=29%, signal=70%
24	TALL1PATHWAY		12	0.24	0.60	0.953	1.000	1.000	2173	tags=17%, list=10%, signal=18%
25	INSULINPATHWAY		19	0.27	0.60	0.900	1.000	1.000	3750	tags=32%, list=17%, signal=38%

26	GPCRPATHWAY		33	0.25	0.60	0.894	1.000	1.000	2908	tags=12%, list=13%, signal=14%
27	GLEEVECPATHWAY		21	0.27	0.59	0.912	1.000	1.000	4318	tags=29%, list=20%, signal=36%
28	BADPATHWAY		19	0.26	0.58	0.944	1.000	1.000	3535	tags=21%, list=16%, signal=25%
29	ARFPATHWAY		12	0.26	0.56	0.937	1.000	1.000	5152	tags=42%, list=24%, signal=54%
30	CELLCYCLEPATHWAY		20	0.28	0.56	0.851	1.000	1.000	4418	tags=35%, list=20%, signal=44%
31	CARM1PATHWAY		11	0.27	0.55	0.955	1.000	1.000	15950	tags=100%, list=73%, signal=368%
32	SALMONELLAPATHWAY		10	0.26	0.54	0.983	1.000	1.000	16257	tags=100%, list=74%, signal=388%
33	IL7PATHWAY		15	0.21	0.53	0.977	1.000	1.000	17396	tags=100%, list=79%, signal=487%
34	RASPATHWAY		21	0.19	0.48	0.904	1.000	1.000	17710	tags=100%, list=81%, signal=523%
35	RARRXRPATHWAY		14	0.19	0.45	0.965	1.000	1.000	1009	tags=7%, list=5%, signal=7%
36	G2PATHWAY		21	0.21	0.44	0.980	1.000	1.000	3462	tags=19%, list=16%, signal=23%
37	VEGFPATHWAY		24	0.19	0.44	0.939	1.000	1.000	5843	tags=38%, list=27%, signal=51%
38	CREBPATHWAY		25	0.18	0.40	0.971	1.000	1.000	15488	tags=96%, list=71%, signal=328%

39	CHEMICALPATHWAY	20	0.17	0.38	0.981	1.000	1.000	6002	tags=35%, list=27%, signal=48%
40	IGF1RPATHWAY	14	0.18	0.37	0.982	1.000	1.000	3750	tags=21%, list=17%, signal=26%
41	RACCYCDPATHWAY	20	0.17	0.36	0.975	1.000	1.000	18204	tags=100%, list=83%, signal=593%
42	PTENPATHWAY	16	0.15	0.29	0.998	1.000	1.000	3750	tags=19%, list=17%, signal=23%
43	PYK2PATHWAY	27	0.11	0.27	1.000	1.000	1.000	6055	tags=26%, list=28%, signal=36%

Table: Gene sets enriched in phenotype **KO (10 samples)** **[plain text format]**

	GS follow link to MSigDB	GS DETAILS	SIZE	ES	NES	NOM p-val	FDR q-val	FWER p-val	RANK AT MAX	LEADING EDGE
1	NKCELLSPATHWAY	Details ...	15	-0.50	-1.70	0.025	0.139	0.084	4461	tags=33%, list=20%, signal=42%
2	EXTRINSICPATHWAY	Details ...	12	-0.71	-1.69	0.015	0.082	0.097	5458	tags=75%, list=25%, signal=100%
3	TOB1PATHWAY	Details ...	14	-0.73	-1.63	0.004	0.087	0.134	1018	tags=36%, list=5%, signal=37%
4	CTLA4PATHWAY	Details ...	12	-0.60	-1.50	0.056	0.234	0.304	322	tags=17%, list=1%, signal=17%
5	PLCEPATHWAY	Details ...	10	-0.64	-1.47	0.054	0.242	0.352	297	tags=10%, list=1%, signal=10%
6	VITCBPATHWAY	Details ...	10	-0.51	-1.45	0.039	0.228	0.377	3392	tags=30%, list=15%, signal=35%
7	LAIRPATHWAY	Details ...	10	-0.67	-1.45	0.046	0.198	0.380	2902	tags=50%, list=13%, signal=58%
8	INTRINSICPATHWAY	Details ...	20	-0.58	-1.45	0.021	0.178	0.390	5458	tags=55%, list=25%, signal=73%
9	MCALPAINPATHWAY	Details ...	22	-0.53	-1.44	0.006	0.179	0.426	595	tags=14%, list=3%, signal=14%
10	ACE2PATHWAY	Details ...	10	-0.56	-1.43	0.097	0.171	0.443	3945	tags=50%, list=18%, signal=61%
11	TGFBPATHWAY	Details ...	13	-0.67	-1.42	0.010	0.164	0.458	2520	tags=38%, list=12%, signal=43%
12	ERYTHPATHWAY	Details ...	12	-0.48	-1.36	0.061	0.304	0.667	1018	tags=25%, list=5%, signal=26%
13	IL10PATHWAY	Details ...	12	-0.55	-1.35	0.066	0.311	0.685	1988	tags=25%, list=9%, signal=27%

14	NTHIPATHWAY	Details ...	19	-0.61	-1.35	0.041	0.291	0.689	217	tags=16%, list=1%, signal=16%
15	ALKPATHWAY	Details ...	32	-0.55	-1.34	0.014	0.299	0.711	1993	tags=22%, list=9%, signal=24%
16	D4GDIPATHWAY	Details ...	10	-0.68	-1.31	0.043	0.397	0.780	5136	tags=70%, list=23%, signal=91%
17	RAC1PATHWAY	Details ...	21	-0.59	-1.30	0.020	0.419	0.806	643	tags=19%, list=3%, signal=20%
18	DCPATHWAY	Details ...	20	-0.65	-1.29	0.162	0.484	0.838	192	tags=15%, list=1%, signal=15%
19	CCR5PATHWAY	Details ...	16	-0.63	-1.27	0.078	0.579	0.870	1534	tags=25%, list=7%, signal=27%
20	AMIPATHWAY	Details ...	17	-0.57	-1.27	0.069	0.569	0.873	322	tags=12%, list=1%, signal=12%
21	CSKPATHWAY		17	-0.57	-1.27	0.069	0.541	0.873	322	tags=12%, list=1%, signal=12%
22	PMLPATHWAY		11	-0.57	-1.22	0.212	0.819	0.940	4764	tags=45%, list=22%, signal=58%
23	ECMPATHWAY		19	-0.52	-1.20	0.274	0.974	0.959	4035	tags=26%, list=18%, signal=32%
24	NKTPATHWAY		24	-0.43	-1.14	0.218	1.000	0.980	1018	tags=13%, list=5%, signal=13%
25	RHOPATHWAY		27	-0.44	-1.14	0.351	1.000	0.981	2473	tags=19%, list=11%, signal=21%
26	FIBRINOLYSISPATHWAY		11	-0.52	-1.12	0.294	1.000	0.988	7458	tags=64%, list=34%, signal=96%
27	CASPASEPATHWAY		20	-0.47	-1.07	0.429	1.000	0.996	5136	tags=40%, list=23%, signal=52%

28	FCER1PATHWAY		36	-0.42	-1.07	0.573	1.000	0.996	4461	tags=25%, list=20%, signal=31%
29	IL4PATHWAY		10	-0.38	-1.06	0.387	1.000	0.996	9813	tags=80%, list=45%, signal=145%
30	GSK3PATHWAY		25	-0.44	-1.03	0.556	1.000	0.998	728	tags=8%, list=3%, signal=8%
31	TELPATHWAY		14	-0.46	-1.03	0.617	1.000	0.998	4764	tags=36%, list=22%, signal=46%
32	CDMACPATHWAY		15	-0.41	-1.02	0.544	1.000	0.999	2099	tags=20%, list=10%, signal=22%
33	IL6PATHWAY		20	-0.31	-0.99	0.546	1.000	1.000	4968	tags=30%, list=23%, signal=39%
34	IL1RPATHWAY		30	-0.38	-0.99	0.587	1.000	1.000	2520	tags=20%, list=12%, signal=23%
35	RELAPATHWAY		15	-0.40	-0.99	0.600	1.000	1.000	7667	tags=60%, list=35%, signal=92%
36	LONGEVITYPATHWAY		11	-0.55	-0.98	0.659	1.000	1.000	4149	tags=55%, list=19%, signal=67%
37	MEF2DPATHWAY		18	-0.41	-0.97	0.669	1.000	1.000	634	tags=11%, list=3%, signal=11%
38	NFKBPATHWAY		22	-0.36	-0.95	0.654	1.000	1.000	8441	tags=59%, list=39%, signal=96%
39	TOLLPATHWAY		31	-0.36	-0.95	0.637	1.000	1.000	6343	tags=32%, list=29%, signal=45%
40	P35ALZHEIMERSPATHWAY		10	-0.40	-0.92	0.704	1.000	1.000	563	tags=10%, list=3%, signal=10%
41	BCRPATHWAY		33	-0.35	-0.92	0.692	1.000	1.000	4461	tags=24%, list=20%, signal=30%

42	MTA3PATHWAY	13	-0.39	-0.92	0.679	1.000	1.000	2228	tags=15%, list=10%, signal=17%
43	HIFPATHWAY	11	-0.44	-0.89	0.717	1.000	1.000	8908	tags=73%, list=41%, signal=123%
44	HSP27PATHWAY	14	-0.31	-0.89	0.656	1.000	1.000	4634	tags=43%, list=21%, signal=54%
45	PPARAPATHWAY	48	-0.34	-0.89	0.694	1.000	1.000	2814	tags=17%, list=13%, signal=19%
46	UCALPAINPATHWAY	14	-0.35	-0.89	0.699	1.000	1.000	6204	tags=29%, list=28%, signal=40%
47	TIDPATHWAY	17	-0.30	-0.88	0.648	1.000	1.000	7535	tags=53%, list=34%, signal=81%
48	P38MAPKPATHWAY	36	-0.35	-0.86	0.701	1.000	1.000	4745	tags=25%, list=22%, signal=32%
49	ERKPATHWAY	28	-0.36	-0.86	0.732	1.000	1.000	4182	tags=36%, list=19%, signal=44%
50	TNFR1PATHWAY	26	-0.38	-0.85	0.689	1.000	1.000	6904	tags=46%, list=32%, signal=67%
51	ARAPPATHWAY	19	-0.32	-0.84	0.733	1.000	1.000	866	tags=11%, list=4%, signal=11%
52	TH1TH2PATHWAY	13	-0.36	-0.84	0.715	1.000	1.000	103	tags=8%, list=0%, signal=8%
53	INFLAMPATHWAY	24	-0.37	-0.84	0.745	1.000	1.000	3891	tags=33%, list=18%, signal=40%
54	TCRPATHWAY	41	-0.31	-0.84	0.700	1.000	1.000	1534	tags=10%, list=7%, signal=10%
55	CARDIACEGFPATHWAY	16	-0.35	-0.83	0.770	1.000	1.000	2981	tags=25%, list=14%, signal=29%

56	INTEGRINPATHWAY	33	-0.33	-0.81	0.713	1.000	1.000	4298	tags=21%, list=20%, signal=26%
57	PROTEASOMEPATHWAY	20	-0.33	-0.80	0.753	1.000	1.000	10640	tags=55%, list=49%, signal=107%
58	IL2PATHWAY	20	-0.27	-0.78	0.828	1.000	1.000	4496	tags=25%, list=21%, signal=31%
59	CDK5PATHWAY	11	-0.34	-0.77	0.790	1.000	1.000	1024	tags=9%, list=5%, signal=10%
60	VIPPATHWAY	25	-0.38	-0.77	0.836	1.000	1.000	2099	tags=12%, list=10%, signal=13%
61	CBLPATHWAY	12	-0.36	-0.77	0.804	1.000	1.000	2981	tags=25%, list=14%, signal=29%
62	GATA3PATHWAY	13	-0.30	-0.77	0.835	1.000	1.000	5751	tags=31%, list=26%, signal=42%
63	HIVNEFPATHWAY	51	-0.32	-0.76	0.683	1.000	1.000	5451	tags=31%, list=25%, signal=42%
64	KERATINOCYTEPATHWAY	40	-0.30	-0.76	0.755	1.000	1.000	6184	tags=28%, list=28%, signal=38%
65	NDKDYNAMINPATHWAY	17	-0.33	-0.76	0.792	1.000	1.000	634	tags=6%, list=3%, signal=6%
66	P53PATHWAY	14	-0.37	-0.76	0.827	1.000	1.000	4764	tags=29%, list=22%, signal=36%
67	IL17PATHWAY	10	-0.33	-0.75	0.905	1.000	1.000	322	tags=10%, list=1%, signal=10%
68	CFTRPATHWAY	10	-0.33	-0.74	0.812	1.000	1.000	1844	tags=10%, list=8%, signal=11%
69	MAPKPATHWAY	81	-0.29	-0.74	0.764	1.000	1.000	2408	tags=12%, list=11%, signal=14%

70	EIF4PATHWAY		23	-0.33	-0.73	0.729	1.000	1.000	1027	tags=9%, list=5%, signal=9%
71	STRESSPATHWAY		23	-0.31	-0.73	0.786	1.000	1.000	2125	tags=17%, list=10%, signal=19%
72	41BBPATHWAY		14	-0.29	-0.72	0.861	1.000	1.000	10066	tags=71%, list=46%, signal=132%
73	MITOCHONDRIAPATHWAY		19	-0.32	-0.71	0.785	1.000	1.000	5472	tags=32%, list=25%, signal=42%
74	NGFPATHWAY		17	-0.32	-0.70	0.810	1.000	1.000	4182	tags=24%, list=19%, signal=29%
75	FASPATHWAY		24	-0.32	-0.70	0.789	1.000	1.000	6761	tags=33%, list=31%, signal=48%
76	MYOSINPATHWAY		13	-0.33	-0.69	0.830	1.000	1.000	595	tags=15%, list=3%, signal=16%
77	GHPATHWAY		25	-0.29	-0.69	0.834	1.000	1.000	3173	tags=20%, list=14%, signal=23%
78	IL2RBPATHWAY		33	-0.27	-0.68	0.863	1.000	1.000	5793	tags=30%, list=26%, signal=41%
79	CXCR4PATHWAY		23	-0.31	-0.67	0.859	1.000	1.000	75	tags=4%, list=0%, signal=4%
80	IGF1PATHWAY		19	-0.31	-0.67	0.834	1.000	1.000	4182	tags=26%, list=19%, signal=33%
81	ARENRF2PATHWAY		13	-0.32	-0.66	0.877	1.000	1.000	1534	tags=15%, list=7%, signal=17%
82	EPOPATHWAY		17	-0.26	-0.66	0.907	1.000	1.000	4182	tags=24%, list=19%, signal=29%
83	ERK5PATHWAY		16	-0.34	-0.66	0.901	1.000	1.000	9281	tags=69%, list=42%, signal=119%

84	P27PATHWAY	11	-0.35	-0.65	0.860	1.000	1.000	1466	tags=9%, list=7%, signal=10%
85	FMLPPATHWAY	34	-0.25	-0.65	0.849	1.000	1.000	634	tags=6%, list=3%, signal=6%
86	PS1PATHWAY	13	-0.28	-0.63	0.858	1.000	1.000	2231	tags=15%, list=10%, signal=17%
87	WNTPATHWAY	24	-0.22	-0.61	0.844	1.000	1.000	6438	tags=29%, list=29%, signal=41%
88	STEMPATHWAY	11	-0.35	-0.61	0.893	1.000	1.000	4224	tags=36%, list=19%, signal=45%
89	G1PATHWAY	22	-0.31	-0.61	0.860	1.000	1.000	4764	tags=27%, list=22%, signal=35%
90	RANKLPATHWAY	12	-0.20	-0.58	0.966	1.000	1.000	3472	tags=17%, list=16%, signal=20%
91	ETSPATHWAY	16	-0.27	-0.58	0.920	1.000	1.000	1534	tags=13%, list=7%, signal=13%
92	HDACPATHWAY	28	-0.27	-0.57	0.933	1.000	1.000	783	tags=7%, list=4%, signal=7%
93	PGC1APATHWAY	22	-0.27	-0.57	0.940	1.000	1.000	1984	tags=9%, list=9%, signal=10%
94	CERAMIDEPATHWAY	21	-0.24	-0.56	0.840	1.000	1.000	10471	tags=67%, list=48%, signal=128%
95	CK1PATHWAY	15	-0.30	-0.56	0.865	1.000	1.000	3425	tags=13%, list=16%, signal=16%
96	ATMPATHWAY	17	-0.26	-0.55	0.924	1.000	1.000	6457	tags=41%, list=29%, signal=58%
97	TRKAPATHWAY	12	-0.26	-0.54	0.944	1.000	1.000	7536	tags=42%, list=34%, signal=64%

98	NFATPATHWAY	49	-0.20	-0.54	0.957	1.000	1.000	1290	tags=6%, list=6%, signal=6%
99	EGFPATHWAY	26	-0.22	-0.53	0.934	1.000	1.000	4182	tags=19%, list=19%, signal=24%
100	MTORPATHWAY	22	-0.22	-0.53	0.846	1.000	1.000	651	tags=5%, list=3%, signal=5%
101	PITX2PATHWAY	15	-0.23	-0.52	0.950	1.000	1.000	845	tags=7%, list=4%, signal=7%
102	TPOPATHWAY	22	-0.22	-0.51	0.936	1.000	1.000	3173	tags=14%, list=14%, signal=16%
103	CELL2CELLPATHWAY	12	-0.25	-0.51	0.954	1.000	1.000	3864	tags=17%, list=18%, signal=20%
104	PDGFPATHWAY	26	-0.21	-0.50	0.923	1.000	1.000	4182	tags=19%, list=19%, signal=24%
105	IGF1MTORPATHWAY	18	-0.24	-0.50	0.899	1.000	1.000	4334	tags=17%, list=20%, signal=21%
106	PTDINSPATHWAY	20	-0.22	-0.50	0.892	1.000	1.000	10061	tags=55%, list=46%, signal=102%
107	DEATHPATHWAY	28	-0.21	-0.49	0.953	1.000	1.000	6904	tags=39%, list=32%, signal=57%
108	METPATHWAY	35	-0.19	-0.47	0.965	1.000	1.000	2674	tags=11%, list=12%, signal=13%
109	CALCINEURINPATHWAY	18	-0.23	-0.46	0.920	1.000	1.000	634	tags=6%, list=3%, signal=6%
110	NO1PATHWAY	27	-0.19	-0.45	0.953	1.000	1.000	4429	tags=15%, list=20%, signal=19%
111	AKTPATHWAY	15	-0.22	-0.44	0.950	1.000	1.000	6184	tags=27%, list=28%, signal=37%

112	CYTOKINEPATHWAY	16	-0.25	-0.44	0.984	1.000	1.000	7535	tags=56%, list=34%, signal=86%
113	SHHPATHWAY	12	-0.24	-0.43	0.955	1.000	1.000	5480	tags=25%, list=25%, signal=33%
114	SPRYPATHWAY	18	-0.20	-0.43	0.959	1.000	1.000	4127	tags=17%, list=19%, signal=21%
115	CARM_ERPATHWAY	24	-0.20	-0.43	0.973	1.000	1.000	6682	tags=38%, list=31%, signal=54%
116	TNFR2PATHWAY	17	-0.18	-0.43	0.996	0.997	1.000	7434	tags=41%, list=34%, signal=62%
117	AT1RPATHWAY	33	-0.15	-0.39	0.967	0.998	1.000	4182	tags=12%, list=19%, signal=15%
118	CDC42RACPATHWAY	12	-0.16	-0.37	0.998	0.994	1.000	18357	tags=100%, list=84%, signal=619%

Table: GSEA Results Summary

Dataset	wt vs ko gsea_collapsed_to_symbols.wt vs ko cls file.cls#Wild-type_versus_KO
Phenotype	wt vs ko cls file.cls#Wild-type_versus_KO
Upregulated in class	Wild-type
GeneSet	GABAPATHWAY
Enrichment Score (ES)	0.67899907
Normalized Enrichment Score (NES)	1.5901968
Nominal p-value	0.046747968
FDR q-value	0.14741753
FWER p-Value	0.179

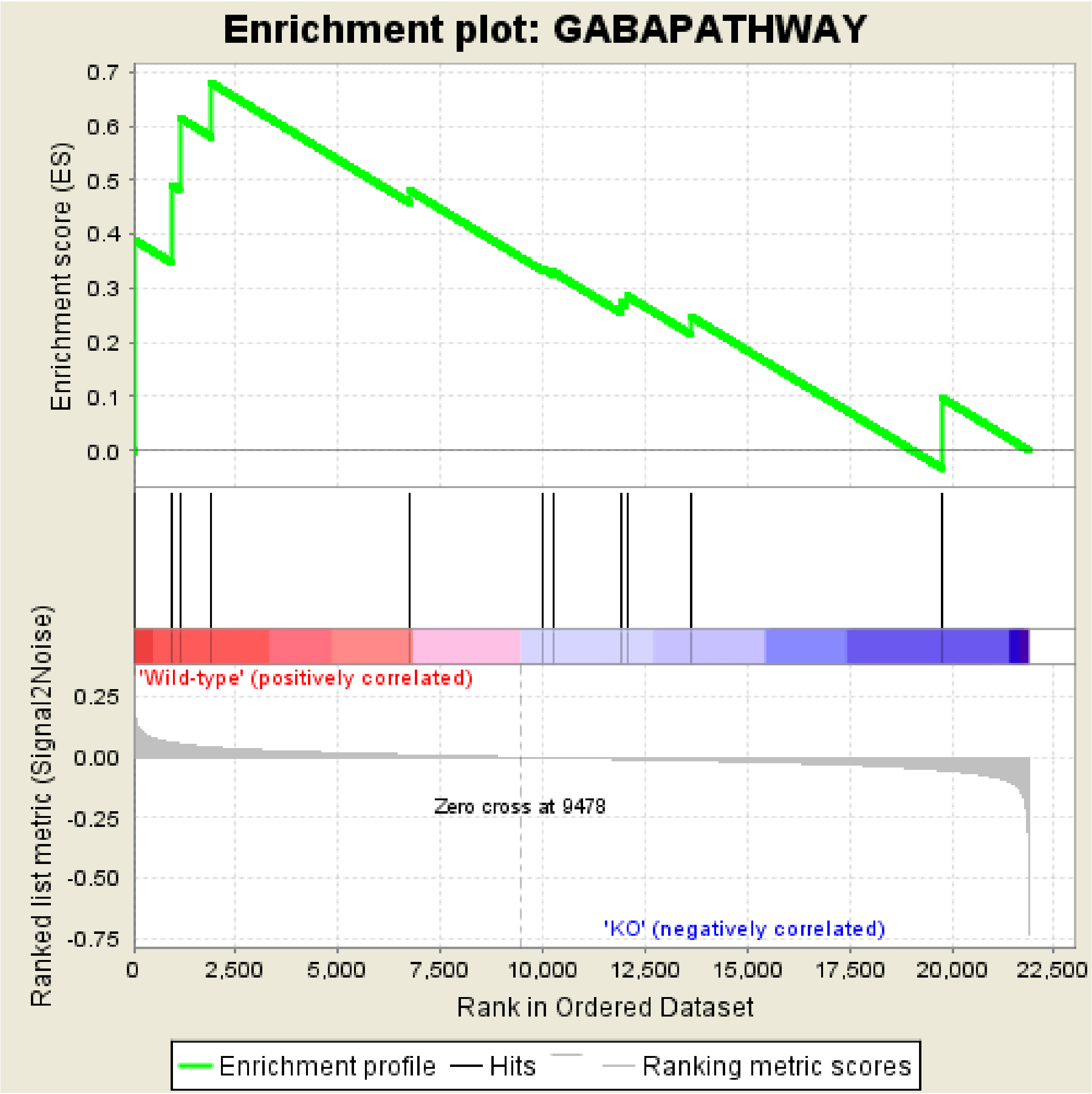

Fig 1: Enrichment plot: GABAPATHWAY
Profile of the Running ES Score & Positions of GeneSet Members on the Rank Ordered List

Table: GSEA details [plain text format]

	PROBE	GENE SYMBOL	GENE_TITLE	RANK IN GENE LIST	RANK METRIC SCORE	RUNNING ES	CORE ENRICHMENT

1	GABRA3	GABRA3<br>Entrez, Source, GeneCards	gamma-aminobutyric acid (GABA) A receptor, alpha 3	44	0.162	0.3879	Yes
2	GABRA2	GABRA2<br>Entrez, Source, GeneCards	gamma-aminobutyric acid (GABA) A receptor, alpha 2	942	0.060	0.4907	Yes
3	GABRA6	GABRA6<br>Entrez, Source, GeneCards	gamma-aminobutyric acid (GABA) A receptor, alpha 6	1118	0.055	0.6155	Yes
4	GABRA1	GABRA1<br>Entrez, Source, GeneCards	gamma-aminobutyric acid (GABA) A receptor, alpha 1	1904	0.041	0.6790	Yes
5	UBQLN1	UBQLN1<br>Entrez, Source, GeneCards	ubiquilin 1	6736	0.010	0.4816	No
6	NSF	NSF<br>Entrez, Source, GeneCards	N-ethylmaleimide-sensitive factor	9976	-0.002	0.3373	No
7	SRC	SRC<br>Entrez, Source, GeneCards	v-src sarcoma (Schmidt-Ruppin A-2) viral oncogene homolog (avian)	10238	-0.002	0.3311	No
8	GABRA4	GABRA4<br>Entrez, Source, GeneCards	gamma-aminobutyric acid (GABA) A receptor, alpha 4	11897	-0.008	0.2739	No
9	GPHN	GPHN<br>Entrez, Source, GeneCards	gephyrin	12050	-0.008	0.2867	No
10	GABARAP	GABARAP<br>Entrez, Source, GeneCards	GABA(A) receptor-associated protein	13645	-0.014	0.2467	No
11	GABRA5	GABRA5<br>Entrez, Source, GeneCards	gamma-aminobutyric acid (GABA) A receptor, alpha 5	19772	-0.054	0.0968	No

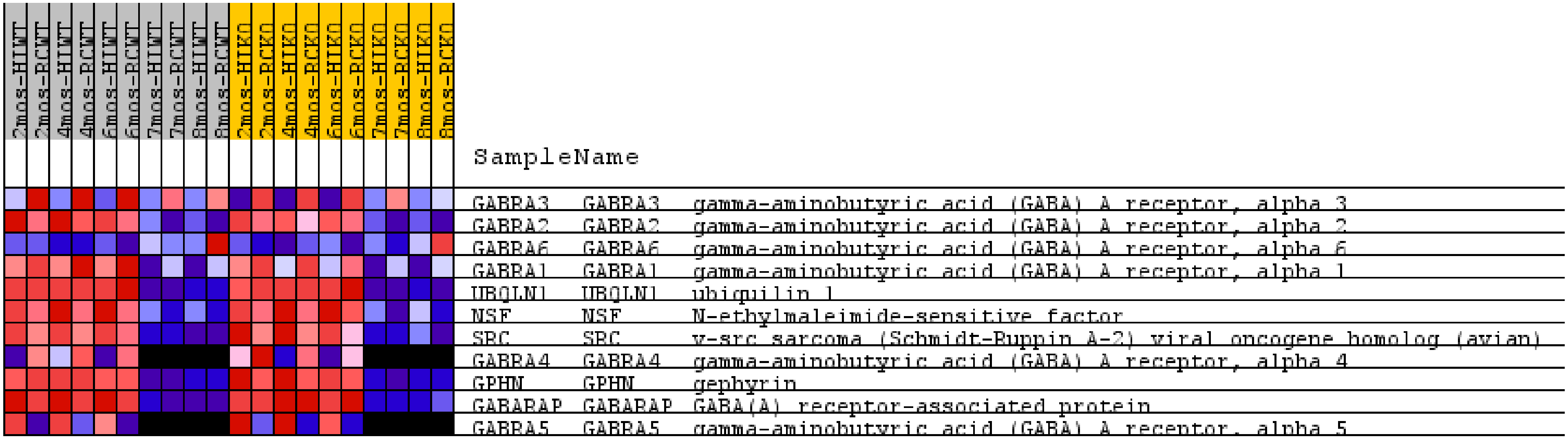

Fig 2: GABAPATHWAY
Blue-Pink O' Gram in the Space of the Analyzed GeneSet

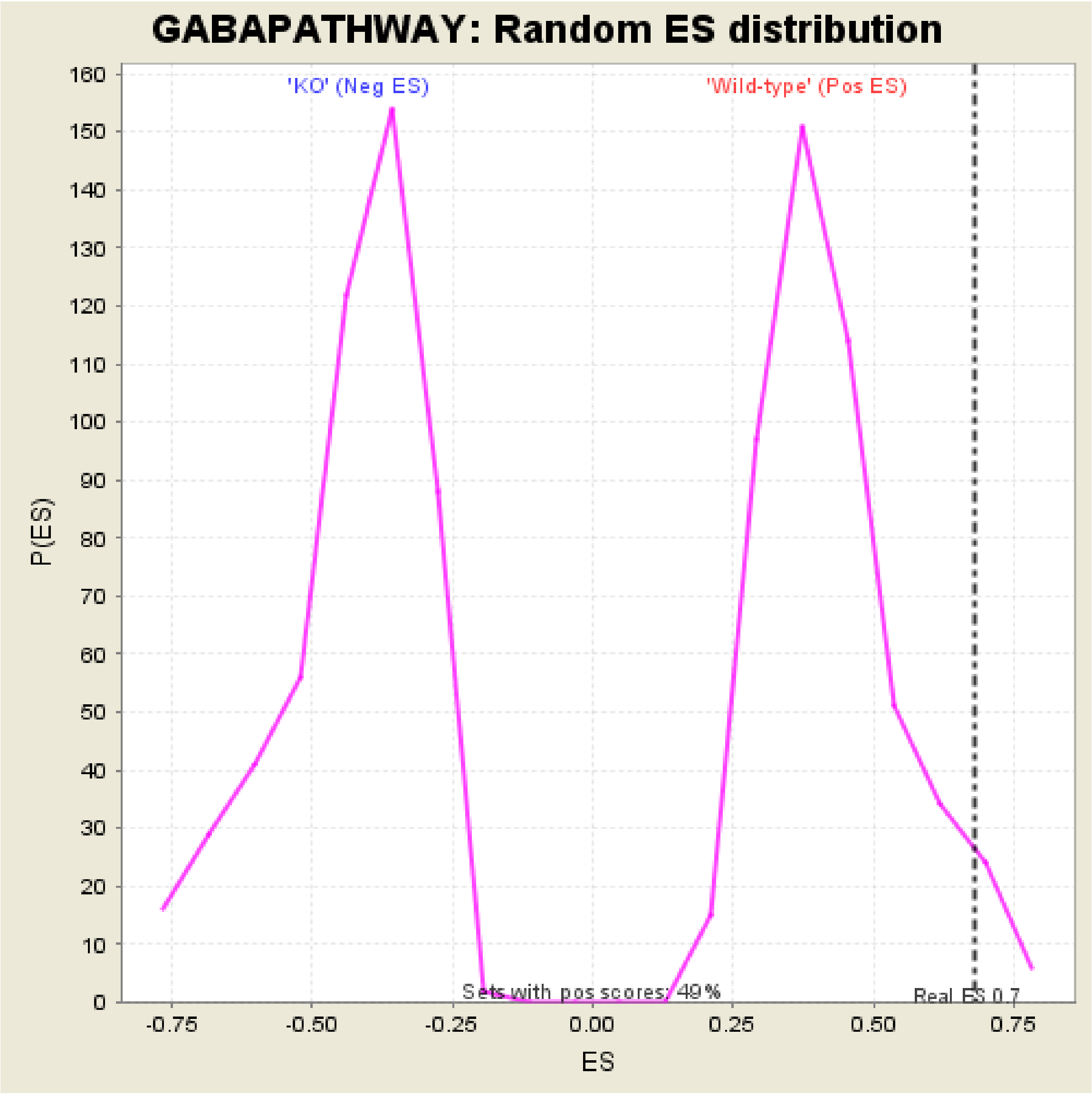

Fig 3: GABAPATHWAY: Random ES distribution
Gene set null distribution of ES for GABAPATHWAY

Table: GSEA Results Summary

Dataset	wt vs ko gsea_collapsed_to_symbols.wt vs ko cls file.cls#Wild-type_versus_KO
Phenotype	wt vs ko cls file.cls#Wild-type_versus_KO
Upregulated in class	Wild-type
GeneSet	AGPCRPATHWAY
Enrichment Score (ES)	0.6687819
Normalized Enrichment Score (NES)	1.4474283
Nominal p-value	0.019067796
FDR q-value	0.24359956
FWER p-Value	0.379

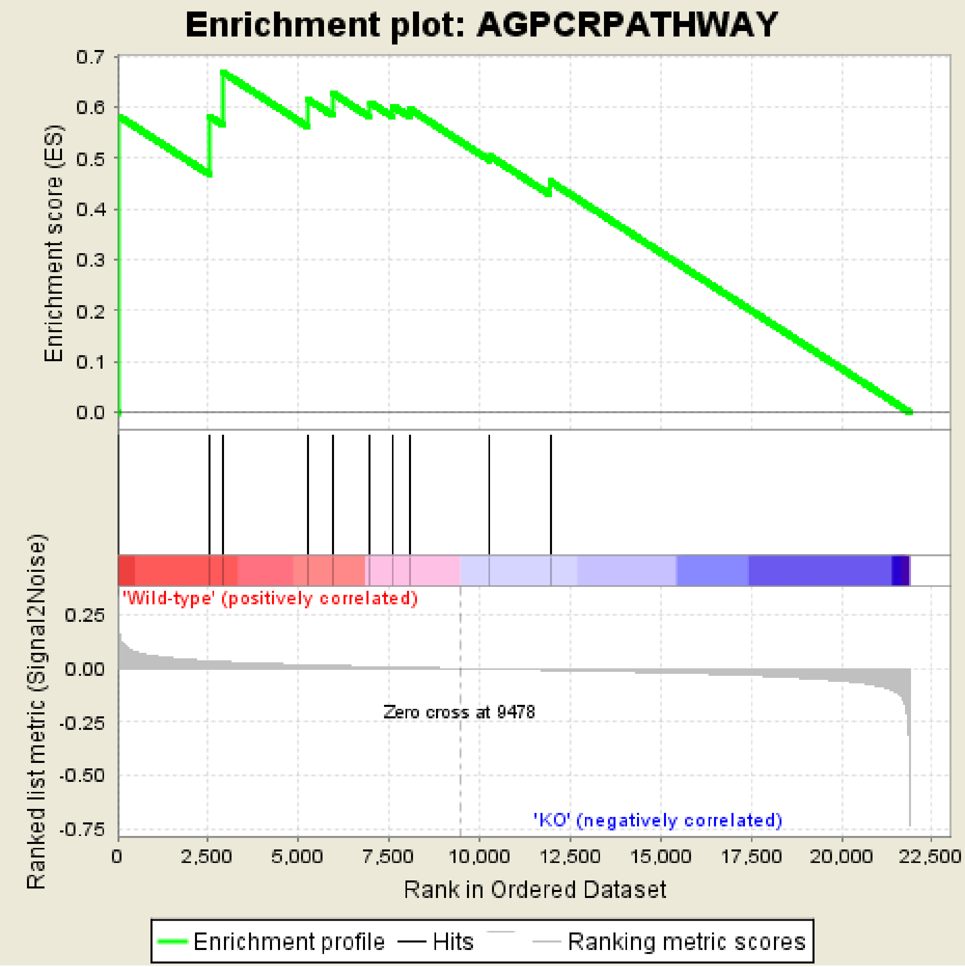

Fig 1: Enrichment plot: AGPCRPATHWAY
Profile of the Running ES Score & Positions of GeneSet Members on the Rank Ordered List

Table: GSEA details [plain text format]

	PROBE	GENE SYMBOL	GENE_TITLE	RANK IN GENE LIST	RANK METRIC SCORE	RUNNING ES	CORE ENRICHMENT
1	GNB1	GNB1 Entrez, Source, GeneCards	guanine nucleotide binding protein (G protein), beta polypeptide 1	30	0.175	0.5824	Yes
2	PRKCA	PRKCA Entrez, Source, GeneCards	protein kinase C, alpha	2542	0.034	0.5821	Yes
3	GNGT1	GNGT1 Entrez, Source, GeneCards	guanine nucleotide binding protein (G protein), gamma transducing activity polypeptide 1	2908	0.031	0.6688	Yes
4	PRKCB1	PRKCB1 Entrez, Source, GeneCards	protein kinase C, beta 1	5268	0.016	0.6149	No
5	PRKAR2A	PRKAR2A Entrez, Source, GeneCards	protein kinase, cAMP-dependent, regulatory, type II, alpha	5932	0.013	0.6279	No
6	GNAS	GNAS Entrez, Source, GeneCards	GNAS complex locus	6980	0.009	0.6093	No
7	PRKAR1A	PRKAR1A Entrez, Source, GeneCards	protein kinase, cAMP-dependent, regulatory, type I, alpha (tissue specific extinguisher 1)	7609	0.006	0.6020	No
8	PRKACB	PRKACB Entrez, Source, GeneCards	protein kinase, cAMP-dependent, catalytic, beta	8091	0.005	0.5957	No
9	PRKAR1B	PRKAR1B Entrez, Source, GeneCards	protein kinase, cAMP-dependent, regulatory, type I, beta	10288	-0.003	0.5039	No
10	PRKAR2B	PRKAR2B Entrez, Source, GeneCards	protein kinase, cAMP-dependent, regulatory, type II, beta	11944	-0.008	0.4545	No

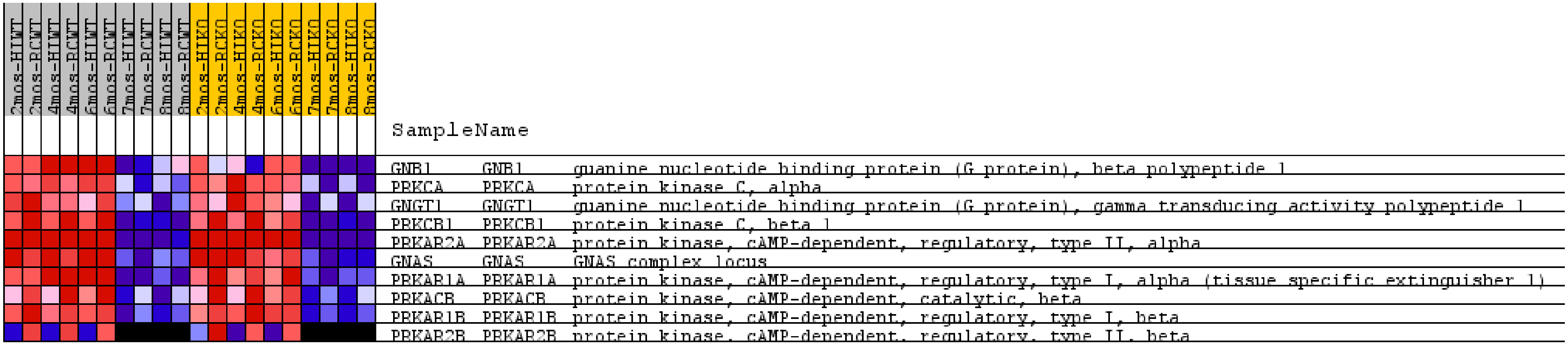

Fig 2: AGPCRPATHWAY
Blue-Pink O' Gram in the Space of the Analyzed GeneSet

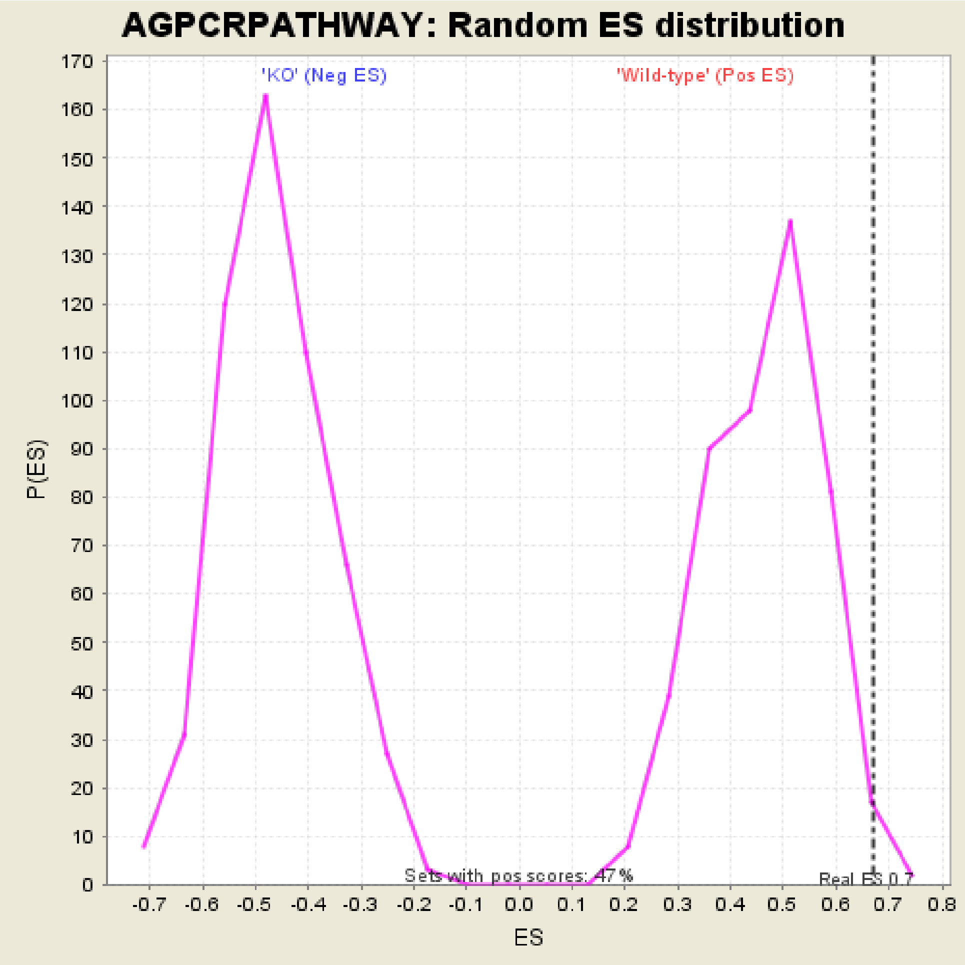

Fig 3: AGPCRPATHWAY: Random ES distribution
Gene set null distribution of ES for AGPCRPATHWAY

Table: GSEA Results Summary

Dataset	wt vs ko gsea_collapsed_to_symbols.wt vs ko cls file.cls#Wild-type_versus_KO
Phenotype	wt vs ko cls file.cls#Wild-type_versus_KO
Upregulated in class	KO
GeneSet	CTLA4PATHWAY
Enrichment Score (ES)	-0.5992027
Normalized Enrichment Score (NES)	-1.4970473
Nominal p-value	0.056112226
FDR q-value	0.23367235
FWER p-Value	0.304

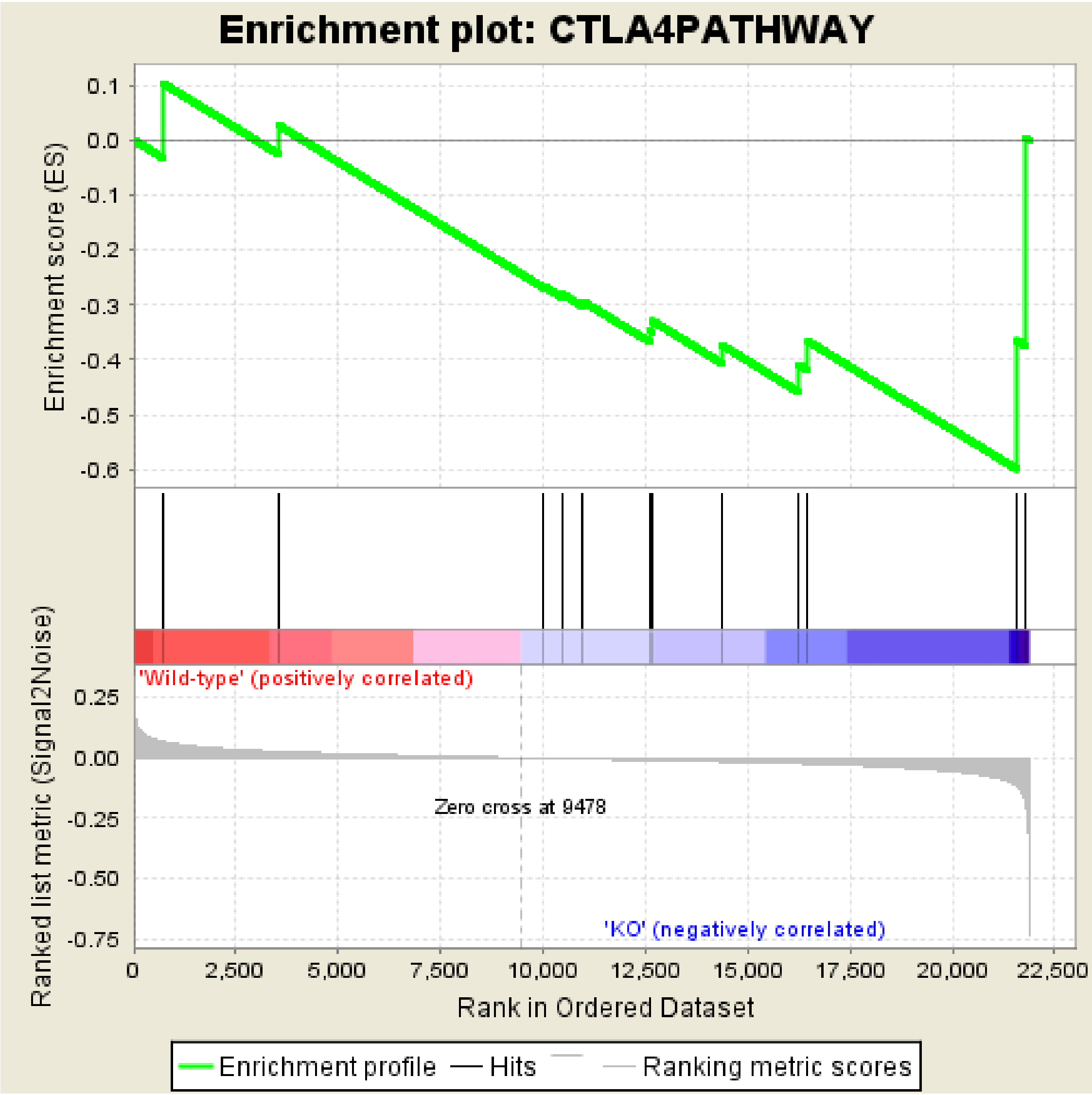

Fig 1: Enrichment plot: CTLA4PATHWAY
Profile of the Running ES Score & Positions of GeneSet Members on the Rank Ordered List

Table: GSEA details [plain text format]

	PROBE	GENE SYMBOL	GENE_TITLE	RANK IN GENE LIST	RANK METRIC SCORE	RUNNING ES	CORE ENRICHMENT

1	CTLA4	CTLA4 Entrez, Source, GeneCards	cytotoxic T-lymphocyte-associated protein 4	727	0.067	0.1027	No
2	PIK3CA	PIK3CA Entrez, Source, GeneCards	phosphoinositide-3-kinase, catalytic, alpha polypeptide	3535	0.026	0.0270	No
3	PTPN11	PTPN11 Entrez, Source, GeneCards	protein tyrosine phosphatase, non-receptor type 11 (Noonan syndrome 1)	10021	-0.002	-0.2660	No
4	ICOS	ICOS Entrez, Source, GeneCards	inducible T-cell co-stimulator	10454	-0.003	-0.2794	No
5	CD3E	CD3E Entrez, Source, GeneCards	CD3e molecule, epsilon (CD3-TCR complex)	10980	-0.005	-0.2937	No
6	PIK3R1	PIK3R1 Entrez, Source, GeneCards	phosphoinositide-3-kinase, regulatory subunit 1 (p85 alpha)	12611	-0.010	-0.3475	No
7	LCK	LCK Entrez, Source, GeneCards	lymphocyte-specific protein tyrosine kinase	12662	-0.010	-0.3287	No
8	GRB2	GRB2 Entrez, Source, GeneCards	growth factor receptor-bound protein 2	14356	-0.016	-0.3728	No
9	ITK	ITK Entrez, Source, GeneCards	IL2-inducible T-cell kinase	16254	-0.025	-0.4092	Yes
10	CD3D	CD3D Entrez, Source, GeneCards	CD3d molecule, delta (CD3-TCR complex)	16474	-0.026	-0.3663	Yes
11	CD3G	CD3G Entrez, Source, GeneCards	CD3g molecule, gamma (CD3-TCR complex)	21570	-0.116	-0.3645	Yes
12	CD86	CD86 Entrez, Source, GeneCards	CD86 molecule	21789	-0.187	0.0046	Yes

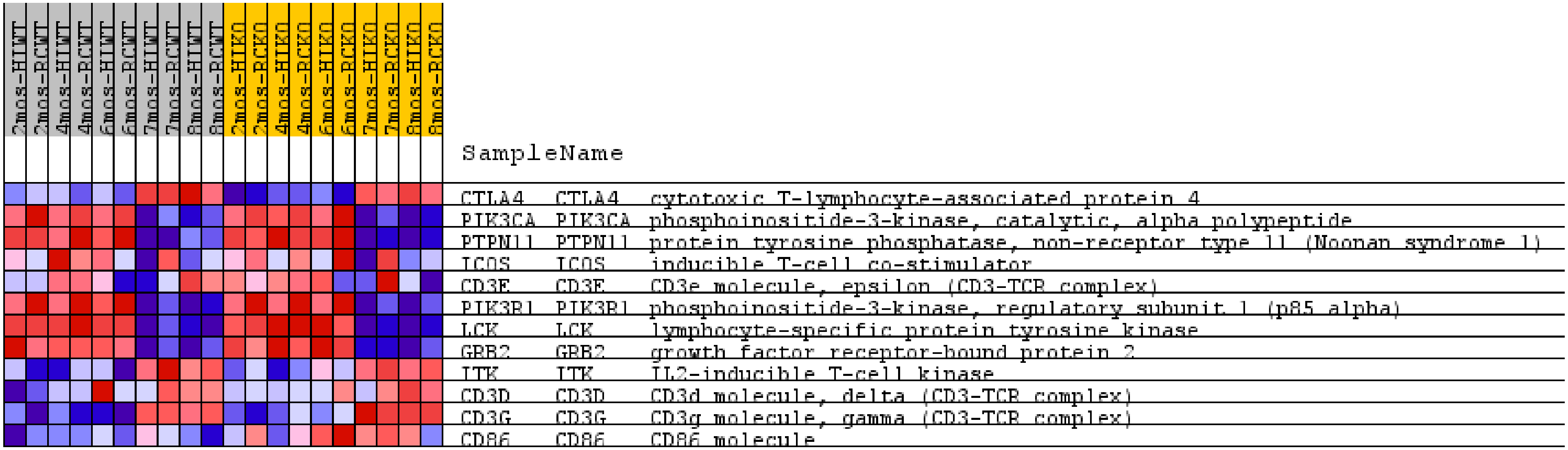

Fig 2: CTLA4PATHWAY
Blue-Pink O' Gram in the Space of the Analyzed GeneSet

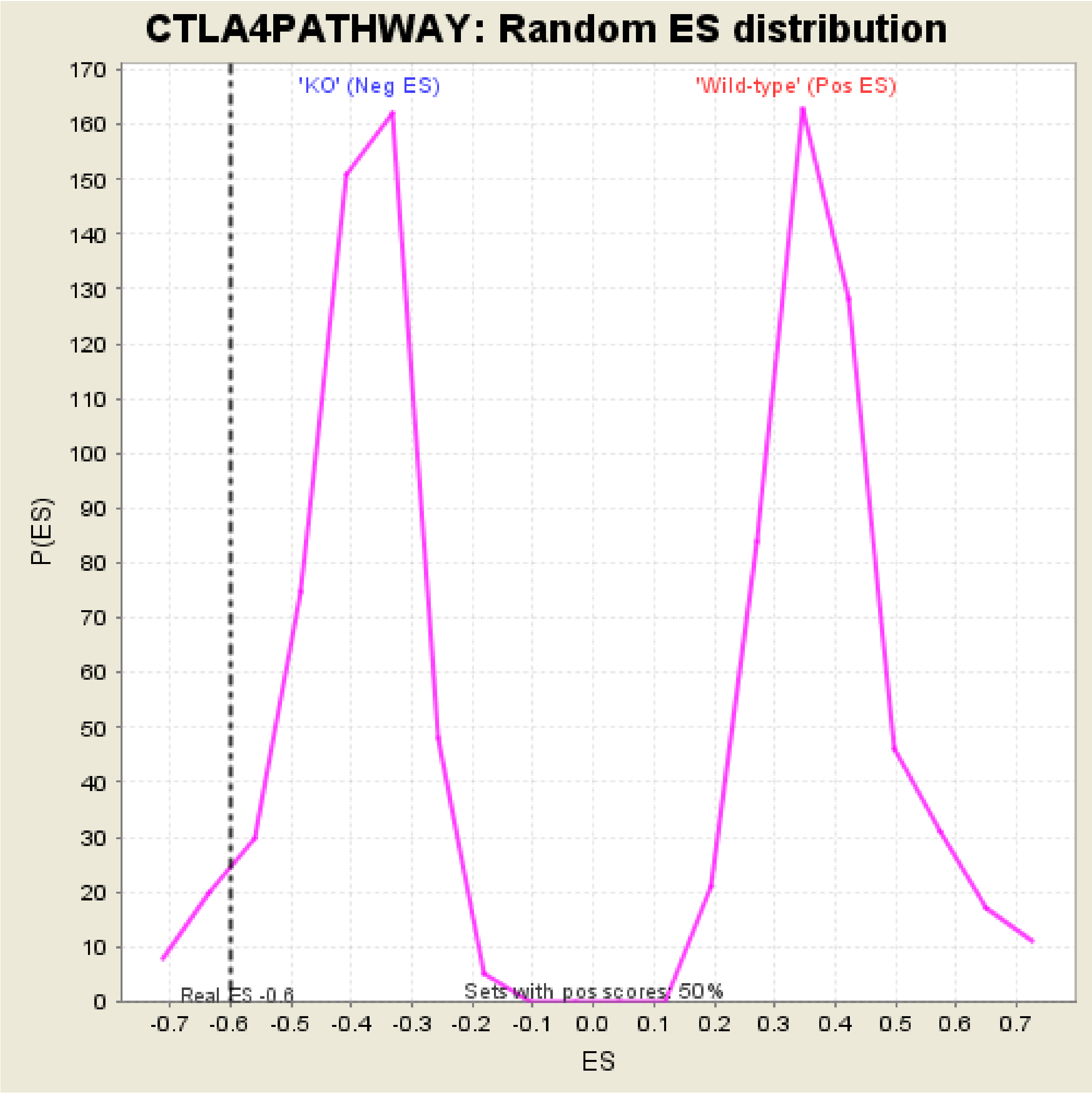

Fig 3: CTLA4PATHWAY: Random ES distribution
Gene set null distribution of ES for CTLA4PATHWAY

Table: GSEA Results Summary

Dataset	wt vs ko gsea_collapsed_to_symbols.wt vs ko cls file.cls#Wild-type_versus_KO
Phenotype	wt vs ko cls file.cls#Wild-type_versus_KO
Upregulated in class	KO
GeneSet	EXTRINSICPATHWAY
Enrichment Score (ES)	-0.7107476
Normalized Enrichment Score (NES)	-1.6888533
Nominal p-value	0.014613778
FDR q-value	0.08240235
FWER p-Value	0.097

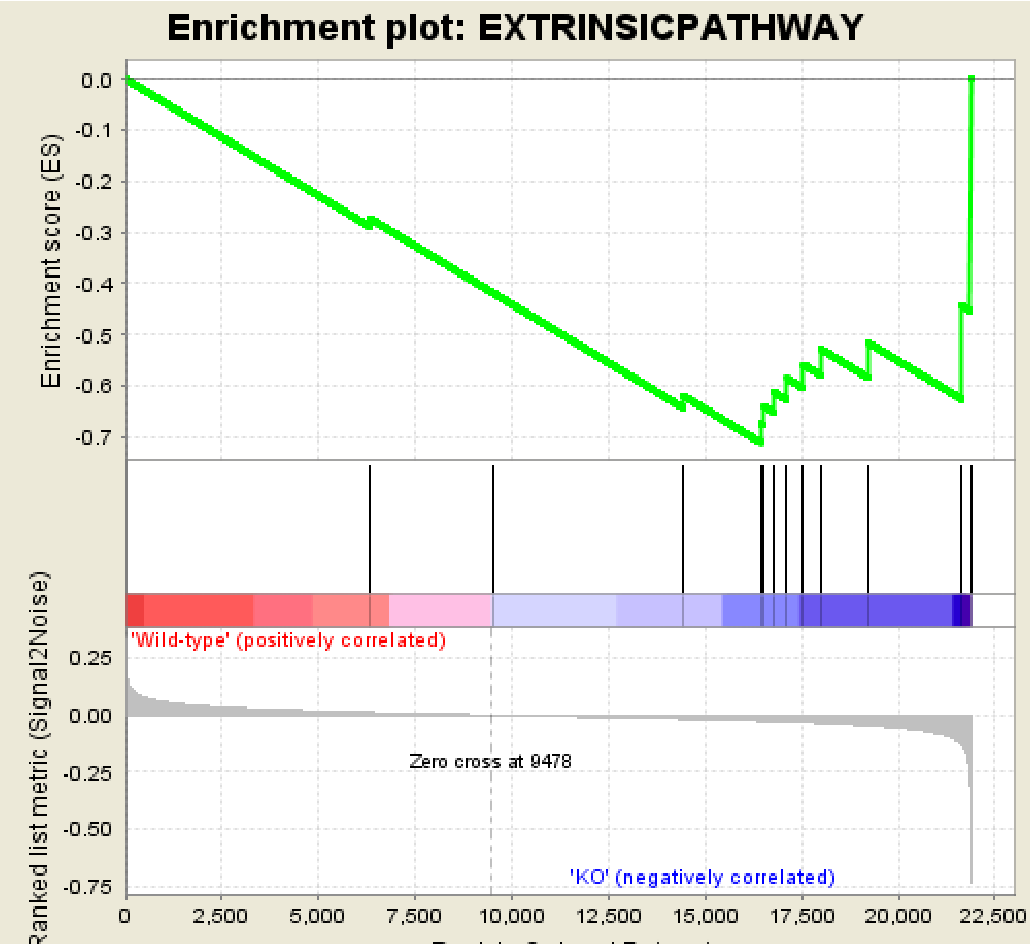

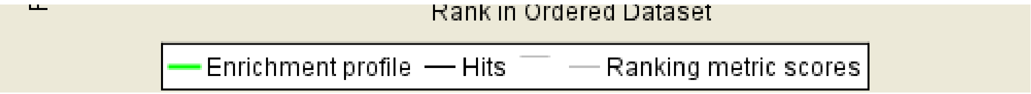

Fig 1: Enrichment plot: EXTRINSICPATHWAY
Profile of the Running ES Score & Positions of GeneSet Members on the Rank Ordered List

Table: GSEA details [plain text format]

	PROBE	GENE SYMBOL	GENE_TITLE	RANK IN GENE LIST	RANK METRIC SCORE	RUNNING ES	CORE ENRICHMENT
1	F3	F3 Entrez, Source, GeneCards	coagulation factor III (thromboplastin, tissue factor)	6324	0.011	-0.2728	No
2	TFPI	TFPI Entrez, Source, GeneCards	tissue factor pathway inhibitor (lipoprotein-associated coagulation inhibitor)	9514	-0.000	-0.4184	No
3	F2	F2 Entrez, Source, GeneCards	coagulation factor II (thrombin)	14434	-0.017	-0.6194	No
4	SERPINC1	SERPINC1 Entrez, Source, GeneCards	serpin peptidase inhibitor, clade C (antithrombin), member 1	16434	-0.026	-0.6739	Yes
5	FGA	FGA Entrez, Source, GeneCards	fibrinogen alpha chain	16525	-0.026	-0.6404	Yes
6	F2R	F2R Entrez, Source, GeneCards	coagulation factor II (thrombin) receptor	16759	-0.028	-0.6117	Yes
7	F7	F7 Entrez, Source, GeneCards	coagulation factor VII (serum prothrombin conversion accelerator)	17115	-0.030	-0.5854	Yes
8	FGG	FGG Entrez, Source, GeneCards	fibrinogen gamma chain	17540	-0.032	-0.5584	Yes
9	PROC	PROC Entrez, Source, GeneCards	protein C (inactivator of coagulation factors Va and VIIIa)	17997	-0.036	-0.5281	Yes
10	FGB	FGB Entrez, Source, GeneCards	fibrinogen beta chain	19231	-0.048	-0.5163	Yes
11	PROS1	PROS1 Entrez, Source, GeneCards	protein S (alpha)	21641	-0.128	-0.4434	Yes
12	F5	F5 Entrez, Source, GeneCards	coagulation factor V (proaccelerin, labile factor)	21865	-0.318	0.0011	Yes

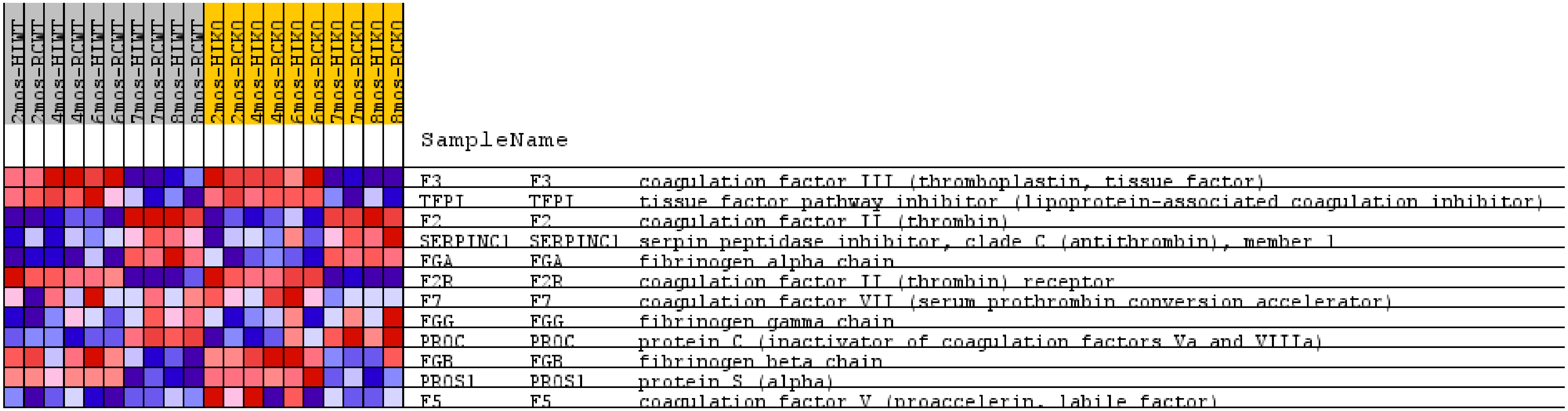

Fig 2: EXTRINSICPATHWAY
Blue-Pink O' Gram in the Space of the Analyzed GeneSet

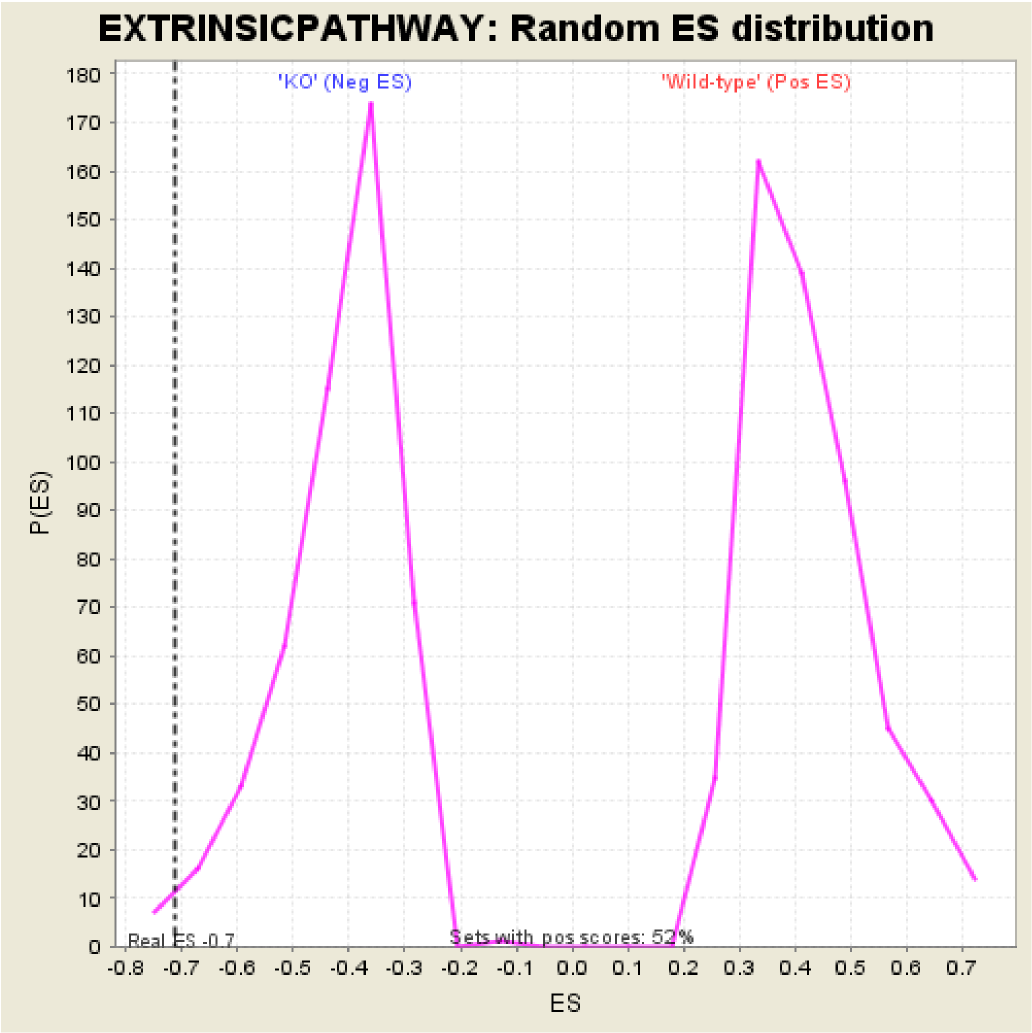

Fig 3: EXTRINSICPATHWAY: Random ES distribution
Gene set null distribution of ES for EXTRINSICPATHWAY

Table: GSEA Results Summary

Dataset	wt vs ko gsea_collapsed_to_symbols.wt vs ko cls file.cls#Wild-type_versus_KO
Phenotype	wt vs ko cls file.cls#Wild-type_versus_KO
Upregulated in class	KO
GeneSet	ACE2PATHWAY
Enrichment Score (ES)	-0.5622675
Normalized Enrichment Score (NES)	-1.428982
Nominal p-value	0.09670782
FDR q-value	0.1712118
FWER p-Value	0.443

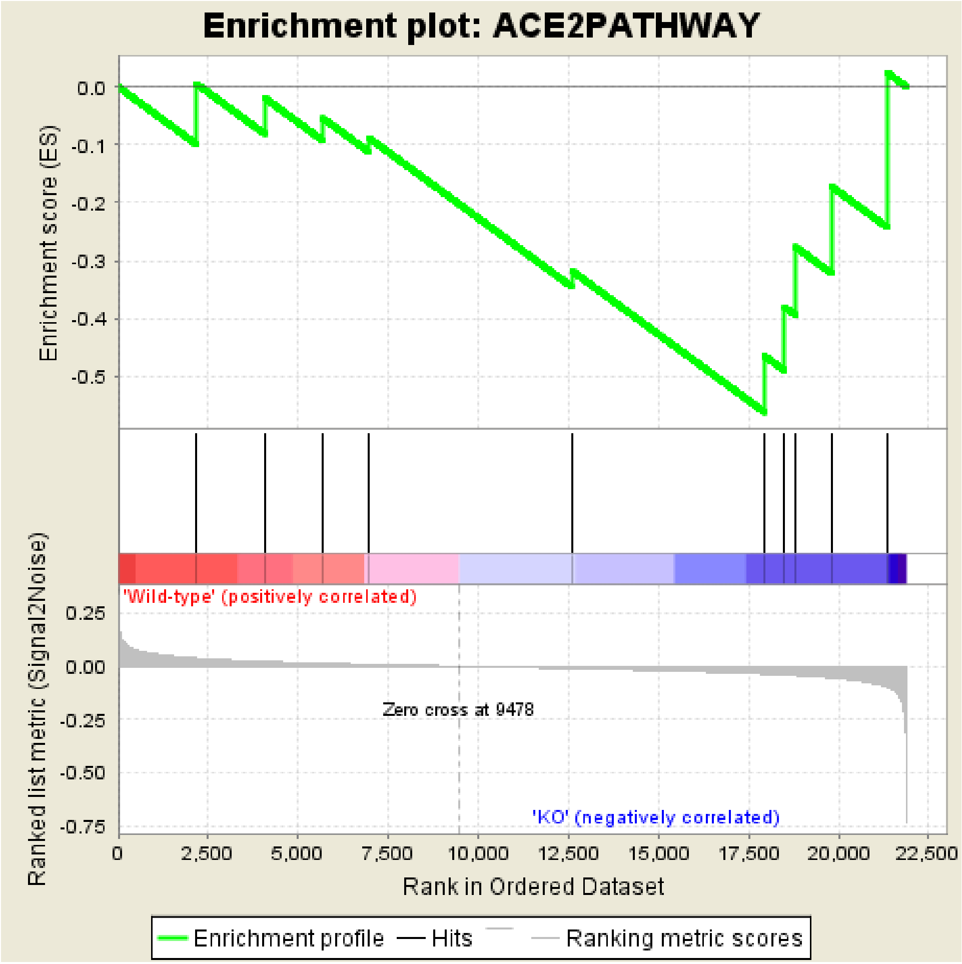

Fig 1: Enrichment plot: ACE2PATHWAY
Profile of the Running ES Score & Positions of GeneSet Members on the Rank Ordered List

Table: GSEA details ***[plain text format]***

	PROBE	GENE SYMBOL	GENE_TITLE	RANK IN GENE LIST	RANK METRIC SCORE	RUNNING ES	CORE ENRICHMENT
1	AGTR2	AGTR2 Entrez, Source, GeneCards	angiotensin II receptor, type 2	2165	0.038	0.0057	No

2	COL4A5	COL4A5 Entrez, Source, GeneCards	collagen, type IV, alpha 5 (Alport syndrome)	4055	0.023	-0.0184	No
3	COL4A6	COL4A6 Entrez, Source, GeneCards	collagen, type IV, alpha 6	5678	0.014	-0.0538	No
4	AGT	AGT Entrez, Source, GeneCards	angiotensinogen (serpin peptidase inhibitor, clade A, member 8)	6942	0.009	-0.0872	No
5	AGTR1	AGTR1 Entrez, Source, GeneCards	angiotensin II receptor, type 1	12588	-0.010	-0.3174	No
6	CMA1	CMA1 Entrez, Source, GeneCards	chymase 1, mast cell	17947	-0.035	-0.4650	Yes
7	COL4A3	COL4A3 Entrez, Source, GeneCards	collagen, type IV, alpha 3 (Goodpasture antigen)	18500	-0.040	-0.3803	Yes
8	COL4A1	COL4A1 Entrez, Source, GeneCards	collagen, type IV, alpha 1	18805	-0.043	-0.2764	Yes
9	COL4A2	COL4A2 Entrez, Source, GeneCards	collagen, type IV, alpha 2	19806	-0.055	-0.1720	Yes
10	COL4A4	COL4A4 Entrez, Source, GeneCards	collagen, type IV, alpha 4	21360	-0.097	0.0242	Yes

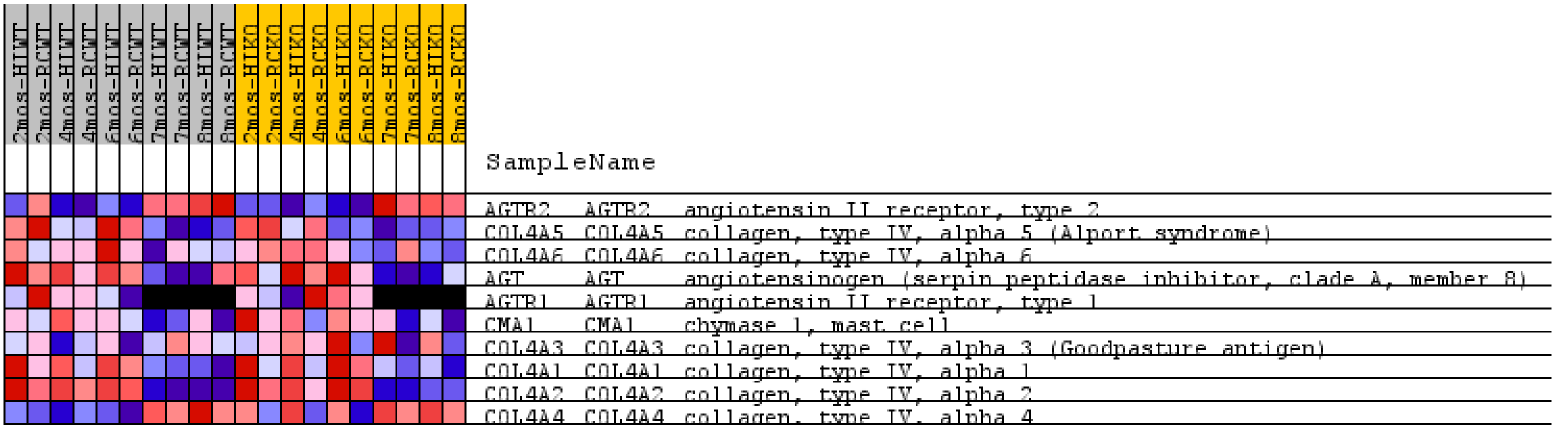

Fig 2: ACE2PATHWAY
Blue-Pink O' Gram in the Space of the Analyzed GeneSet

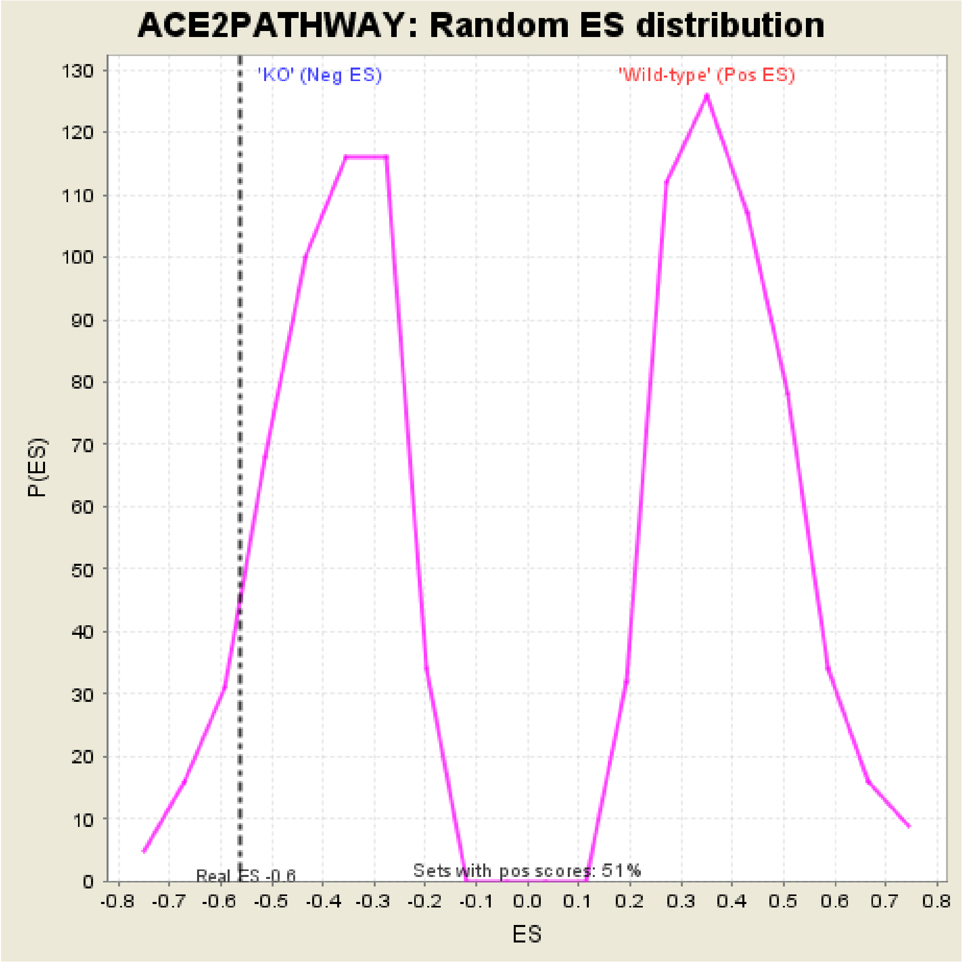

Fig 3: ACE2PATHWAY: Random ES distribution
Gene set null distribution of ES for ACE2PATHWAY

Table: GSEA Results Summary

Dataset	wt vs ko gsea_collapsed_to_symbols.wt vs ko cls file.cls#Wild-type_versus_KO
Phenotype	wt vs ko cls file.cls#Wild-type_versus_KO
Upregulated in class	KO
GeneSet	INTRINSICPATHWAY
Enrichment Score (ES)	-0.5796355
Normalized Enrichment Score (NES)	-1.4494264
Nominal p-value	0.02053388
FDR q-value	0.17798343
FWER p-Value	0.39

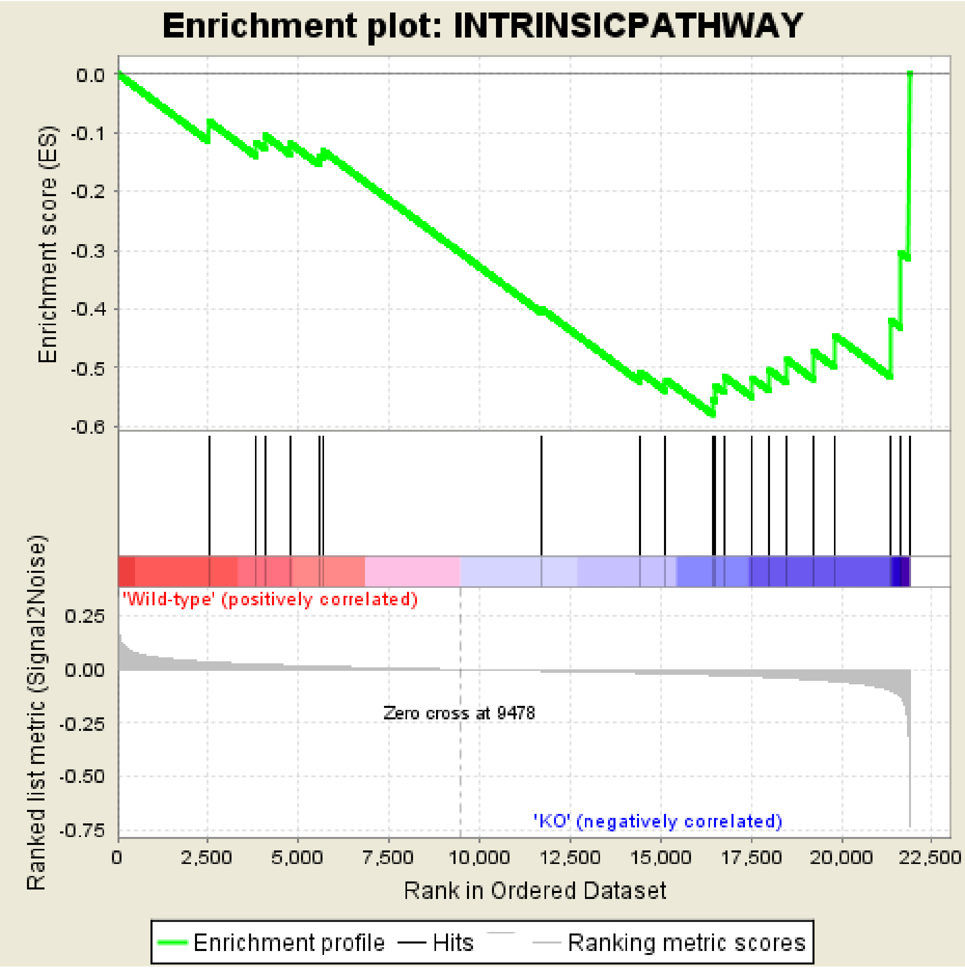

Fig 1: Enrichment plot: INTRINSICPATHWAY
Profile of the Running ES Score & Positions of GeneSet Members on the Rank Ordered List

Table: GSEA details [plain text format]

	PROBE	GENE SYMBOL	GENE_TITLE	RANK IN GENE LIST	RANK METRIC SCORE	RUNNING ES	CORE ENRICHMENT
1	KLKB1	KLKB1<br>Entrez, Source, GeneCards	kallikrein B, plasma (Fletcher factor) 1	2502	0.035	-0.0798	No
2	F11	F11<br>Entrez, Source, GeneCards	coagulation factor XI (plasma thromboplastin antecedent)	3816	0.024	-0.1159	No
3	COL4A5	COL4A5<br>Entrez, Source, GeneCards	collagen, type IV, alpha 5 (Alport syndrome)	4055	0.023	-0.1043	No
4	F9	F9<br>Entrez, Source, GeneCards	coagulation factor IX (plasma thromboplastic component, Christmas disease, hemophilia B)	4758	0.019	-0.1178	No
5	F8	F8<br>Entrez, Source, GeneCards	coagulation factor VIII, procoagulant component (hemophilia A)	5574	0.015	-0.1406	No
6	COL4A6	COL4A6<br>Entrez, Source, GeneCards	collagen, type IV, alpha 6	5678	0.014	-0.1313	No
7	F10	F10<br>Entrez, Source, GeneCards	coagulation factor X	11700	-0.007	-0.3995	No
8	F2	F2<br>Entrez, Source, GeneCards	coagulation factor II (thrombin)	14434	-0.017	-0.5079	No
9	F12	F12<br>Entrez, Source, GeneCards	coagulation factor XII (Hageman factor)	15142	-0.020	-0.5206	No
10	SERPINC1	SERPINC1<br>Entrez, Source, GeneCards	serpin peptidase inhibitor, clade C (antithrombin), member 1	16434	-0.026	-0.5540	Yes
11	FGA	FGA<br>Entrez, Source, GeneCards	fibrinogen alpha chain	16525	-0.026	-0.5319	Yes
12	F2R	F2R<br>Entrez, Source, GeneCards	coagulation factor II (thrombin) receptor	16759	-0.028	-0.5152	Yes
13	FGG	FGG<br>Entrez, Source, GeneCards	fibrinogen gamma chain	17540	-0.032	-0.5186	Yes
14	PROC	PROC<br>Entrez, Source, GeneCards	protein C (inactivator of coagulation factors Va and VIIIa)	17997	-0.036	-0.5039	Yes
15	COL4A3	COL4A3<br>Entrez, Source, GeneCards	collagen, type IV, alpha 3 (Goodpasture antigen)	18500	-0.040	-0.4871	Yes
16	FGB	FGB<br>Entrez, Source, GeneCards	fibrinogen beta chain	19231	-0.048	-0.4731	Yes

17	COL4A2	COL4A2 Entrez, Source, GeneCards	collagen, type IV, alpha 2	19806	-0.055	-0.4450	Yes
18	COL4A4	COL4A4 Entrez, Source, GeneCards	collagen, type IV, alpha 4	21360	-0.097	-0.4194	Yes
19	PROS1	PROS1 Entrez, Source, GeneCards	protein S (alpha)	21641	-0.128	-0.3049	Yes
20	F5	F5 Entrez, Source, GeneCards	coagulation factor V (proaccelerin, labile factor)	21865	-0.318	0.0011	Yes

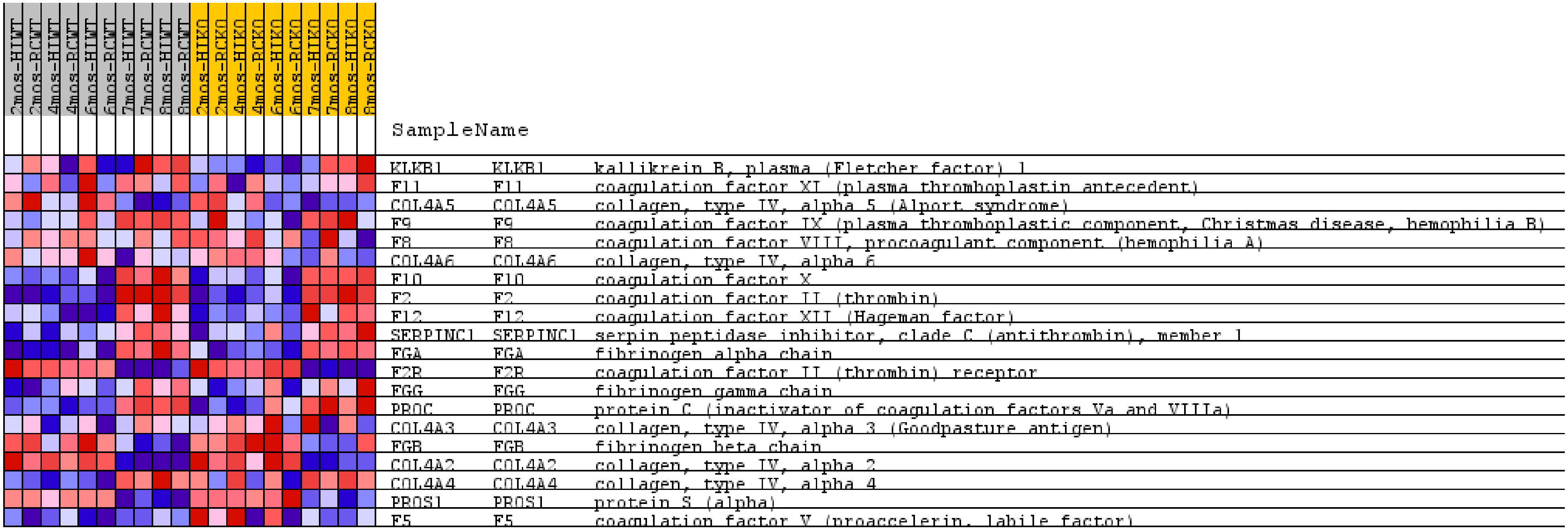

Fig 2: INTRINSICPATHWAY
Blue-Pink O' Gram in the Space of the Analyzed GeneSet

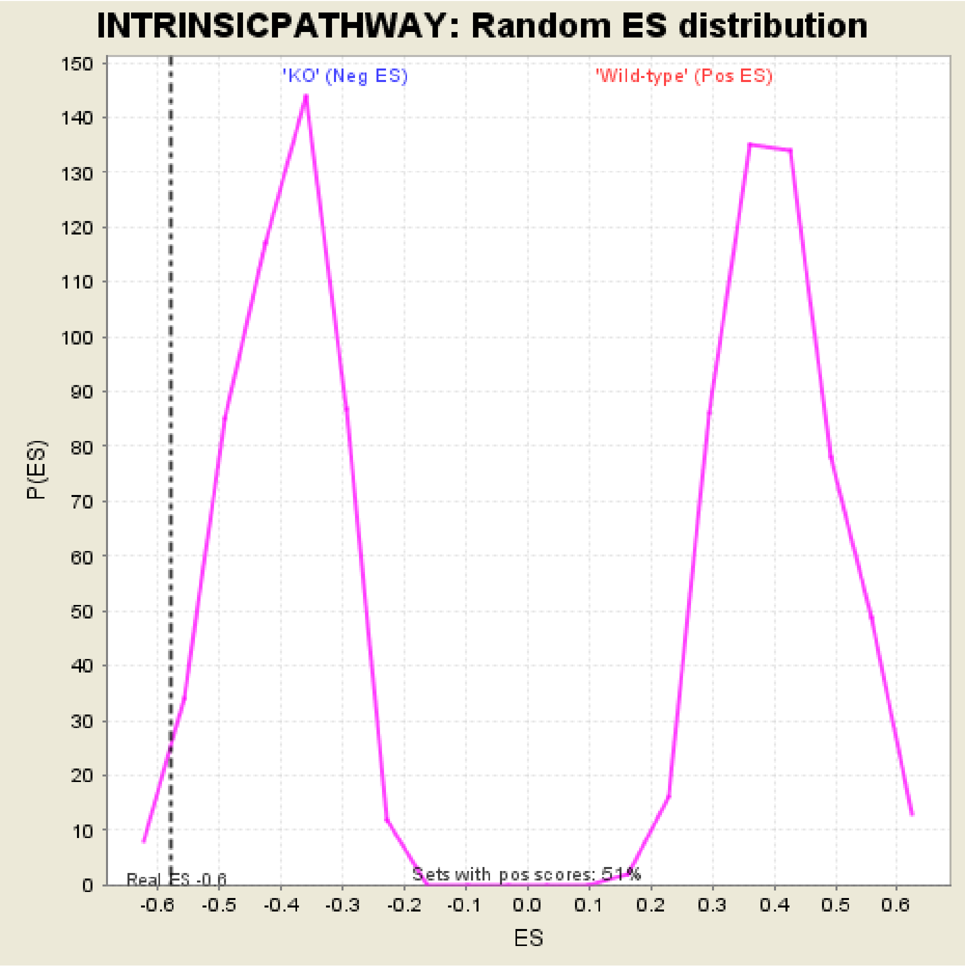

Fig 3: INTRINSICPATHWAY: Random ES distribution
Gene set null distribution of ES for INTRINSICPATHWAY

Table: GSEA Results Summary

Dataset	wt vs ko gsea_collapsed_to_symbols.wt vs ko cls file.cls#Wild-type_versus_KO
Phenotype	wt vs ko cls file.cls#Wild-type_versus_KO
Upregulated in class	KO
GeneSet	LAIRPATHWAY
Enrichment Score (ES)	-0.6730978
Normalized Enrichment Score (NES)	-1.4520286
Nominal p-value	0.046184737
FDR q-value	0.19823746
FWER p-Value	0.38

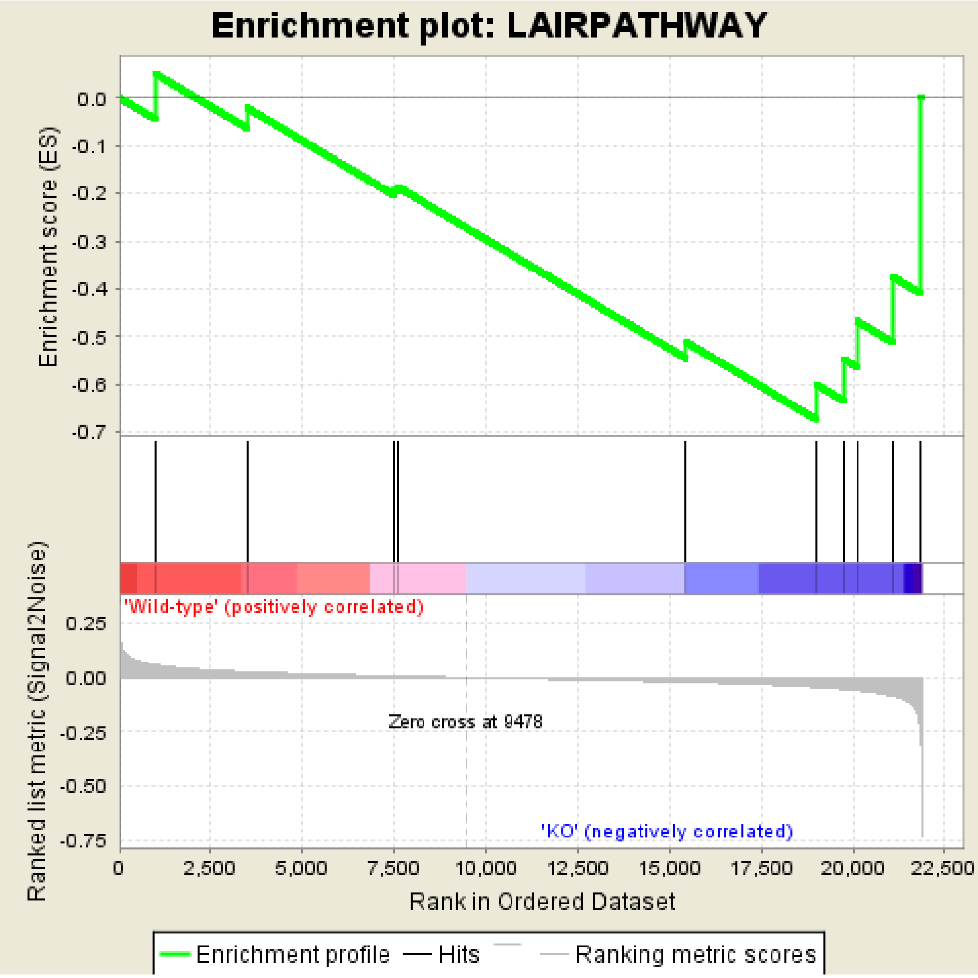

Fig 1: Enrichment plot: LAIRPATHWAY
Profile of the Running ES Score & Positions of GeneSet Members on the Rank Ordered List

Table: GSEA details ***[plain text format]***

	PROBE	GENE SYMBOL	GENE_TITLE	RANK IN GENE LIST	RANK METRIC SCORE	RUNNING ES	CORE ENRICHMENT
1	ITGA4	ITGA4 Entrez, Source, GeneCards	integrin, alpha 4 (antigen CD49D, alpha 4 subunit of VLA-4 receptor)	979	0.059	0.0509	No
2	IL1A	IL1A Entrez, Source, GeneCards	interleukin 1, alpha	3477	0.026	-0.0203	No
3	SELP	SELP Entrez, Source, GeneCards	selectin P (granule membrane protein 140kDa, antigen CD62)	7501	0.007	-0.1931	No
4	ITGB1	ITGB1 Entrez, Source, GeneCards	integrin, beta 1 (fibronectin receptor, beta polypeptide, antigen CD29 includes MDF2, MSK12)	7580	0.007	-0.1861	No
5	IL6	IL6 Entrez, Source, GeneCards	interleukin 6 (interferon, beta 2)	15462	-0.021	-0.5119	No
6	C6	C6 Entrez, Source, GeneCards	complement component 6	18990	-0.045	-0.6002	Yes
7	VCAM1	VCAM1 Entrez, Source, GeneCards	vascular cell adhesion molecule 1	19742	-0.054	-0.5473	Yes
8	TNF	TNF Entrez, Source, GeneCards	tumor necrosis factor (TNF superfamily, member 2)	20111	-0.059	-0.4676	Yes
9	ICAM1	ICAM1 Entrez, Source, GeneCards	intercellular adhesion molecule 1 (CD54), human rhinovirus receptor	21079	-0.084	-0.3748	Yes
10	ITGB2	ITGB2 Entrez, Source, GeneCards	integrin, beta 2 (complement component 3 receptor 3 and 4 subunit)	21839	-0.253	0.0023	Yes

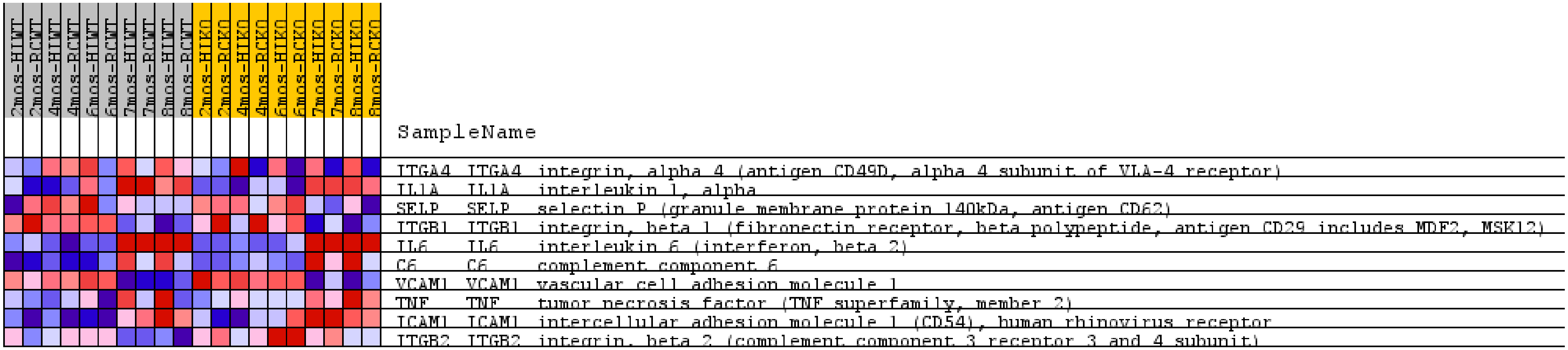

Fig 2: LAIRPATHWAY
Blue-Pink O' Gram in the Space of the Analyzed GeneSet

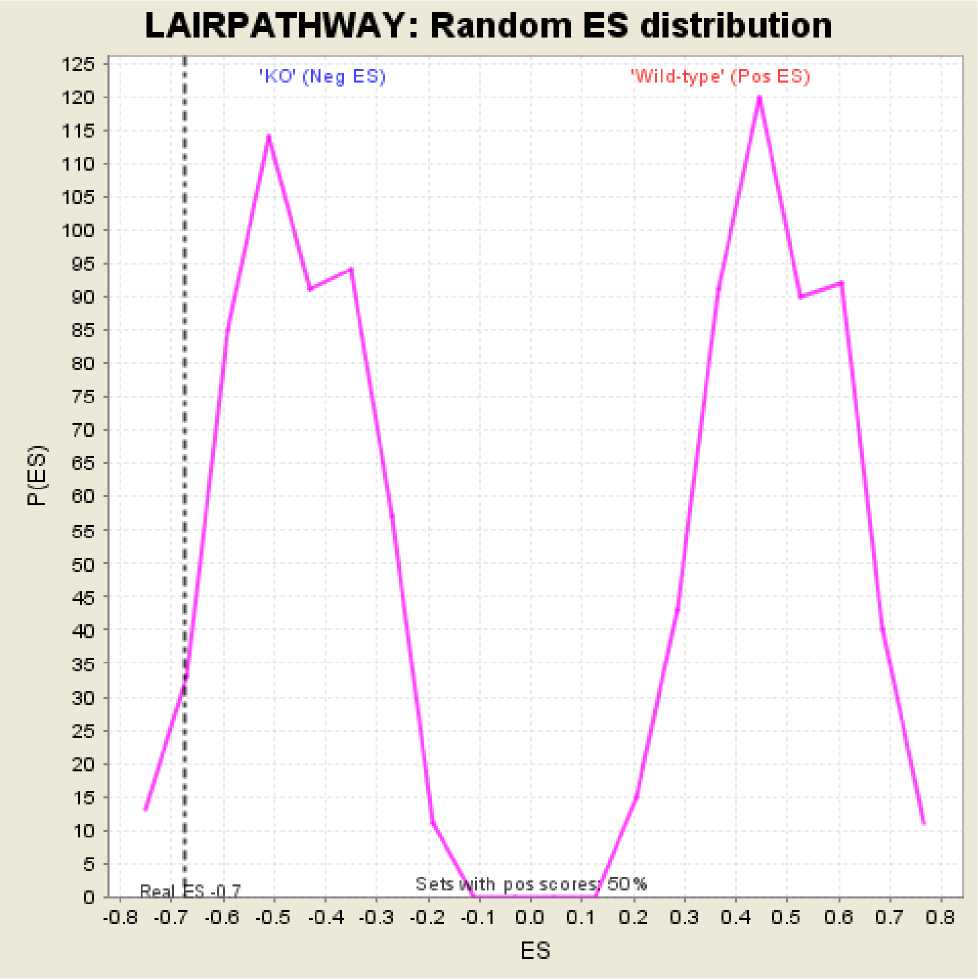

Fig 3: LAIRPATHWAY: Random ES distribution
Gene set null distribution of ES for LAIRPATHWAY

Table: GSEA Results Summary

Dataset	wt vs ko gsea_collapsed_to_symbols.wt vs ko cls file.cls#Wild-type_versus_KO
Phenotype	wt vs ko cls file.cls#Wild-type_versus_KO
Upregulated in class	KO
GeneSet	MCALPAINPATHWAY
Enrichment Score (ES)	-0.5250673
Normalized Enrichment Score (NES)	-1.4360536
Nominal p-value	0.0058479533
FDR q-value	0.17932285
FWER p-Value	0.426

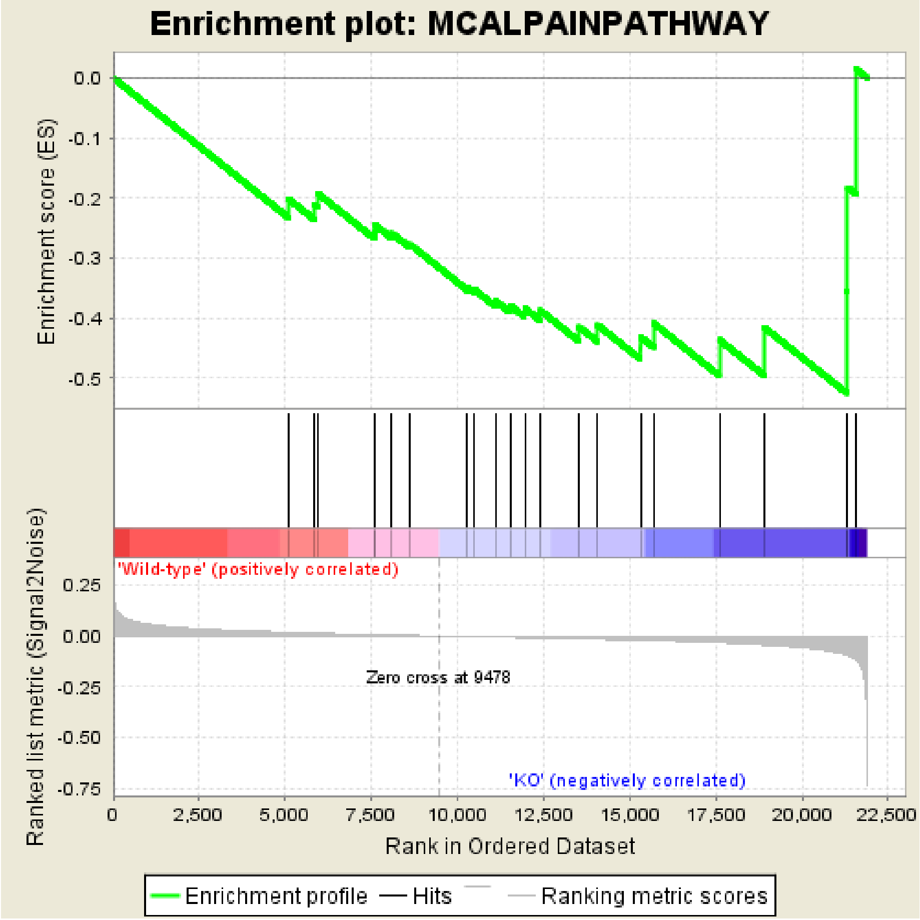

Fig 1: Enrichment plot: MCALPAINPATHWAY
Profile of the Running ES Score & Positions of GeneSet Members on the Rank Ordered List

Table: GSEA details [plain text format]

	PROBE	GENE SYMBOL	GENE_TITLE	RANK IN GENE LIST	RANK METRIC SCORE	RUNNING ES	CORE ENRICHMENT
1	EGF	EGF<br>Entrez, Source, GeneCards	epidermal growth factor (beta-urogastrone)	5091	0.017	-0.2019	No
2	PTK2	PTK2<br>Entrez, Source, GeneCards	PTK2 protein tyrosine kinase 2	5843	0.013	-0.2119	No
3	PRKAR2A	PRKAR2A<br>Entrez, Source, GeneCards	protein kinase, cAMP-dependent, regulatory, type II, alpha	5932	0.013	-0.1923	No
4	ITGB1	ITGB1<br>Entrez, Source, GeneCards	integrin, beta 1 (fibronectin receptor, beta polypeptide, antigen CD29 includes MDF2, MSK12)	7580	0.007	-0.2557	No
5	PRKAR1A	PRKAR1A<br>Entrez, Source, GeneCards	protein kinase, cAMP-dependent, regulatory, type I, alpha (tissue specific extinguisher 1)	7609	0.006	-0.2453	No
6	PRKACB	PRKACB<br>Entrez, Source, GeneCards	protein kinase, cAMP-dependent, catalytic, beta	8091	0.005	-0.2588	No
7	TLN1	TLN1<br>Entrez, Source, GeneCards	talin 1	8609	0.003	-0.2772	No
8	PRKAR1B	PRKAR1B<br>Entrez, Source, GeneCards	protein kinase, cAMP-dependent, regulatory, type I, beta	10288	-0.003	-0.3493	No
9	HRAS	HRAS<br>Entrez, Source, GeneCards	v-Ha-ras Harvey rat sarcoma viral oncogene homolog	10473	-0.003	-0.3519	No
10	CAPN2	CAPN2<br>Entrez, Source, GeneCards	calpain 2, (m/II) large subunit	11100	-0.005	-0.3712	No
11	MAPK3	MAPK3<br>Entrez, Source, GeneCards	mitogen-activated protein kinase 3	11521	-0.006	-0.3786	No
12	PRKAR2B	PRKAR2B<br>Entrez, Source, GeneCards	protein kinase, cAMP-dependent, regulatory, type II, beta	11944	-0.008	-0.3835	No
13	CAPN1	CAPN1<br>Entrez, Source, GeneCards	calpain 1, (mu/I) large subunit	12400	-0.009	-0.3873	No
14	MAPK1	MAPK1<br>Entrez, Source, GeneCards	mitogen-activated protein kinase 1	13530	-0.013	-0.4149	No
15	PXN	PXN<br>Entrez, Source, GeneCards	paxillin	14070	-0.015	-0.4118	No
16	CXCR3	CXCR3<br>Entrez, Source, GeneCards	chemokine (C-X-C motif) receptor 3	15304	-0.020	-0.4311	No
17	VIL2	VIL2<br>Entrez, Source, GeneCards	villin 2 (ezrin)	15688	-0.022	-0.4085	No
18	CAPNS1	CAPNS1<br>Entrez, Source, GeneCards	calpain, small subunit 1	17599	-0.033	-0.4361	Yes
19	EGFR	EGFR<br>Entrez, Source, GeneCards	epidermal growth factor receptor (erythroblastic leukemia viral (v-erb-b) oncogene homolog, avian)	18911	-0.044	-0.4160	Yes

20	MYL2	MYL2<br>Entrez, Source, GeneCards	myosin, light chain 2, regulatory, cardiac, slow	21297	-0.093	-0.3549	Yes
21	CAPNS2	CAPNS2<br>Entrez, Source, GeneCards	calpain, small subunit 2	21329	-0.095	-0.1827	Yes
22	MYLK	MYLK<br>Entrez, Source, GeneCards	myosin, light chain kinase	21563	-0.114	0.0150	Yes

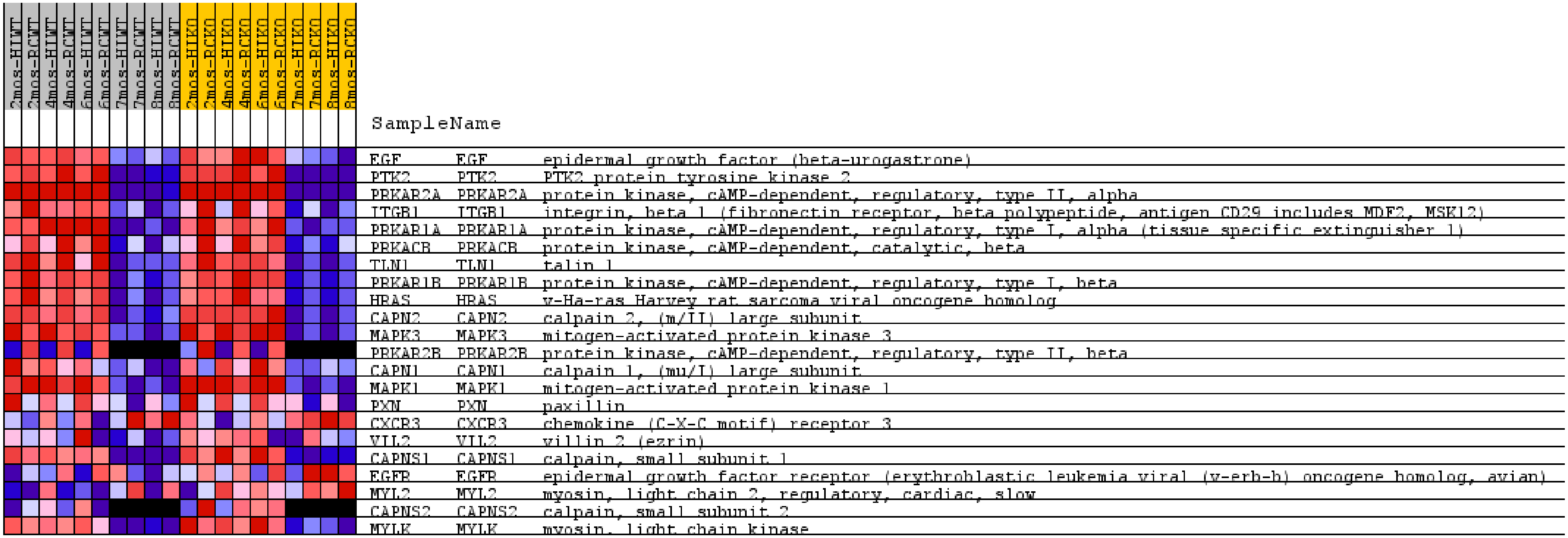

Fig 2: MCALPAINPATHWAY
Blue-Pink O' Gram in the Space of the Analyzed GeneSet

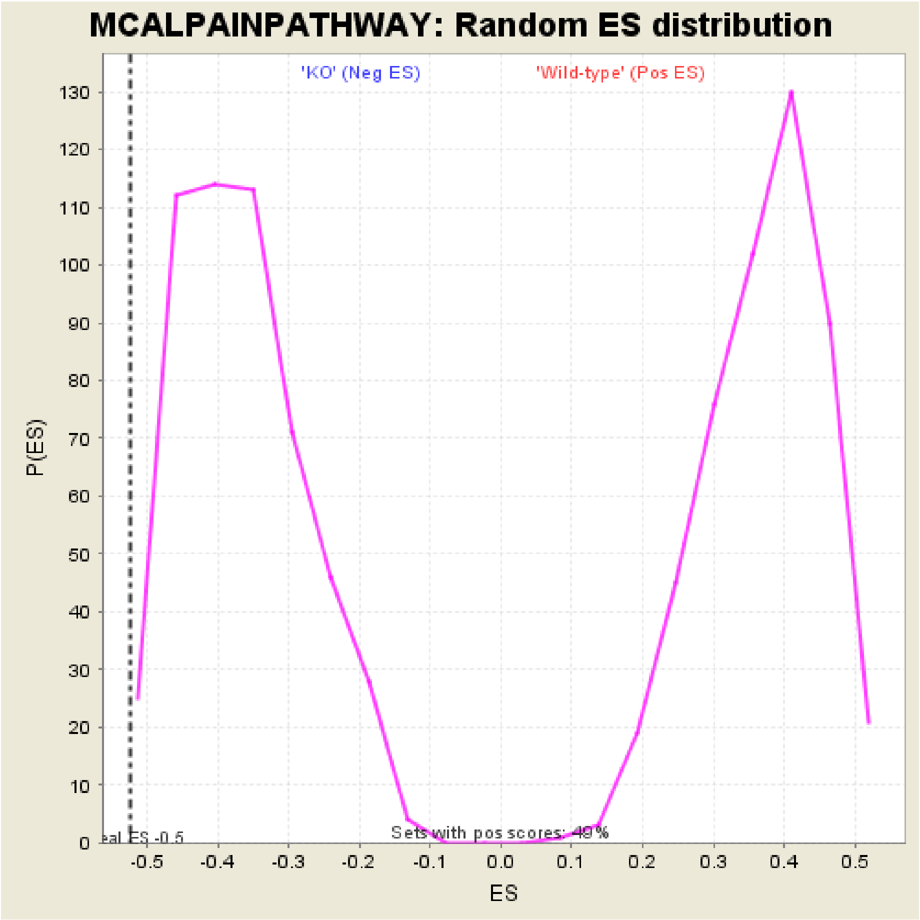

Fig 3: MCALPAINPATHWAY: Random ES distribution
Gene set null distribution of ES for MCALPAINPATHWAY

Table: GSEA Results Summary

Dataset	wt vs ko gsea_collapsed_to_symbols.wt vs ko cls file.cls#Wild-type_versus_KO
Phenotype	wt vs ko cls file.cls#Wild-type_versus_KO
Upregulated in class	KO
GeneSet	NKCELLSPATHWAY
Enrichment Score (ES)	-0.5025256
Normalized Enrichment Score (NES)	-1.703031
Nominal p-value	0.025490196
FDR q-value	0.13899715
FWER p-Value	0.084

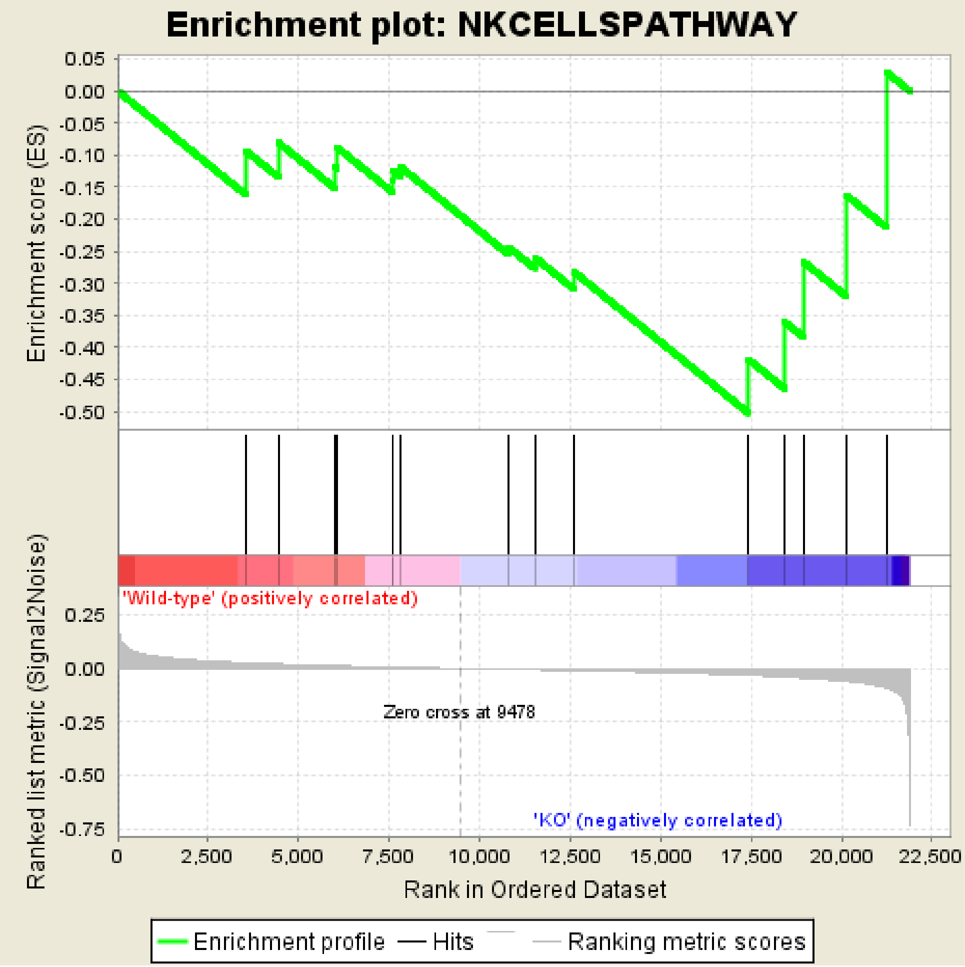

Fig 1: Enrichment plot: NKCELLSPATHWAY
Profile of the Running ES Score & Positions of GeneSet Members on the Rank Ordered List

Table: GSEA details [plain text format]

	PROBE	GENE SYMBOL	GENE_TITLE	RANK IN GENE LIST	RANK METRIC SCORE	RUNNING ES	CORE ENRICHMENT
1	PIK3CA	PIK3CA Entrez, Source, GeneCards	phosphoinositide-3-kinase, catalytic, alpha polypeptide	3535	0.026	-0.0931	No
2	LAT	LAT Entrez, Source, GeneCards	linker for activation of T cells	4434	0.020	-0.0801	No
3	KLRD1	KLRD1 Entrez, Source, GeneCards	killer cell lectin-like receptor subfamily D, member 1	6004	0.013	-0.1182	No
4	PAK1	PAK1 Entrez, Source, GeneCards	p21/Cdc42/Rac1-activated kinase 1 (STE20 homolog, yeast)	6055	0.012	-0.0876	No
5	ITGB1	ITGB1 Entrez, Source, GeneCards	integrin, beta 1 (fibronectin receptor, beta polypeptide, antigen CD29 includes MDF2, MSK12)	7580	0.007	-0.1400	No
6	RAC1	RAC1 Entrez, Source, GeneCards	ras-related C3 botulinum toxin substrate 1 (rho family, small GTP binding protein Rac1)	7597	0.006	-0.1236	No
7	MAP2K1	MAP2K1 Entrez, Source, GeneCards	mitogen-activated protein kinase kinase 1	7814	0.006	-0.1184	No
8	PTK2B	PTK2B Entrez, Source, GeneCards	PTK2B protein tyrosine kinase 2 beta	10795	-0.004	-0.2435	No
9	MAPK3	MAPK3 Entrez, Source, GeneCards	mitogen-activated protein kinase 3	11521	-0.006	-0.2595	No
10	PIK3R1	PIK3R1 Entrez, Source, GeneCards	phosphoinositide-3-kinase, regulatory subunit 1 (p85 alpha)	12611	-0.010	-0.2822	No
11	SYK	SYK Entrez, Source, GeneCards	spleen tyrosine kinase	17431	-0.032	-0.4187	Yes
12	IL18	IL18 Entrez, Source, GeneCards	interleukin 18 (interferon-gamma-inducing factor)	18439	-0.039	-0.3604	Yes
13	PTPN6	PTPN6 Entrez, Source, GeneCards	protein tyrosine phosphatase, non-receptor type 6	18947	-0.044	-0.2660	Yes
14	KLRC3	KLRC3 Entrez, Source, GeneCards	killer cell lectin-like receptor subfamily C, member 3	20135	-0.060	-0.1623	Yes
15	VAV1	VAV1 Entrez, Source, GeneCards	vav 1 oncogene	21249	-0.092	0.0293	Yes

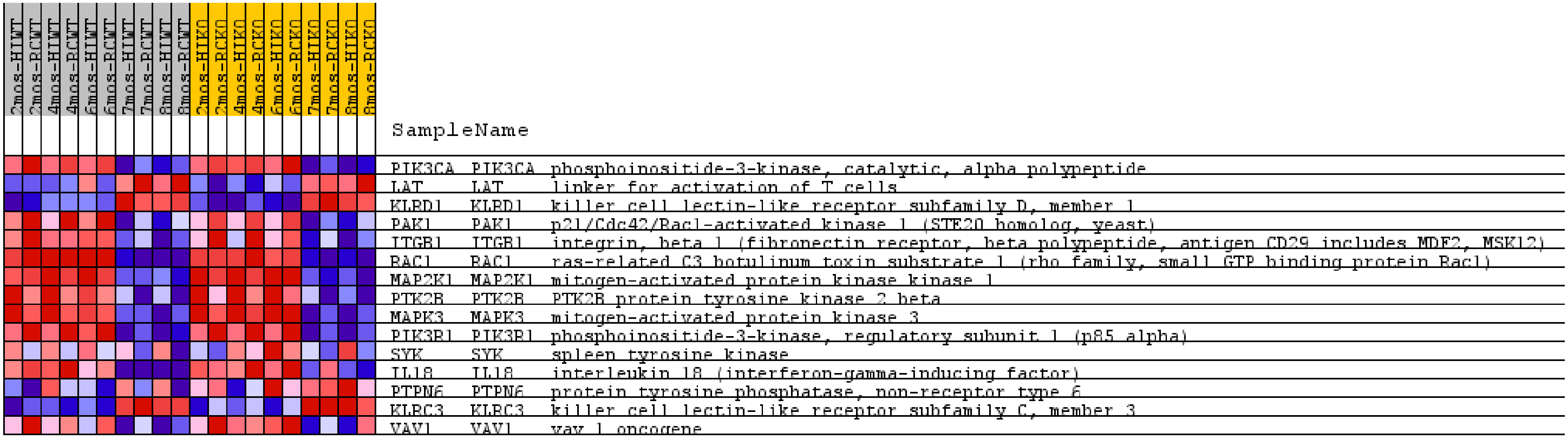

Fig 2: NKCELLSPATHWAY
Blue-Pink O' Gram in the Space of the Analyzed GeneSet

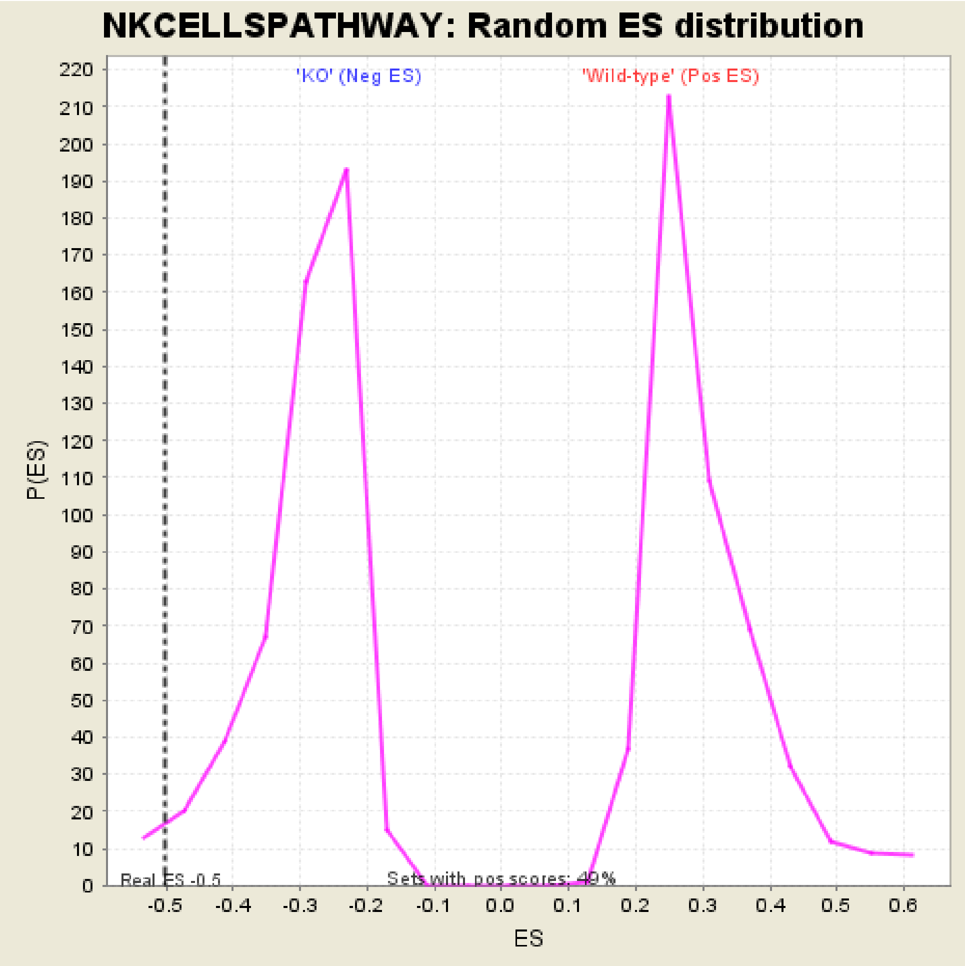

Fig 3: NKCELLSPATHWAY: Random ES distribution
Gene set null distribution of ES for NKCELLSPATHWAY

Table: GSEA Results Summary

Dataset	wt vs ko gsea_collapsed_to_symbols.wt vs ko cls file.cls#Wild-type_versus_KO
Phenotype	wt vs ko cls file.cls#Wild-type_versus_KO
Upregulated in class	KO
GeneSet	PLCEPATHWAY
Enrichment Score (ES)	-0.63690364
Normalized Enrichment Score (NES)	-1.467414
Nominal p-value	0.053742804
FDR q-value	0.24180055
FWER p-Value	0.352

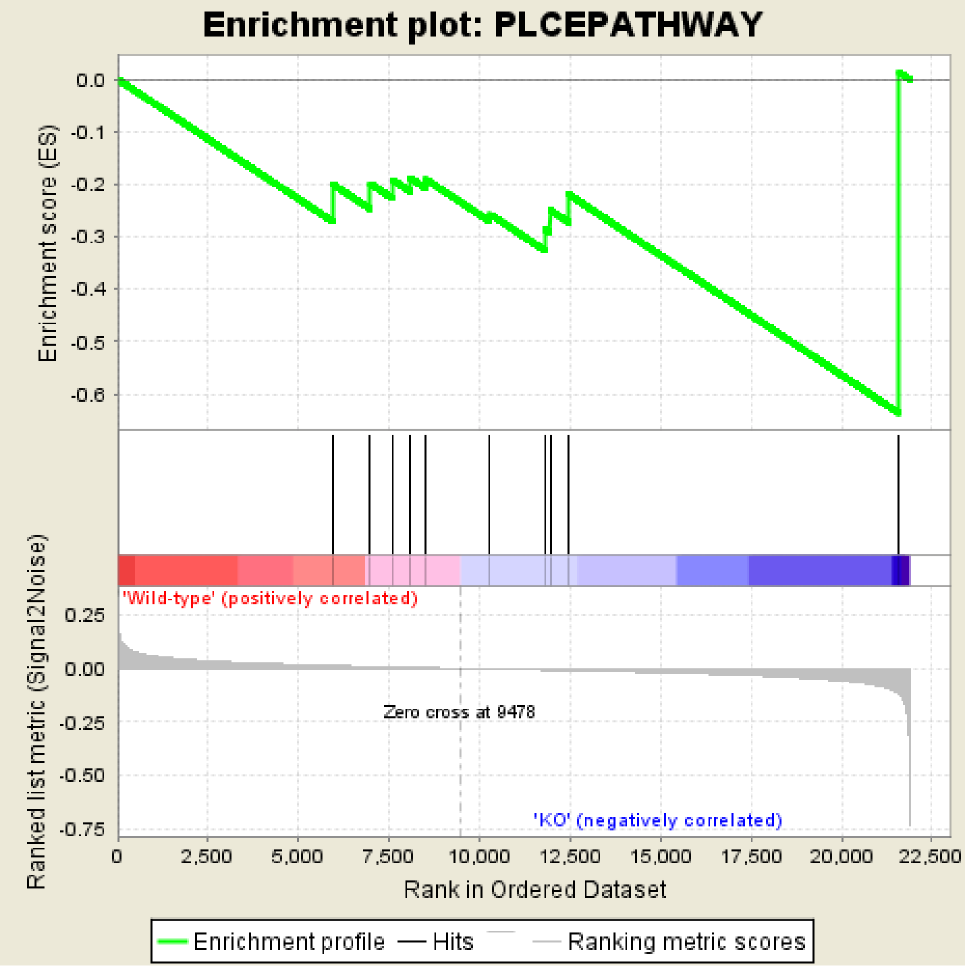

Fig 1: Enrichment plot: PLCEPATHWAY
Profile of the Running ES Score & Positions of GeneSet Members on the Rank Ordered List

Table: GSEA details [plain text format]

	PROBE	GENE SYMBOL	GENE_TITLE	RANK IN GENE LIST	RANK METRIC SCORE	RUNNING ES	CORE ENRICHMENT
1	PRKAR2A	PRKAR2A Entrez, Source, GeneCards	protein kinase, cAMP-dependent, regulatory, type II, alpha	5932	0.013	-0.1996	No
2	GNAS	GNAS Entrez, Source, GeneCards	GNAS complex locus	6980	0.009	-0.1991	No
3	PRKAR1A	PRKAR1A Entrez, Source, GeneCards	protein kinase, cAMP-dependent, regulatory, type I, alpha (tissue specific extinguisher 1)	7609	0.006	-0.1924	No
4	PRKACB	PRKACB Entrez, Source, GeneCards	protein kinase, cAMP-dependent, catalytic, beta	8091	0.005	-0.1887	No
5	ADRB2	ADRB2 Entrez, Source, GeneCards	adrenergic, beta-2-, receptor, surface	8522	0.003	-0.1909	No
6	PRKAR1B	PRKAR1B Entrez, Source, GeneCards	protein kinase, cAMP-dependent, regulatory, type I, beta	10288	-0.003	-0.2574	No
7	RAP2B	RAP2B Entrez, Source, GeneCards	RAP2B, member of RAS oncogene family	11793	-0.007	-0.2856	Yes
8	PRKAR2B	PRKAR2B Entrez, Source, GeneCards	protein kinase, cAMP-dependent, regulatory, type II, beta	11944	-0.008	-0.2491	Yes
9	PTGER1	PTGER1 Entrez, Source, GeneCards	prostaglandin E receptor 1 (subtype EP1), 42kDa	12465	-0.010	-0.2197	Yes
10	PLCE1	PLCE1 Entrez, Source, GeneCards	phospholipase C, epsilon 1	21595	-0.118	0.0135	Yes

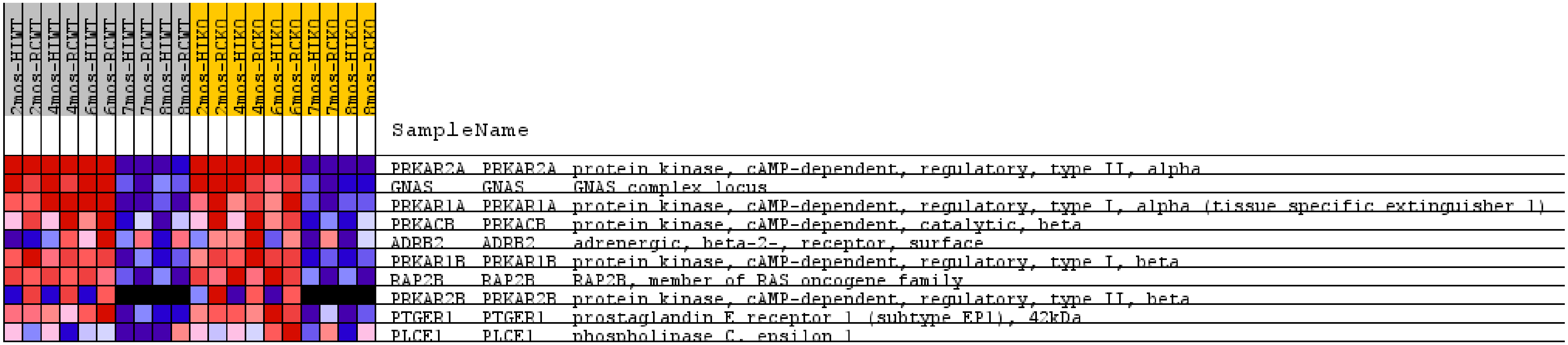

Fig 2: PLCEPATHWAY
Blue-Pink O' Gram in the Space of the Analyzed GeneSet

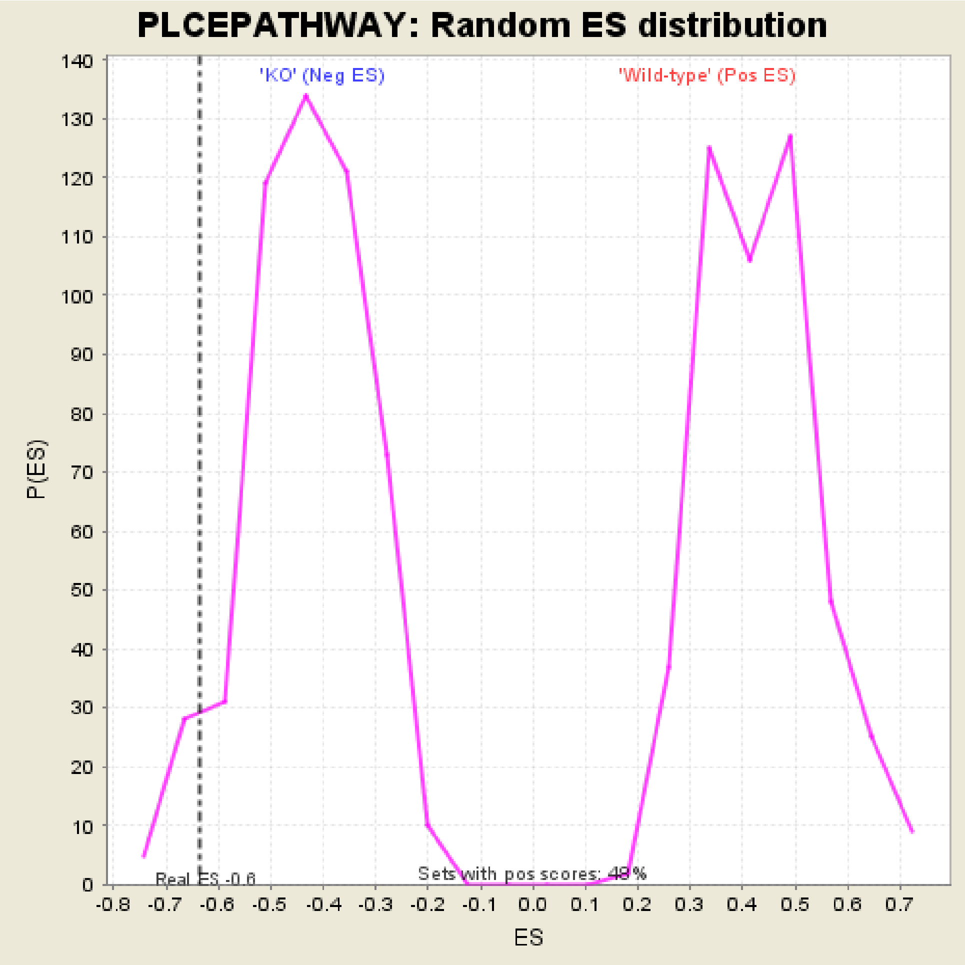

Fig 3: PLCEPATHWAY: Random ES distribution
Gene set null distribution of ES for PLCEPATHWAY

Table: GSEA Results Summary

Dataset	wt vs ko gsea_collapsed_to_symbols.wt vs ko cls file.cls#Wild-type_versus_KO
Phenotype	wt vs ko cls file.cls#Wild-type_versus_KO
Upregulated in class	KO
GeneSet	TGFBPATHWAY
Enrichment Score (ES)	-0.67048216
Normalized Enrichment Score (NES)	-1.4235907
Nominal p-value	0.010141988
FDR q-value	0.16384448
FWER p-Value	0.458

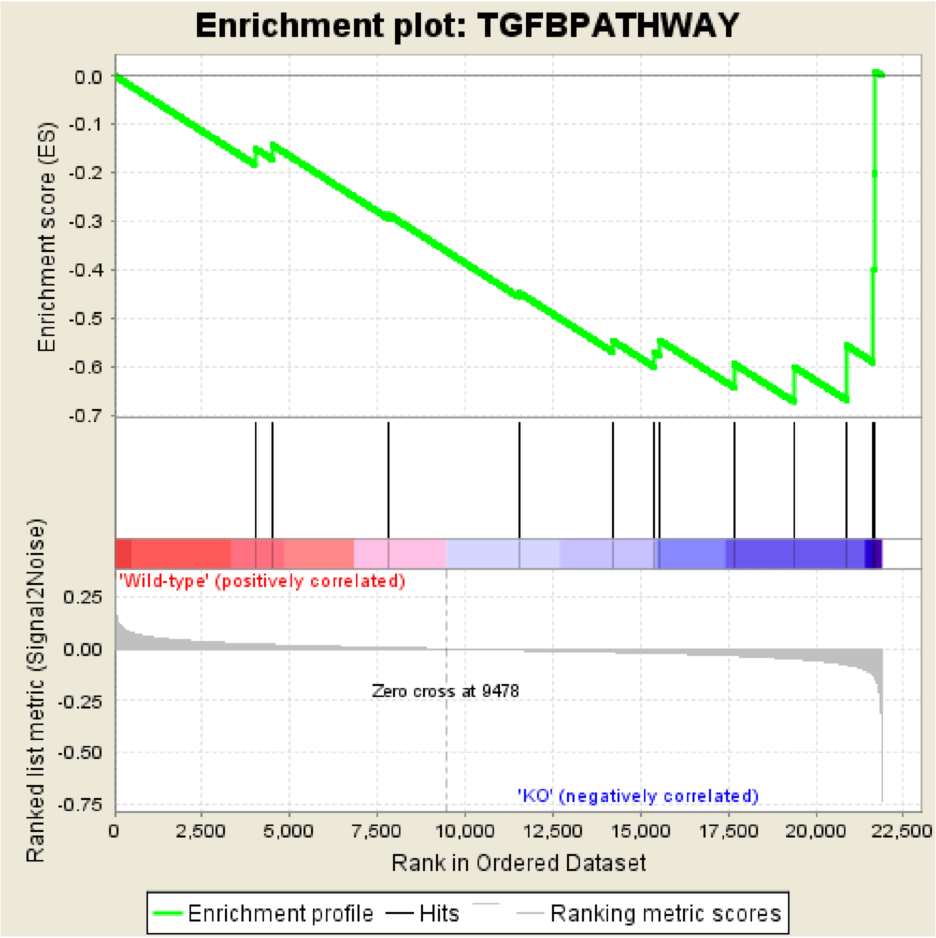

Fig 1: Enrichment plot: TGFBPATHWAY

Profile of the Running ES Score & Positions of GeneSet Members on the Rank Ordered List

Table: GSEA details [plain text format]

	PROBE	GENE SYMBOL	GENE_TITLE	RANK IN GENE LIST	RANK METRIC SCORE	RUNNING ES	CORE ENRICHMENT
1	TGFB3	TGFB3<br>Entrez, Source, GeneCards	transforming growth factor, beta 3	3997	0.023	-0.1491	No
2	CREBBP	CREBBP<br>Entrez, Source, GeneCards	CREB binding protein (Rubinstein-Taybi syndrome)	4496	0.020	-0.1423	No
3	MAP2K1	MAP2K1<br>Entrez, Source, GeneCards	mitogen-activated protein kinase kinase 1	7814	0.006	-0.2856	No
4	MAPK3	MAPK3<br>Entrez, Source, GeneCards	mitogen-activated protein kinase 3	11521	-0.006	-0.4455	No
5	EP300	EP300<br>Entrez, Source, GeneCards	E1A binding protein p300	14225	-0.016	-0.5458	No
6	CDH1	CDH1<br>Entrez, Source, GeneCards	cadherin 1, type 1, E-cadherin (epithelial)	15382	-0.021	-0.5683	No
7	MAP3K7	MAP3K7<br>Entrez, Source, GeneCards	mitogen-activated protein kinase kinase kinase 7	15549	-0.021	-0.5445	No
8	SKIL	SKIL<br>Entrez, Source, GeneCards	SKI-like	17662	-0.033	-0.5924	No
9	MAP3K7IP1	MAP3K7IP1<br>Entrez, Source, GeneCards	mitogen-activated protein kinase kinase kinase 7 interacting protein 1	19372	-0.049	-0.5985	Yes
10	TGFB2	TGFB2<br>Entrez, Source, GeneCards	transforming growth factor, beta 2	20874	-0.077	-0.5546	Yes
11	TGFB1	TGFB1<br>Entrez, Source, GeneCards	transforming growth factor, beta 1 (Camurati-Engelmann disease)	21650	-0.131	-0.3976	Yes
12	TGFBR2	TGFBR2<br>Entrez, Source, GeneCards	transforming growth factor, beta receptor II (70/80kDa)	21675	-0.135	-0.2003	Yes
13	TGFBR1	TGFBR1<br>Entrez, Source, GeneCards	transforming growth factor, beta receptor I (activin A receptor type II-like kinase, 53kDa)	21699	-0.143	0.0087	Yes

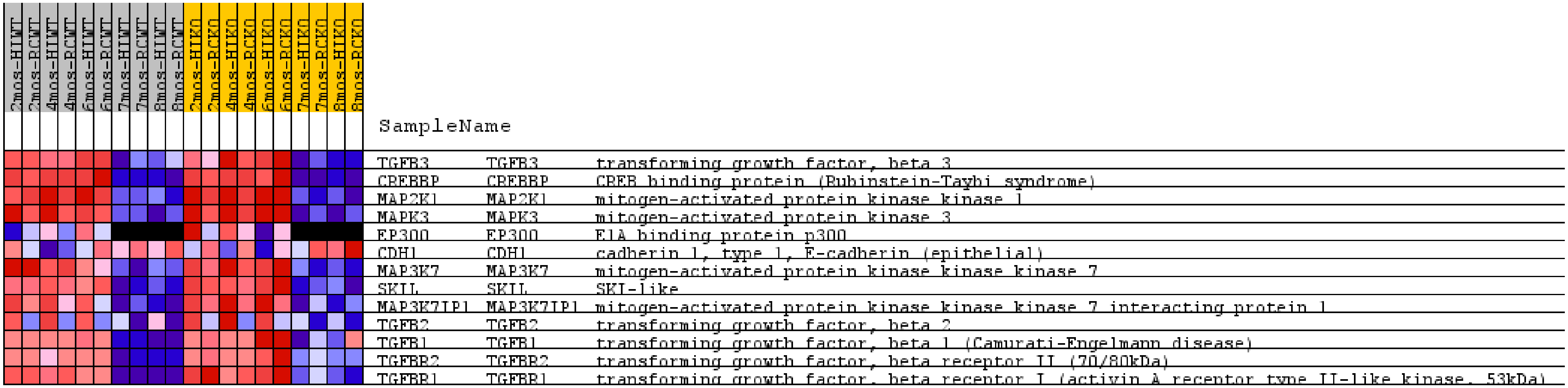

Fig 2: TGFBPATHWAY
Blue-Pink O' Gram in the Space of the Analyzed GeneSet

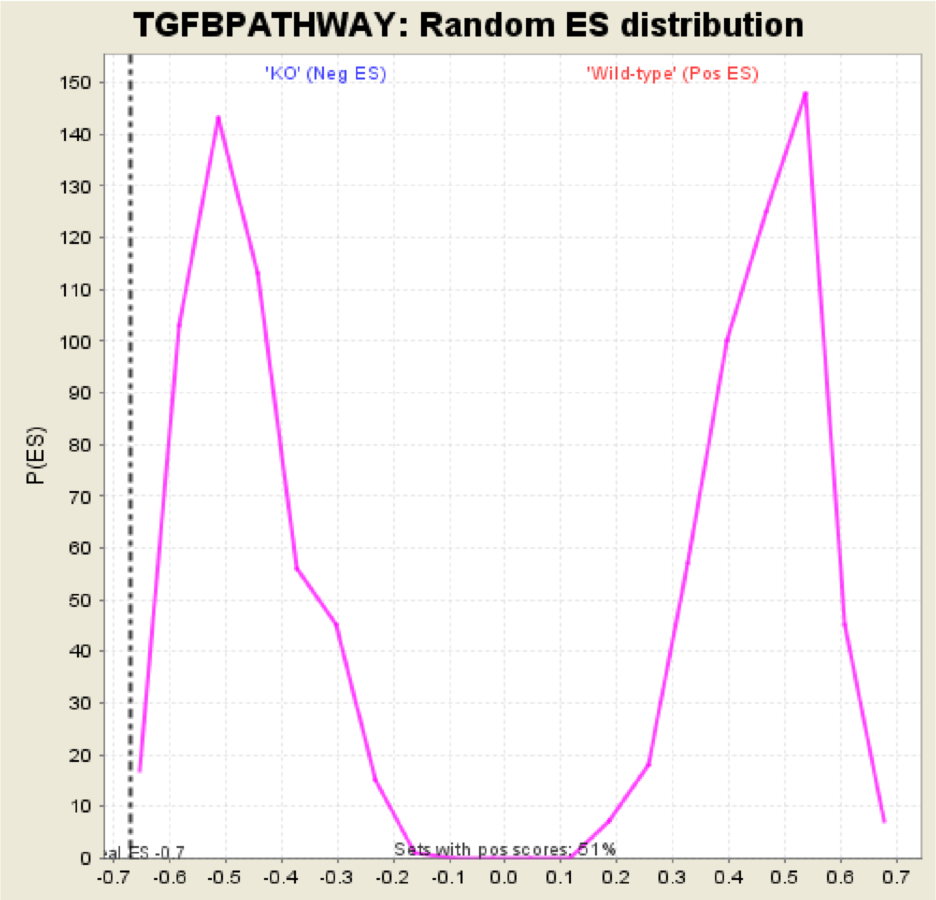

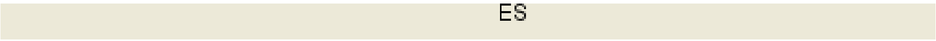

Fig 3: TGFBPATHWAY: Random ES distribution
Gene set null distribution of ES for TGFBPATHWAY

Table: GSEA Results Summary

Dataset	wt vs ko gsea_collapsed_to_symbols.wt vs ko cls file.cls#Wild-type_versus_KO
Phenotype	wt vs ko cls file.cls#Wild-type_versus_KO
Upregulated in class	KO
GeneSet	PLCEPATHWAY
Enrichment Score (ES)	-0.63690364
Normalized Enrichment Score (NES)	-1.467414
Nominal p-value	0.053742804
FDR q-value	0.24180055
FWER p-Value	0.352

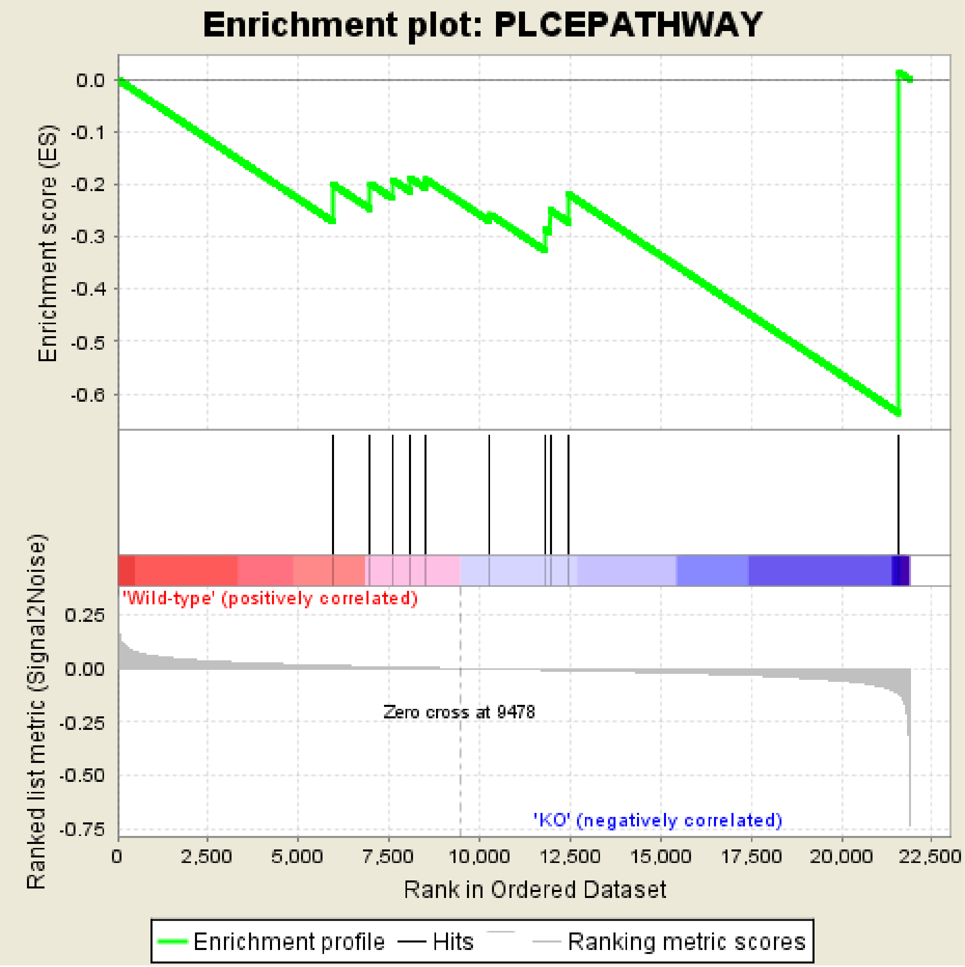

Fig 1: Enrichment plot: PLCEPATHWAY
Profile of the Running ES Score & Positions of GeneSet Members on the Rank Ordered List

Table: GSEA details [plain text format]

	PROBE	GENE SYMBOL	GENE_TITLE	RANK IN GENE LIST	RANK METRIC SCORE	RUNNING ES	CORE ENRICHMENT
1	PRKAR2A	PRKAR2A<br>Entrez, Source, GeneCards	protein kinase, cAMP-dependent, regulatory, type II, alpha	5932	0.013	-0.1996	No
2	GNAS	GNAS<br>Entrez, Source, GeneCards	GNAS complex locus	6980	0.009	-0.1991	No
3	PRKAR1A	PRKAR1A<br>Entrez, Source, GeneCards	protein kinase, cAMP-dependent, regulatory, type I, alpha (tissue specific extinguisher 1)	7609	0.006	-0.1924	No
4	PRKACB	PRKACB<br>Entrez, Source, GeneCards	protein kinase, cAMP-dependent, catalytic, beta	8091	0.005	-0.1887	No
5	ADRB2	ADRB2<br>Entrez, Source, GeneCards	adrenergic, beta-2-, receptor, surface	8522	0.003	-0.1909	No
6	PRKAR1B	PRKAR1B<br>Entrez, Source, GeneCards	protein kinase, cAMP-dependent, regulatory, type I, beta	10288	-0.003	-0.2574	No
7	RAP2B	RAP2B<br>Entrez, Source, GeneCards	RAP2B, member of RAS oncogene family	11793	-0.007	-0.2856	Yes
8	PRKAR2B	PRKAR2B<br>Entrez, Source, GeneCards	protein kinase, cAMP-dependent, regulatory, type II, beta	11944	-0.008	-0.2491	Yes
9	PTGER1	PTGER1<br>Entrez, Source, GeneCards	prostaglandin E receptor 1 (subtype EP1), 42kDa	12465	-0.010	-0.2197	Yes
10	PLCE1	PLCE1<br>Entrez, Source, GeneCards	phospholipase C, epsilon 1	21595	-0.118	0.0135	Yes

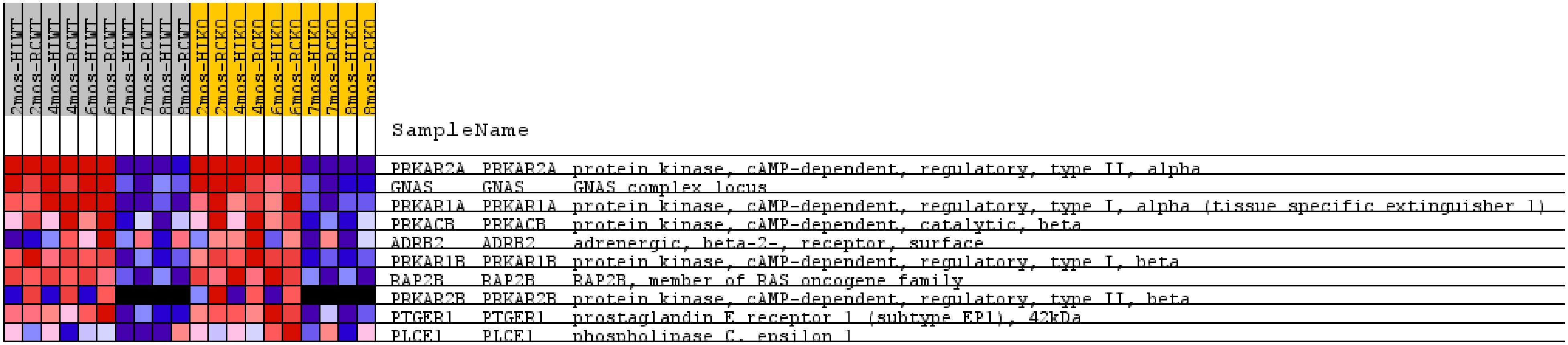

Fig 2: PLCEPATHWAY
Blue-Pink O' Gram in the Space of the Analyzed GeneSet

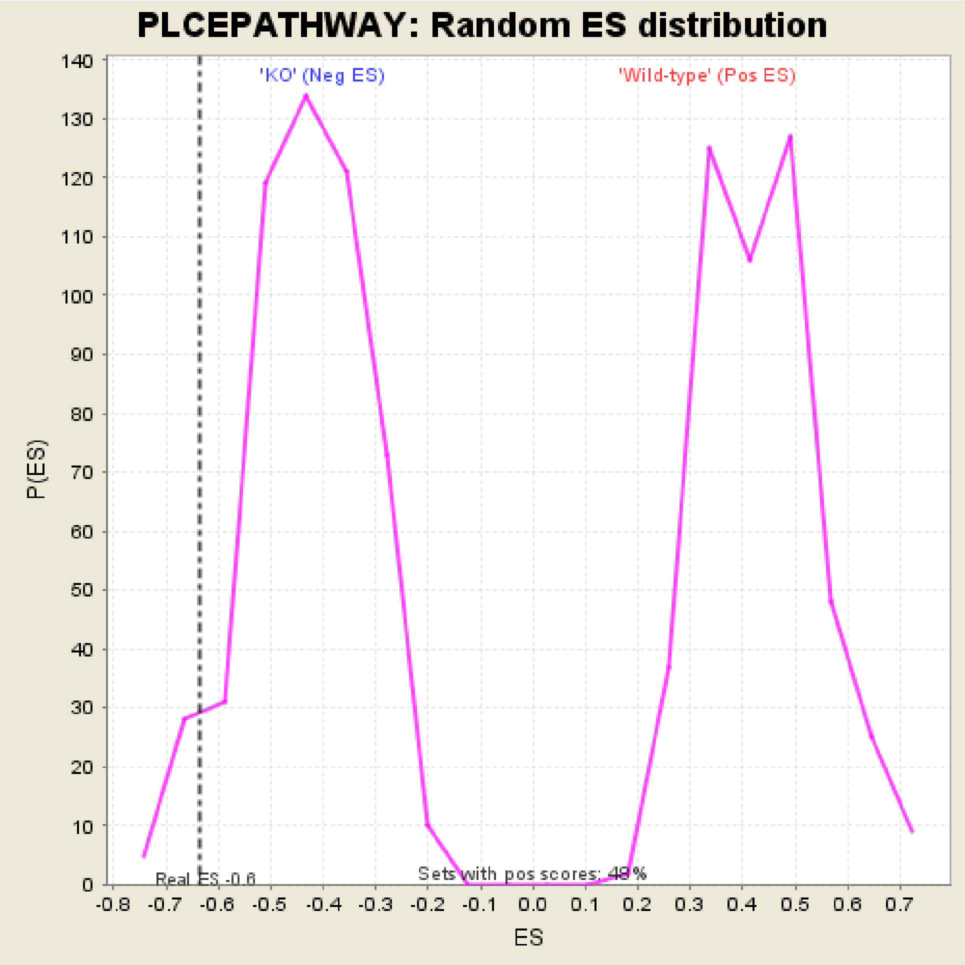

Fig 3: PLCEPATHWAY: Random ES distribution
Gene set null distribution of ES for PLCEPATHWAY

Table: GSEA Results Summary

Dataset	wt vs ko gsea_collapsed_to_symbols.wt vs ko cls file.cls#Wild-type_versus_KO
Phenotype	wt vs ko cls file.cls#Wild-type_versus_KO
Upregulated in class	KO
GeneSet	TOB1PATHWAY
Enrichment Score (ES)	-0.7317913
Normalized Enrichment Score (NES)	-1.6300206
Nominal p-value	0.0040816325
FDR q-value	0.08729656
FWER p-Value	0.134

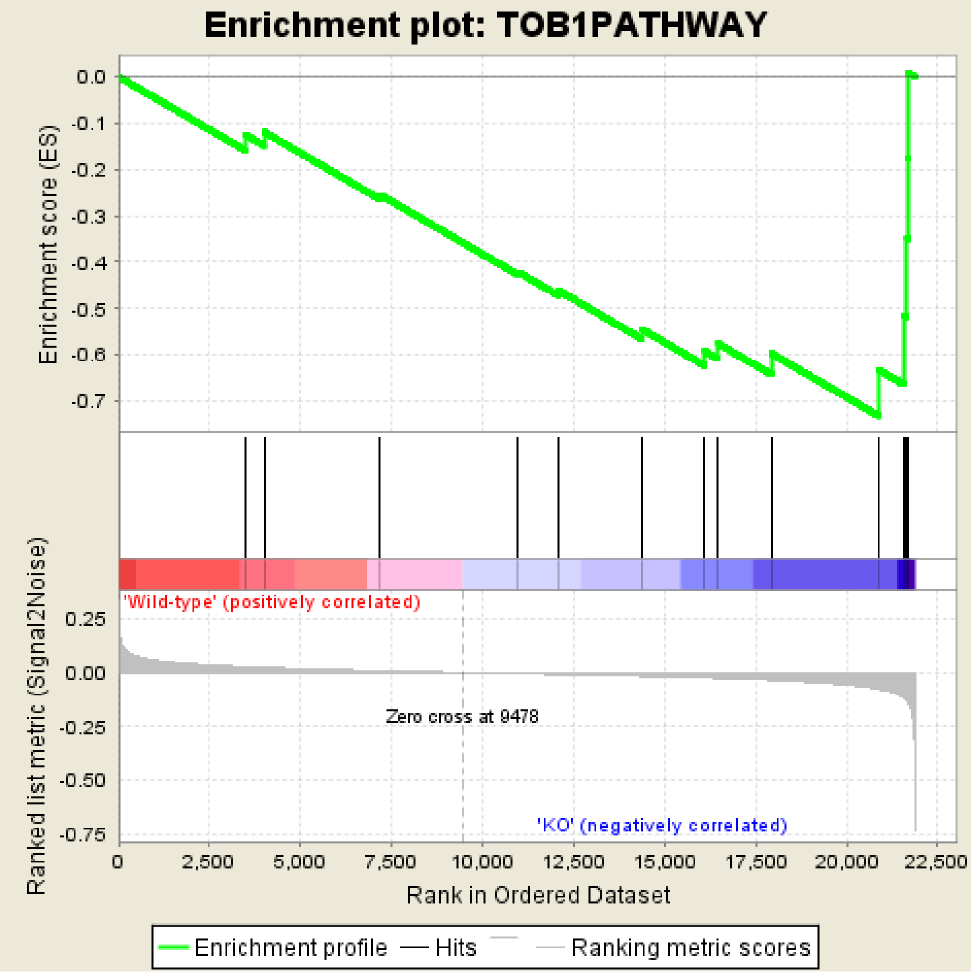

Fig 1: Enrichment plot: TOB1PATHWAY
Profile of the Running ES Score & Positions of GeneSet Members on the Rank Ordered List

Table: GSEA details [plain text format]

	PROBE	GENE SYMBOL	GENE_TITLE	RANK IN GENE LIST	RANK METRIC SCORE	RUNNING ES	CORE ENRICHMENT
1	TOB1	TOB1<br>Entrez, Source, GeneCards	transducer of ERBB2, 1	3490	0.026	-0.1256	No
2	TGFB3	TGFB3<br>Entrez, Source, GeneCards	transforming growth factor, beta 3	3997	0.023	-0.1191	No
3	TOB2	TOB2<br>Entrez, Source, GeneCards	transducer of ERBB2, 2	7186	0.008	-0.2545	No
4	CD3E	CD3E<br>Entrez, Source, GeneCards	CD3e molecule, epsilon (CD3-TCR complex)	10980	-0.005	-0.4217	No
5	IL4	IL4<br>Entrez, Source, GeneCards	interleukin 4	12079	-0.008	-0.4612	No
6	IFNG	IFNG<br>Entrez, Source, GeneCards	interferon, gamma	14357	-0.016	-0.5441	No
7	IL2RA	IL2RA<br>Entrez, Source, GeneCards	interleukin 2 receptor, alpha	16099	-0.024	-0.5926	No
8	CD3D	CD3D<br>Entrez, Source, GeneCards	CD3d molecule, delta (CD3-TCR complex)	16474	-0.026	-0.5761	No
9	TGFBR3	TGFBR3<br>Entrez, Source, GeneCards	transforming growth factor, beta receptor III (betaglycan, 300kDa)	17925	-0.035	-0.5970	No
10	TGFB2	TGFB2<br>Entrez, Source, GeneCards	transforming growth factor, beta 2	20874	-0.077	-0.6326	Yes
11	CD3G	CD3G<br>Entrez, Source, GeneCards	CD3g molecule, gamma (CD3-TCR complex)	21570	-0.116	-0.5152	Yes
12	TGFB1	TGFB1<br>Entrez, Source, GeneCards	transforming growth factor, beta 1 (Camurati-Engelmann disease)	21650	-0.131	-0.3492	Yes
13	TGFBR2	TGFBR2<br>Entrez, Source, GeneCards	transforming growth factor, beta receptor II (70/80kDa)	21675	-0.135	-0.1754	Yes
14	TGFBR1	TGFBR1<br>Entrez, Source, GeneCards	transforming growth factor, beta receptor I (activin A receptor type II-like kinase, 53kDa)	21699	-0.143	0.0087	Yes

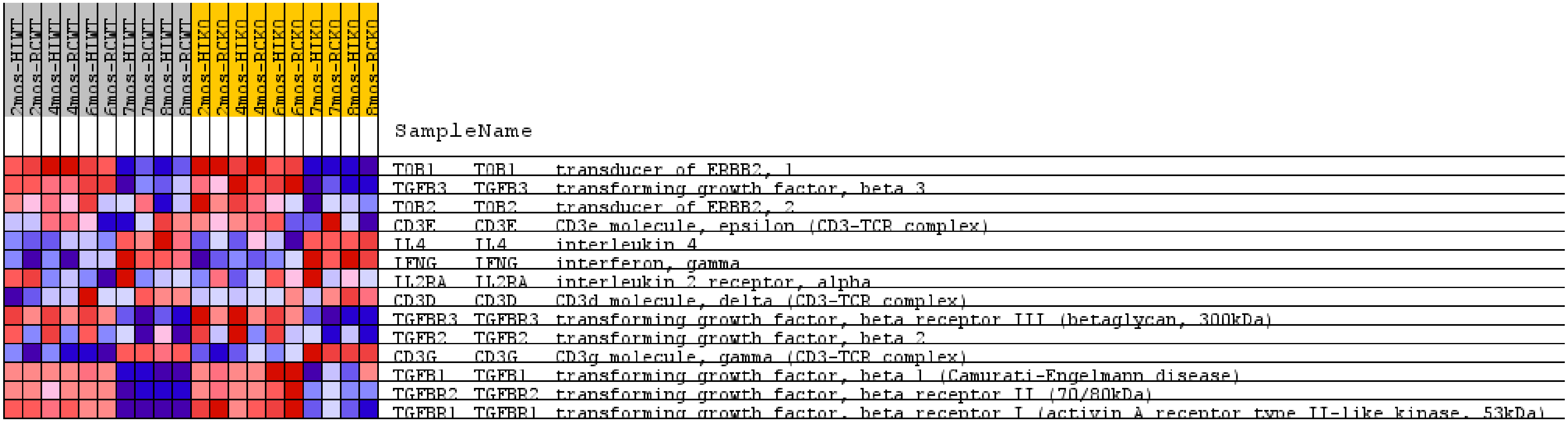

Fig 2: TOB1PATHWAY
Blue-Pink O' Gram in the Space of the Analyzed GeneSet

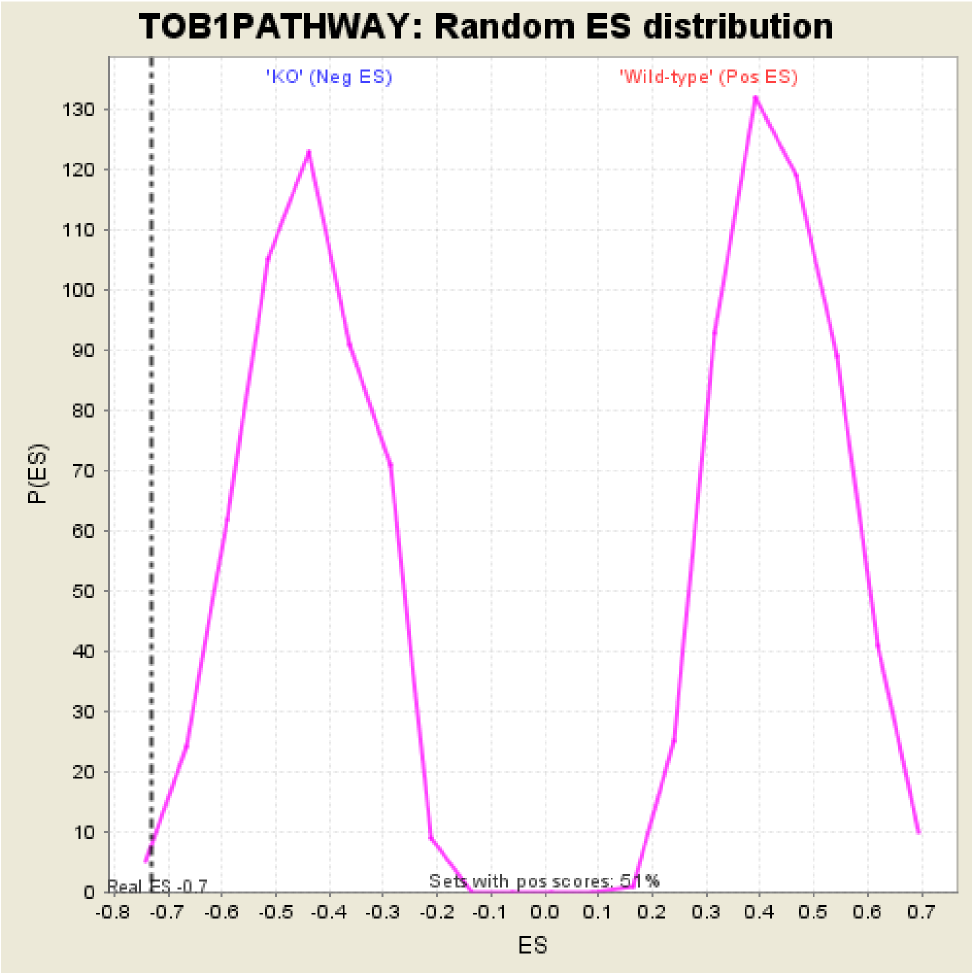

Fig 3: TOB1PATHWAY: Random ES distribution
Gene set null distribution of ES for TOB1PATHWAY

Table: GSEA Results Summary

Dataset	wt vs ko gsea_collapsed_to_symbols.wt vs ko cls file.cls#Wild-type_versus_KO
Phenotype	wt vs ko cls file.cls#Wild-type_versus_KO
Upregulated in class	KO
GeneSet	VITCBPATHWAY
Enrichment Score (ES)	-0.5132887
Normalized Enrichment Score (NES)	-1.4545459
Nominal p-value	0.03877551
FDR q-value	0.22819489
FWER p-Value	0.377

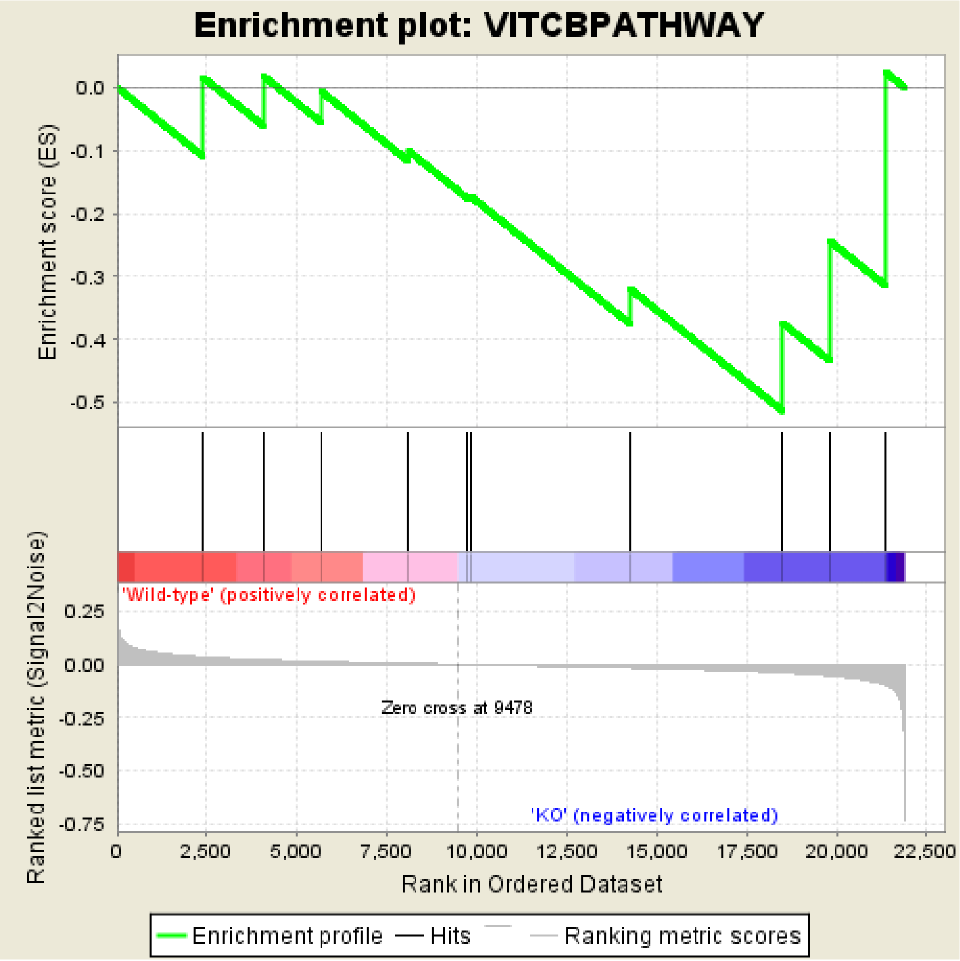

Fig 1: Enrichment plot: VITCBPATHWAY
Profile of the Running ES Score & Positions of GeneSet Members on the Rank Ordered List

Table: GSEA details [plain text format]

	PROBE	GENE SYMBOL	GENE_TITLE	RANK IN GENE LIST	RANK METRIC SCORE	RUNNING ES	CORE ENRICHMENT
1	SLC23A1	SLC23A1 Entrez, Source, GeneCards	solute carrier family 23 (nucleobase transporters), member 1	2387	0.036	0.0161	No
2	COL4A5	COL4A5 Entrez, Source, GeneCards	collagen, type IV, alpha 5 (Alport syndrome)	4055	0.023	0.0187	No
3	COL4A6	COL4A6 Entrez, Source, GeneCards	collagen, type IV, alpha 6	5678	0.014	-0.0063	No
4	SLC2A1	SLC2A1 Entrez, Source, GeneCards	solute carrier family 2 (facilitated glucose transporter), member 1	8102	0.005	-0.1009	No
5	SLC2A3	SLC2A3 Entrez, Source, GeneCards	solute carrier family 2 (facilitated glucose transporter), member 3	9745	-0.001	-0.1730	No
6	SLC23A2	SLC23A2 Entrez, Source, GeneCards	solute carrier family 23 (nucleobase transporters), member 2	9821	-0.001	-0.1726	No
7	P4HB	P4HB Entrez, Source, GeneCards	procollagen-proline, 2-oxoglutarate 4-dioxygenase (proline 4-hydroxylase), beta polypeptide	14259	-0.016	-0.3195	No
8	COL4A3	COL4A3 Entrez, Source, GeneCards	collagen, type IV, alpha 3 (Goodpasture antigen)	18500	-0.040	-0.3740	Yes
9	COL4A2	COL4A2 Entrez, Source, GeneCards	collagen, type IV, alpha 2	19806	-0.055	-0.2434	Yes
10	COL4A4	COL4A4 Entrez, Source, GeneCards	collagen, type IV, alpha 4	21360	-0.097	0.0242	Yes

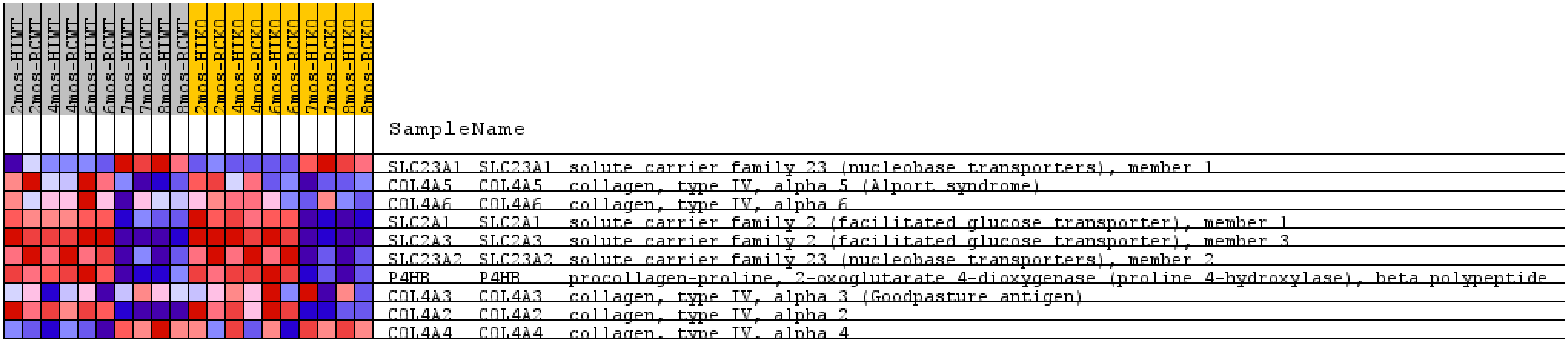

Fig 2: VITCBPATHWAY
Blue-Pink O' Gram in the Space of the Analyzed GeneSet

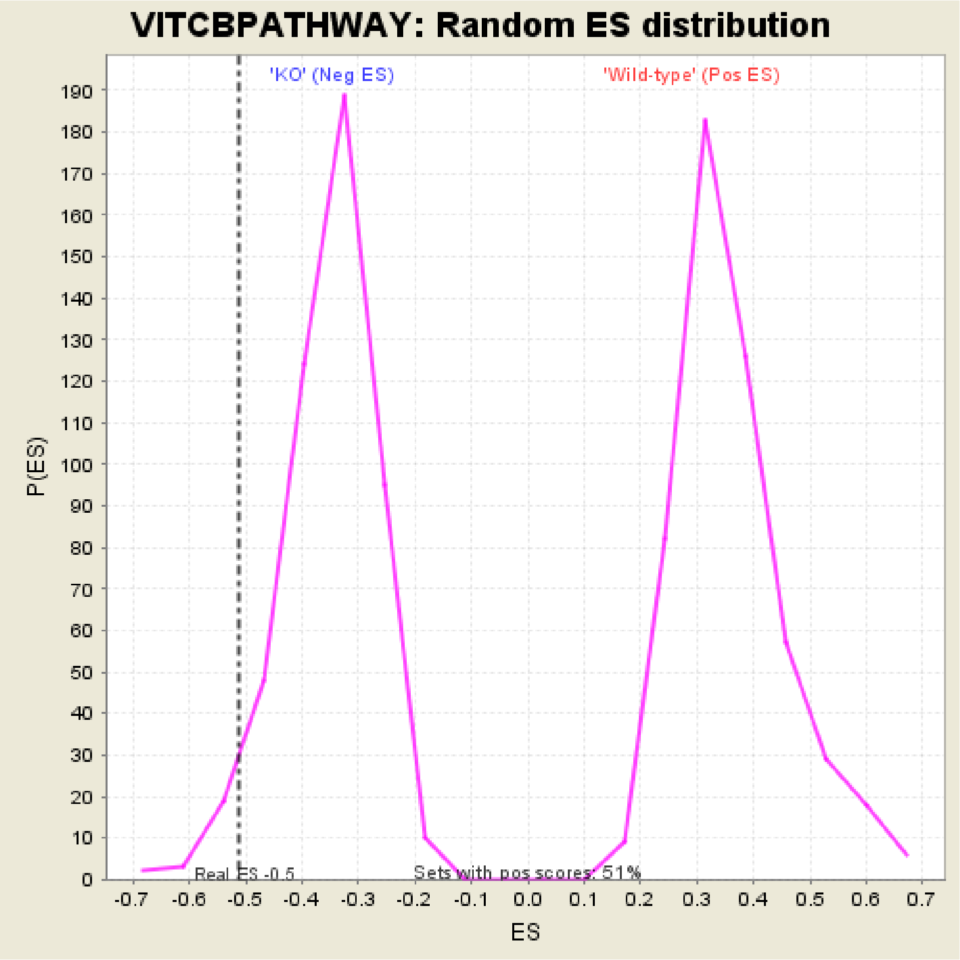

Fig 3: VITCBPATHWAY: Random ES distribution
Gene set null distribution of ES for VITCBPATHWAY
