# Supplementary material for: Molecular signatures of neurodegeneration in the cortex of PS1/PS2 double knockout mice
Source: Mol Neurodegener. 2008 Oct 3;3:14. doi: 10.1186/1750-1326-3-14 (PMC2569036; doi:10.1186/1750-1326-3-14)
Supplement: Additional file 4 — Gene expression differences in the frontal cortex (FC) of PSKO and CNT mice. A gene probe was differentially expressed if it reported > 50% change (|ALR| > 0.585) at a pairwise t-test p < 0.05 between the FC of the PSKO and CNT samples. 70 genes were upregulated, while only 3 genes showed reduction in the PSKO samples. The probes from this list are clustered in Figure 2. [file 1750-1326-3-14-S4.pdf]

TABLE 2. Expression differences between PS1PS2KO and CNT in the FRONTAL CORTEX (>50%, p<0.05)

Probe Set	Gene Title	Symbol	Unigene	NCBI ID	ALR all	ALR HC	ALR FC	pVal ALL	PrPval HC	PrPval FC
1417851_at	chemokine (C-X-C motif) ligand 13	Cxcl13	Mm.10116.1	AF030636	**2.31**	**2.02**	**2.59**	*0.00452*	*0.13134*	*0.02250*
1419202_at	cystatin F (leukocystatin)	Cst7	Mm.12965.1	NM_009977	**1.97**	**1.41**	**2.53**	*0.00143*	*0.08791*	*0.00992*
1417266_at	chemokine (C-C motif) ligand 6	Ccl6	Mm.137.1	BC002073	**1.58**	**1.23**	**1.92**	*0.00025*	*0.04007*	*0.00373*
1420699_at	C-type lectin domain family 7, member a	Clec7a	Mm.132943.1	NM_020008	**1.47**	**1.20**	**1.75**	*0.00100*	*0.06092*	*0.01402*
1426509_s_at	glial fibrillary acidic protein	Gfap	Mm.1239.1	BB183081	**1.43**	**1.13**	**1.73**	*0.00124*	*0.04247*	*0.02356*
1426508_at	glial fibrillary acidic protein	Gfap	Mm.1239.1	BB183081	**1.32**	**0.98**	**1.67**	*0.00260*	*0.05941*	*0.03135*
1418021_at	complement component 4B	C4b	Mm.16106.1	NM_009780	**1.34**	**1.07**	**1.61**	*0.00507*	*0.11087*	*0.03941*
1448859_at	chemokine (C-X-C motif) ligand 13	Cxcl13	Mm.10116.1	AF030636	**1.32**	**1.07**	**1.56**	*0.00426*	*0.13192*	*0.02315*
1450792_at	TYRO protein tyrosine kinase binding protein	Tyrobp	Mm.46301.1	NM_011662	**1.34**	**1.13**	**1.56**	*0.00088*	*0.06521*	*0.01056*
1436996_x_at	lysozyme	Lyz	Mm.45436.2	AV066625	**1.16**	**0.79**	**1.54**	*0.00803*	*0.15150*	*0.03980*
1423547_at	lysozyme	Lyzs	Mm.45436.1	AW208566	**1.11**	**0.71**	**1.51**	*0.00851*	*0.12182*	*0.04666*
1419128_at	integrin alpha X	Itgax	Mm.22378.1	NM_021334	**1.00**	**0.57**	**1.43**	*0.00244*	*0.12644*	*0.00716*
1439426_x_at	lysozyme	Lyz	Mm.45436.3	AV058500	**1.06**	**0.74**	**1.37**	*0.00311*	*0.07813*	*0.02788*
1452417_x_at	immunoglobulin kappa chain, constant region	Igk-C	Mm.104747.14	AV057155	**0.84**	**0.38**	**1.30**	*0.11118*	*0.67283*	*0.04517*
1417381_at	complement component 1, q subcomponent, alpha	C1qa	Mm.370.1	NM_007572	**1.11**	**0.94**	**1.28**	*0.00180*	*0.09314*	*0.01369*
1449911_at	lymphocyte-activation gene 3	Lag3	Mm.4528.1	NM_008479	**0.91**	**0.59**	**1.24**	*0.00267*	*0.12388*	*0.01152*
1449164_at	CD68 antigen	Cd68	Mm.15819.1	BC021637	**1.03**	**0.85**	**1.22**	*0.00342*	*0.09923*	*0.02970*
1425519_a_at	CD74 antigen (invariant polypeptide of MHC class II)	Cd74	Mm.7043.1	BC003476	**0.95**	**0.69**	**1.21**	*0.00850*	*0.16952*	*0.03817*
1421792_s_at	triggering receptor expressed on myeloid cells 2	Trem2	Mm.195828.1	NM_031254	**0.93**	**0.66**	**1.20**	*0.00288*	*0.12005*	*0.01573*
1460218_at	CD52 antigen	Cd52	Mm.24130.1	NM_013706	**0.99**	**0.81**	**1.16**	*0.00108*	*0.07929*	*0.00912*
1422903_at	lymphocyte antigen 86	Ly86	Mm.2639.1	NM_010745	**0.87**	**0.60**	**1.13**	*0.00215*	*0.09559*	*0.01465*
1417063_at	complement component 1, q subcomponent, beta	C1qb	Mm.2570.1	NM_009777	**0.97**	**0.84**	**1.10**	*0.00297*	*0.10032*	*0.02402*
1449401_at	complement component 1, q subcomponent, C	C1qc	Mm.3453.1	NM_007574	**0.93**	**0.79**	**1.07**	*0.00185*	*0.07523*	*0.02206*
1433935_at	expressed sequence AU020206	AU020206	Mm.200422.1	BI151331	**0.90**	**0.74**	**1.05**	*0.00219*	*0.06900*	*0.02895*
1435290_x_at	histocompatibility 2, class II antigen A, alpha	H2-Aa	Mm.175310.2	BE688749	**0.78**	**0.54**	**1.01**	*0.02613*	*0.33849*	*0.03616*
1427076_at	macrophage expressed gene 1	Mpeg1	Mm.3999.1	L20315	**0.93**	**0.85**	**1.01**	*0.00038*	*0.03121*	*0.01347*
1428114_at	solute carrier family 14 (urea transporter), member 1	Slc14a1	Mm.33832.1	AW556396	**0.71**	**0.44**	**0.98**	*0.00222*	*0.08049*	*0.01390*
1437726_x_at	complement component 1, q subcomponent, beta	C1qb	Mm.2570.3	BB111335	**0.96**	**0.94**	**0.98**	*0.00025*	*0.02432*	*0.01219*
1419483_at	complement component 3a receptor 1	C3ar1	Mm.2408.1	NM_009779	**0.82**	**0.66**	**0.98**	*0.00251*	*0.05516*	*0.03805*
1454268_a_at	cytochrome b-245, alpha polypeptide	Cyba	Mm.448.2	AK018713	**0.70**	**0.43**	**0.96**	*0.00208*	*0.06983*	*0.01453*
1416714_at	interferon regulatory factor 8	Irf8	Mm.3182.1	BG069095	**0.69**	**0.44**	**0.94**	*0.00365*	*0.10040*	*0.02244*
1452968_at	collagen triple helix repeat containing 1	Cthrc1	Mm.41556.1	AK003674	**0.96**	**1.00**	**0.92**	*0.00030*	*0.02034*	*0.04006*
1417870_x_at	cathepsin Z	Ctsz	Mm.156919.1	NM_022325	**0.81**	**0.70**	**0.92**	*0.00369*	*0.11343*	*0.02486*
1419482_at	complement component 3a receptor 1	C3ar1	Mm.2408.1	NM_009779	**0.60**	**0.28**	**0.91**	*0.02177*	*0.34027*	*0.03940*
1417868_a_at	cathepsin Z	Ctsz	Mm.156919.1	NM_022325	**0.86**	**0.81**	**0.90**	*0.00256*	*0.08309*	*0.02592*
1448591_at	cathepsin S	Ctss	Mm.3619.1	NM_021281	**0.70**	**0.50**	**0.90**	*0.00214*	*0.12149*	*0.00945*
1416382_at	cathepsin C	Ctsc	Mm.684.1	NM_009982	**0.73**	**0.56**	**0.90**	*0.00157*	*0.05315*	*0.02338*
1419132_at	toll-like receptor 2	Tlr2	Mm.87596.1	NM_011905	**0.65**	**0.42**	**0.89**	*0.00867*	*0.11926*	*0.04823*
1451780_at	B-cell linker	Blnk	Mm.9749.1	AF068182	**0.85**	**0.81**	**0.89**	*0.00379*	*0.08357*	*0.04229*
1416066_at	CD9 antigen	Cd9	Mm.2956.1	NM_007657	**0.77**	**0.67**	**0.87**	*0.00160*	*0.06631*	*0.02275*
1419004_s_at	B-cell leukemia/lymphoma 2 related protein A1	Bcl2a1	Mm.196731.1	L16462	**0.65**	**0.45**	**0.86**	*0.00561*	*0.08845*	*0.04303*
1418340_at	Fc receptor, IgE, high affinity I, gamma polypeptide	Fcer1g	Mm.22673.1	NM_010185	**0.65**	**0.47**	**0.82**	*0.00215*	*0.07978*	*0.02018*
1450678_at	integrin beta 2	Itgb2	Mm.1137.1	NM_008404	**0.63**	**0.44**	**0.82**	*0.00512*	*0.13998*	*0.02652*
1417268_at	CD14 antigen	Cd14	Mm.3460.1	NM_009841	**0.61**	**0.40**	**0.81**	*0.00008*	*0.01818*	*0.00027*
1420361_at	solute carrier family 11, member 1	Slc11a1	Mm.2913.1	NM_013612	**0.57**	**0.37**	**0.78**	*0.01729*	*0.27064*	*0.04271*
1434366_x_at	complement component 1, q subcomponent, beta	C1qb	Mm.2570.2	AW227993	**0.69**	**0.60**	**0.78**	*0.00113*	*0.03548*	*0.03044*
1417869_s_at	cathepsin Z	Ctsz	Mm.156919.1	NM_022325	**0.70**	**0.64**	**0.76**	*0.00157*	*0.04719*	*0.03422*
1437874_s_at	hexosaminidase B	Hexb	Mm.219675.2	AV225808	**0.67**	**0.58**	**0.75**	*0.00171*	*0.07442*	*0.02038*
1436905_x_at	lysosomal-associated protein transmembrane 5	Laptm5	Mm.4554.2	BB218107	**0.71**	**0.69**	**0.73**	*0.00014*	*0.01123*	*0.01530*
1448749_at	pleckstrin	Plek	Mm.98232.1	AF181829	**0.57**	**0.41**	**0.73**	*0.00900*	*0.22278*	*0.02126*
1420249_s_at	chemokine (C-C motif) ligand 6	Ccl6	Mm.218266.1	AV084904	**0.48**	**0.25**	**0.72**	*0.01080*	*0.13946*	*0.03907*
1417346_at	PYD and CARD domain containing	Pycard	Mm.24163.1	BG084230	**0.54**	**0.37**	**0.71**	*0.00328*	*0.07192*	*0.03006*
1418365_at	cathepsin H	Ctsh	Mm.2277.1	NM_007801	**0.61**	**0.52**	**0.71**	*0.00174*	*0.02609*	*0.04492*
1448620_at	Fc receptor, IgG, low affinity III	Fcgr3	Mm.22119.1	NM_010188	**0.58**	**0.47**	**0.69**	*0.00394*	*0.08562*	*0.04041*
1448118_a_at	cathepsin D	Ctsd	Mm.2147.1	NM_009983	**0.64**	**0.60**	**0.69**	*0.00147*	*0.05952*	*0.02417*
1435999_at	serine peptidase inhibitor, Kazal type 8	Spink8	Mm.99613.1	BB284475	**0.39**	**0.10**	**0.67**	*0.19053*	*0.85508*	*0.02674*
1419100_at	serine (or cysteine) peptidase inhibitor, clade A, 3N	Serpina3n	Mm.22650.1	NM_009252	**0.51**	**0.36**	**0.67**	*0.00303*	*0.02803*	*0.04000*
1438629_x_at	granulin	Grn	Mm.1568.2	AV166504	**0.54**	**0.42**	**0.66**	*0.00034*	*0.02322*	*0.01058*
1423909_at	transmembrane protein 176A	Tmem176a	Mm.27061.1	BC010831	**0.52**	**0.38**	**0.65**	*0.00388*	*0.10302*	*0.02962*
1417523_at	pleckstrin	Plek	Mm.98232.1	AF181829	**0.37**	**0.09**	**0.65**	*0.03232*	*0.68526*	*0.00739*
1428018_a_at	Cd300D antigen	Cd300d	Mm.2699.2	AF251705	**0.44**	**0.24**	**0.63**	*0.00332*	*0.12129*	*0.01157*
1416121_at	lysyl oxidase	Lox	Mm.172.1	M65143	**0.32**	**0.03**	**0.62**	*0.05209*	*0.80142*	*0.03354*
1452014_a_at	insulin-like growth factor 1	Igf1	Mm.2770.2	AF440694	**0.36**	**0.11**	**0.62**	*0.01925*	*0.24103*	*0.02914*
1448380_at	lectin, galactoside-binding, soluble 3 binding	Lgals3bp	Mm.3152.1	NM_011150	**0.44**	**0.28**	**0.61**	*0.00906*	*0.11928*	*0.04887*
1421375_a_at	S100 calcium binding protein A6 (calcyclin)	S100a6	Mm.100144.1	NM_011313	**0.57**	**0.53**	**0.60**	*0.00036*	*0.02383*	*0.01769*
1450355_a_at	capping protein (actin filament), gelsolin-like	Capg	Mm.18626.1	NM_007599	**0.41**	**0.22**	**0.60**	*0.01610*	*0.32483*	*0.02581*
1448617_at	CD53 antigen	Cd53	Mm.2692.1	NM_007651	**0.51**	**0.42**	**0.60**	*0.00193*	*0.08527*	*0.01867*
1425545_x_at	histocompatibility 2, D region locus 1	H2-D1	Mm.33263.2	M86502	**0.50**	**0.40**	**0.60**	*0.01362*	*0.25177*	*0.01935*
1448452_at	interferon regulatory factor 8	Irf8	Mm.3182.1	BG069095	**0.50**	**0.40**	**0.60**	*0.00319*	*0.06613*	*0.04074*
1448148_at	granulin	Grn	Mm.1568.1	M86736	**0.53**	**0.46**	**0.59**	*0.00032*	*0.01122*	*0.02192*
1418610_at	solute carrier family 17, member 6	Slc17a6	Mm.70945.1	NM_080853	**-0.68**	**-0.78**	**-0.59**	*0.00014*	*0.01017*	*0.01269*
1440464_at	ELAV (embryonic lethal, abnormal vision)-like 1	Elavl1	Mm.127901.1	BB284404	**-0.83**	**-0.95**	**-0.72**	*0.05498*	*0.32224*	*0.02987*
1439332_at	DNA-damage-inducible transcript 4-like	Ddit4l	Mm.195809.2	AV251625	**-0.50**	**-0.21**	**-0.78**	*0.00511*	*0.27400*	*0.00263*
