# Supplementary material for: Molecular signatures of neurodegeneration in the cortex of PS1/PS2 double knockout mice
Source: Mol Neurodegener. 2008 Oct 3;3:14. doi: 10.1186/1750-1326-3-14 (PMC2569036; doi:10.1186/1750-1326-3-14)
Supplement: Additional file 5 — Gene expression differences in the hippocampus (HC) of PSKO and CNT mice. A gene probe was differentially expressed if it reported > 50% change (|ALR| > 0.585) at a pairwise t-test p < 0.05 between the HC of the PSKO and CNT samples. 36 genes were upregulated, while 101 genes showed reduction in the PSKO samples. The probes from this list are clustered in Figure 3. [file 1750-1326-3-14-S5.pdf]

TABLE 3. Expression differences between PS1PS2KO and CNT in the HIPPOCAMPUS (>50%, p<0.05)

Probe Set	Gene Title	Symbol	Unigene	NCBI ID	ALR all	ALR HC	ALR FC	pVal ALL	PrPval HC	PrPval FC
1454866_s_at	chloride intracellular channel 6	Clic6	Mm.44747.1	BQ176424	1.49	3.03	-0.06	0.09138	0.01047	0.84647
1460049_s_at	RIKEN cDNA 1500015O10 gene	1500015O10R	Mm.213028.1	BB392676	1.24	2.32	0.16	0.07481	0.02017	0.77927
1438200_at	sulfatase 1	Sulf1	Mm.45563.1	BB065799	0.75	1.24	0.26	0.02865	0.01863	0.28295
1417266_at	chemokine (C-C motif) ligand 6	Ccl6	Mm.137.1	BC002073	1.58	1.23	1.92	0.00025	0.04007	0.00373
1426509_s_at	glial fibrillary acidic protein	Gfap	Mm.1239.1	BB183081	1.43	1.13	1.73	0.00124	0.04247	0.02356
1440807_at	Membrane associated guanylate kinase 2	Magi2	Mm.132480.1	BB337886	0.86	1.05	0.66	0.03780	0.02008	0.40748
1429135_at	RIKEN cDNA 1110059M19 gene	1110059M19R	Mm.23496.1	AV015858	0.69	1.03	0.34	0.02029	0.03778	0.25963
1429846_at	RIKEN cDNA 9030411K21 gene	9030411K21R	Mm.100469.1	BM250766	0.89	1.03	0.76	0.00464	0.02851	0.15935
1452968_at	collagen triple helix repeat containing 1	Cthrc1	Mm.41556.1	AK003674	0.96	1.00	0.92	0.00030	0.02034	0.04006
1437726_x_at	complement component 1, q subcomponent, beta	C1qb	Mm.2570.3	BB111335	0.96	0.94	0.98	0.00025	0.02432	0.01219
1437060_at	olfactomedin 4	Olfm4	Mm.26456.1	AV290148	0.47	0.94	-0.01	0.11200	0.04006	0.95601
1445555_at	transient receptor potential cation channel M3	Trpm3	Mm.48730.1	AW125244	0.52	0.93	0.10	0.04566	0.00290	0.51958
1443770_x_at	---	BB026407	Mm.127920.1	BB026407	0.45	0.91	0.00	0.11684	0.03607	0.99130
1446772_at	---	BB453864	Mm.215970.1	BB453864	0.67	0.87	0.47	0.00352	0.01143	0.12967
1456923_at	transient receptor potential cation channel M3	Trpm3	Mm.127343.1	BB377721	0.27	0.87	-0.33	0.40039	0.01051	0.29035
1427076_at	macrophage expressed gene 1	Mpeg1	Mm.3999.1	L20315	0.93	0.85	1.01	0.00038	0.03121	0.01347
1415894_at	ectonucleotide phosphodiesterase 2	Enpp2	Mm.28107.1	BC003264	0.42	0.84	0.01	0.06130	0.04547	0.90588
1423400_at	klotho	Kl	Mm.6500.1	BQ175355	0.54	0.75	0.33	0.05750	0.04344	0.48811
1441102_at	prolactin receptor	Prlr	Mm.33737.1	BB429201	0.37	0.75	-0.01	0.09367	0.02066	0.94457
1456440_s_at	ST8 a-N-acetyl-neuraminide alpha-2,8-sialyltransf 6	St8sia6	Mm.24267.2	AV375081	0.63	0.72	0.53	0.02041	0.00816	0.31737
1428485_at	carbonic anyhydrase 12	Car12	Mm.21397.1	AK009873	0.29	0.71	-0.13	0.12441	0.01155	0.34749
1436905_x_at	lysosomal-associated protein transmembrane 5	Laptm5	Mm.4554.2	BB218107	0.71	0.69	0.73	0.00014	0.01123	0.01530
1441679_at	---	BB126796	Mm.213317.1	BB126796	0.34	0.67	0.00	0.13751	0.03044	0.98642
1454653_at	copine family member IX	Cpne9	Mm.65597.1	BB274531	0.14	0.66	-0.38	0.62151	0.01389	0.25710
1456633_at	transient receptor potential cation channel M3	Trpm3	Mm.131943.1	BB313276	0.30	0.65	-0.04	0.12272	0.01229	0.78653
1424684_at	RAB5C, member RAS oncogene family	Rab5c	Mm.29829.1	BC023027	0.29	0.64	-0.05	0.09196	0.03337	0.62671
1417869_s_at	cathepsin Z	Ctsz	Mm.156919.1	NM_022325	0.70	0.64	0.76	0.00157	0.04719	0.03422
1448556_at	prolactin receptor	Prlr	Mm.2752.1	BC005555	0.33	0.63	0.03	0.05325	0.04917	0.60271
1434719_at	alpha-2-macroglobulin	A2m	Mm.30151.1	BB185854	0.57	0.63	0.50	0.00029	0.00158	0.05596
1420720_at	neuronal pentraxin 2	Nptx2	Mm.10099.1	NM_016789	0.38	0.62	0.14	0.02269	0.03879	0.31888
1433734_at	solute carrier family 13, member 4	Slc13a4	Mm.23666.1	BB192951	0.25	0.62	-0.12	0.20677	0.01648	0.14206
1456435_at	MORN repeat containing 1	Morn1	Mm.19009.1	AI425983	0.49	0.61	0.37	0.00509	0.01583	0.17704
1422124_a_at	protein tyrosine phosphatase, receptor type, C	Ptprc	Mm.143846.1	NM_011210	0.71	0.61	0.81	0.00493	0.04972	0.07405
1438643_at	Calcium/calmodulin-dependent protein kinase ID	Camk1d	Mm.133375.1	BB230839	0.32	0.61	0.04	0.07724	0.01478	0.81078
1418047_at	neurogenic differentiation 6	Neurod6	Mm.5106.1	NM_009717	0.32	0.60	0.03	0.04949	0.03655	0.76196
1434366_x_at	complement component 1, q subcomponent, beta	C1qb	Mm.2570.2	AW227993	0.69	0.60	0.78	0.00113	0.03548	0.03044
1439026_at	transient receptor potential cation channel M3	Trpm3	Mm.92048.1	BB125842	0.44	0.59	0.29	0.00474	0.00756	0.15212
1458378_at	glutamate receptor ionotropic, NMDA3A	Grin3a	Mm.132574.1	AV325957	-0.32	-0.59	-0.05	0.04855	0.00182	0.48771
1431684_at	RIKEN cDNA 4933402J24 gene	4933402J24R	Mm.196294.1	AK016622	-0.39	-0.59	-0.18	0.01067	0.00890	0.08628
1426115_a_at	potassium inwardly-rectifying channel, subfamily J9	Kcnj9	Mm.4278.2	AF130860	-0.45	-0.59	-0.30	0.00300	0.03806	0.02622
1416702_at	serine peptidase inhibitor, clade I, member 1	Serpini1	Mm.41560.1	NM_009250	-0.31	-0.59	-0.04	0.04944	0.04080	0.70819
1444020_at	neurocan	Ncan	Mm.156790.1	BM939365	-0.40	-0.59	-0.20	0.01182	0.03530	0.02830
1431064_at	dipeptidylpeptidase 8	Dpp8	Mm.202749.1	BF119821	-0.36	-0.59	-0.14	0.02217	0.00553	0.25898
1458518_at	cytoplasmic polyadenylation element binding 2	Cpeb2	Mm.151602.1	BM935843	-0.40	-0.60	-0.20	0.02995	0.01552	0.43622
1444139_at	DNA-damage-inducible transcript 4-like	Ddit4l	Mm.205420.1	BG797099	-0.68	-0.60	-0.76	0.00392	0.03772	0.10333
1431229_at	imprinted gene in the Prader-Willi syndrome region	Ipw	Mm.220871.1	AK019361	-0.42	-0.60	-0.23	0.01346	0.00086	0.29805
1440390_at	cDNA sequence BC025575	BC025575	Mm.100100.1	BE980997	-0.39	-0.61	-0.17	0.01278	0.00756	0.05277
1456683_at	SAFB-like, transcription modulator	Sltm	Mm.80600.1	BM200011	-0.55	-0.61	-0.49	0.00364	0.01662	0.15306
1428376_at	RIKEN cDNA 4932415G12 gene	4932415G12R	Mm.116284.1	AK015136	-0.41	-0.61	-0.21	0.01218	0.02455	0.14448
1438205_at	RIKEN cDNA 1110014N23 gene	1110014N23R	Mm.29451.2	BE692418	-0.37	-0.61	-0.12	0.02899	0.01992	0.24160
1456304_at	gene model 996, (NCBI)	Gm996	Mm.44116.1	BF463551	-0.40	-0.62	-0.18	0.02139	0.04583	0.11569
1422331_at	POU domain, class 3, transcription factor 3	Pou3f3	Mm.56944.1	NM_008900	-0.39	-0.62	-0.17	0.00419	0.00322	0.21468
1435615_at	zinc finger protein 365	Zfp365	Mm.39548.2	BB277790	-0.38	-0.63	-0.13	0.02403	0.00430	0.24051
1447207_at	zyg-ll homolog B (C. elegans)	Zyg11b	Mm.219003.1	BE946949	-0.49	-0.63	-0.35	0.00965	0.04375	0.20127
1429684_at	RIKEN cDNA 5830472M02 gene	5830472M02R	Mm.89828.1	BG094398	-0.46	-0.63	-0.29	0.00869	0.04559	0.12443
1431216_s_at	DnaJ (Hsp40) homolog, subfamily C, member 6	Dnajc6	Mm.181754.1	BI730538	-0.41	-0.63	-0.18	0.01572	0.00585	0.23669
1429696_at	G protein-coupled receptor 123	Gpr123	Mm.39863.1	BE946247	-0.46	-0.63	-0.29	0.00763	0.00917	0.20047
1434885_at	SPT2, Suppressor of Ty, domain containing 1	Spty2d1	Mm.24055.1	BM242524	-0.34	-0.63	-0.05	0.09042	0.03486	0.81331
1443805_at	dapper homolog 3, antagonist of beta-catenin	Dact3	Mm.65304.1	AW122309	-0.44	-0.64	-0.24	0.00725	0.01972	0.05822
1431465_s_at	forty-two-three domain containing 1	Fyttd1	Mm.195995.1	AK008130	-0.48	-0.65	-0.32	0.00697	0.02416	0.17016
1422949_at	nitric oxide synthase 1, neuronal	Nos1	Mm.44249.1	NM_008712	-0.26	-0.65	0.13	0.13029	0.01141	0.31277
1459750_s_at	G protein-coupled receptor 123	Gpr123	Mm.195308.1	AU015577	-0.49	-0.65	-0.33	0.01531	0.02070	0.30183
1430395_at	ankyrin repeat domain 45	Ankrd45	Mm.87356.1	AK016757	-0.44	-0.65	-0.22	0.01046	0.01552	0.10166
1429187_at	transmembrane emp24 transport domain 7	Tmed7	Mm.220909.1	AV306753	-0.39	-0.65	-0.12	0.03836	0.04615	0.14143
1442099_at	ubiquitin specific peptidase 31	Usp31	Mm.171183.1	BM227490	-0.45	-0.65	-0.24	0.00728	0.00627	0.11215
1427017_at	special AT-rich sequence binding protein 2	Satb2	Mm.73537.1	BB104560	-0.51	-0.66	-0.35	0.00558	0.04735	0.07620
1435877_at	serine/threonine kinase 38 like	Stk38l	Mm.100606.1	BB476811	-0.44	-0.66	-0.22	0.02392	0.04908	0.27982
1435385_at	teashirt zinc finger family member 2	Tshz2	Mm.28740.1	AW556161	-0.24	-0.66	0.19	0.28054	0.00203	0.17376
1443434_s_at	plexin C1	Plxnc1	Mm.189760.1	AV317262	-0.52	-0.67	-0.37	0.00302	0.00600	0.12769
1450041_a_at	tubby candidate gene	Tub	Mm.4804.1	NM_021885	-0.39	-0.68	-0.10	0.03133	0.01312	0.62859
1428052_a_at	zinc finger, MYM domain containing 1	Zmym1	Mm.80623.2	BC027750	-0.57	-0.68	-0.45	0.00393	0.04126	0.08303
1435234_at	nuclear receptor coactivator 2	Ncoa2	Mm.9636.1	BM234716	-0.40	-0.68	-0.12	0.04606	0.02359	0.52424
1460025_at	leucine-rich and immunoglobulin-like domains 2	Lrig2	Mm.122660.1	BB129159	-0.43	-0.68	-0.19	0.01336	0.00962	0.02408
1422151_at	opposite strand transcription unit to Stag3	Gats	Mm.160250.1	NM_030719	-0.45	-0.68	-0.21	0.01173	0.03421	0.17128
1440228_at	RAN binding protein 6	Ranbp6	Mm.138234.1	BB477637	-0.42	-0.68	-0.16	0.02612	0.02937	0.21807
1453760_at	mesoderm induction early response 1 homolog	Mier1	Mm.100190.1	BG963598	-0.40	-0.69	-0.11	0.03805	0.03233	0.23409
1443851_at	RIKEN cDNA 8430415E04 gene	8430415E04R	Mm.151153.1	BE980275	-0.45	-0.69	-0.22	0.01658	0.04884	0.03419
1434112_at	latrophilin 2	Lphn2	Mm.30658.1	BG966339	-0.40	-0.69	-0.11	0.02793	0.00104	0.01476
1450750_a_at	nuclear receptor subfamily 4, group A, member 2	Nr4a2	Mm.3507.1	NM_013613	-0.47	-0.71	-0.23	0.00784	0.01019	0.27450
1442867_at	---	BQ174216	Mm.194351.1	BQ174216	-0.39	-0.71	-0.07	0.10367	0.03395	0.82457
1429362_a_at	splicing factor 3b, subunit 2	Sf3b2	Mm.196532.1	BF022081	-0.40	-0.71	-0.09	0.05242	0.04038	0.35578
1434917_at	cordon-bleu	Cobl	Mm.22847.1	BQ173923	-0.43	-0.71	-0.14	0.03121	0.02830	0.25494
1447944_at	zinc finger with KRAB and SCAN domains 1	Zkscan1	Mm.25477.2	BB006473	-0.53	-0.71	-0.35	0.00603	0.04890	0.06630
1454950_at	RIKEN cDNA B930006L02 gene	B930006L02R	Mm.148313.1	BB699417	-0.44	-0.71	-0.16	0.02910	0.02641	0.29703
1423287_at	cerebellin 1 precursor protein	Cbln1	Mm.4880.1	AA016422	-0.38	-0.71	-0.04	0.04034	0.01442	0.80020
1435134_at	arylacetamide deacetylase-like 1	Aadacl1	Mm.24576.1	AV369935	-0.46	-0.71	-0.20	0.01456	0.01588	0.04136
1441325_at	NF-kappaB repressing factor	Nkrf	Mm.151033.1	BE952938	-0.52	-0.72	-0.32	0.01456	0.04743	0.23025
1450184_s_at	thyrotroph embryonic factor	Tef	Mm.154152.1	NM_017376	-0.39	-0.72	-0.05	0.02412	0.01824	0.24758
1441064_at	RIKEN cDNA E330039K12 gene	E330039K12R	Mm.152355.1	BF467171	-0.44	-0.73	-0.16	0.01956	0.00017	0.13178
1453263_at	MAK10 homolog, amino-acid N-acetyltransferase	Mak10	Mm.195964.1	AK021042	-0.50	-0.73	-0.28	0.00554	0.01015	0.02561
1430144_at	RIKEN cDNA 5830411G16 gene	5830411G16R	Mm.105343.1	BB815600	-0.59	-0.73	-0.45	0.00053	0.01204	0.00490
1453860_s_at	nuclear receptor subfamily 3, group C, member 1	Nr3c1	Mm.220887.1	BB600983	-0.57	-0.73	-0.41	0.01480	0.04249	0.26705
1434374_at	RIKEN cDNA B930006L02 gene	B930006L02R	Mm.148313.1	BB699417	-0.45	-0.74	-0.17	0.02705	0.00911	0.35909
1453224_at	zinc finger, AN1-type domain 5	Zfand5	Mm.48662.1	AW049828	-0.48	-0.74	-0.22	0.01480	0.02131	0.09281
1454250_at	exocyst complex component 6B	Exoc6b	Mm.159621.1	AK016259	-0.52	-0.74	-0.30	0.01119	0.03947	0.14190
1445061_at	Protocadherin 9	Pcdh9	Mm.155985.1	BM941356	-0.59	-0.74	-0.45	0.00315	0.02633	0.10991
1429591_at	transforming, acidic coiled-coil containing protein 1	Tacc1	Mm.150176.1	BE862546	-0.45	-0.75	-0.16	0.03907	0.04171	0.39054
1423325_at	pinin	Pnn	Mm.22347.1	AV135835	-0.44	-0.75	-0.14	0.01510	0.02462	0.16397
1438801_at	dynamin 3	Dnm3	Mm.132054.1	BB709245	-0.55	-0.75	-0.35	0.02035	0.01569	0.35452
1430449_at	RIKEN cDNA C330002I19 gene	C330002I19R	Mm.133458.1	AK021172	-0.54	-0.75	-0.32	0.00521	0.02609	0.01565
1431430_s_at	tripartite motif-containing 59	Trim59	Mm.176695.1	AK012269	-0.42	-0.76	-0.07	0.05972	0.04033	0.35846
1440322_at	---	BB612042	Mm.103559.1	BB612042	-0.36	-0.77	0.04	0.13330	0.04901	0.76562
1437107_at	RAB6B, member RAS oncogene family	Rab6b	Mm.193647.1	AV220161	-0.48	-0.77	-0.19	0.03100	0.03786	0.34050
1428026_at	teashirt zinc finger family member 2	Tshz2	Mm.221068.1	BC028776	-0.39	-0.78	-0.01	0.04365	0.02395	0.89366
1418610_at	solute carrier family 17, member 6	Slc17a6	Mm.70945.1	NM_080853	-0.68	-0.78	-0.59	0.00014	0.01017	0.01269
1437167_at	tropomodulin 2	Tmod2	Mm.74512.1	BB334744	-0.54	-0.78	-0.30	0.00514	0.00653	0.03715
1444513_at	connector enhancer of kinase suppressor of Ras 2	Cnksr2	Mm.101219.1	AI854548	-0.48	-0.80	-0.16	0.03679	0.03314	0.37123
1416959_at	nuclear receptor subfamily 1, group D, member 2	Nr1d2	Mm.26587.1	NM_011584	-0.48	-0.80	-0.17	0.00314	0.00189	0.04131
1457843_at	LY6/PLAUR domain containing 6	Lypd6	Mm.44908.1	AI848492	-0.54	-0.81	-0.26	0.01744	0.01990	0.26815
1459317_at	ankyrin 2, brain	Ank2	Mm.208496.1	BB280924	-0.68	-0.81	-0.54	0.00157	0.03391	0.05471
1442927_at	PTK2 protein tyrosine kinase 2 beta	Ptk2b	Mm.208031.1	BB548690	-0.50	-0.81	-0.20	0.02646	0.04349	0.11410
1456343_at	solute carrier family 35, member F1	Slc35f1	Mm.132397.1	BB540579	-0.53	-0.83	-0.23	0.01341	0.01118	0.05884
1431252_a_at	zinc finger protein 655	Zfp655	Mm.206555.2	AK012336	-0.48	-0.84	-0.11	0.06186	0.04125	0.60215
1429517_at	zinc finger, FYVE domain containing 20	Zfyve20	Mm.26129.1	BC017622	-0.63	-0.87	-0.38	0.00844	0.00014	0.23081
1440031_at	dapper homolog 3	Dact3	Mm.44621.1	BI202427	-0.54	-0.90	-0.18	0.02245	0.00530	0.02274
1438408_at	ankyrin repeat domain 56	Ankrd56	Mm.76746.1	BB131927	-0.58	-0.90	-0.26	0.01586	0.00026	0.26911
1439158_at	tousled-like kinase 1	Tlk1	Mm.132569.1	BB749708	-0.56	-0.91	-0.20	0.03953	0.04894	0.37813
1445473_at	DIRAS family, GTP-binding RAS-like 2	Diras2	Mm.123719.1	BB229114	-0.67	-0.91	-0.43	0.01493	0.04776	0.24360
1443597_at	cofactor required for Sp1 transcriptional activation 2	Crsp2	Mm.187323.1	BB463128	-0.62	-0.94	-0.30	0.01610	0.01351	0.25133
1456827_at	expressed sequence AA987161	AA987161	Mm.138283.1	BB131790	-0.68	-0.95	-0.42	0.00508	0.02914	0.04150
1440318_at	WD repeat domain 35	Wdr35	Mm.131476.1	BB475816	-0.62	-0.96	-0.27	0.02358	0.04541	0.20028
1431129_at	ubiquitin specific peptidase 31	Usp31	Mm.122538.1	BG861783	-0.57	-0.97	-0.18	0.02632	0.00473	0.16523
1447513_at	potassium voltage-gated channel, Shal, member 3	Kcnd3	Mm.54226.1	BE957136	-0.62	-0.98	-0.26	0.02069	0.02902	0.17797
1445631_at	---	BE865226	Mm.156043.1	BE865226	-0.60	-0.99	-0.22	0.02380	0.01433	0.20587
1457142_at	EF hand calcium binding protein 1	Efcbp1	Mm.131089.1	BB667731	-0.61	-0.99	-0.23	0.01915	0.01058	0.11194
1459826_at	Potassium voltage-gated channel, subfamily Q, 2	Kcnq2	Mm.131914.1	BB264132	-0.62	-1.00	-0.24	0.02561	0.03962	0.11719
1439517_at	myb-like, SWIRM and MPN domains 1	Mysm1	Mm.202463.1	BB660695	-0.74	-1.05	-0.43	0.00762	0.03639	0.04338
1455960_at	multiple EGF-like-domains 9	Megf9	Mm.28686.3	BB483642	-0.66	-1.06	-0.26	0.02330	0.03328	0.10127
1459430_at	G protein-coupled receptor 158	Gpr158	Mm.209128.1	BB429778	-0.81	-1.10	-0.51	0.00407	0.00783	0.10519
1431196_at	ATPase, Ca++-sequestering	Atp2c1	Mm.153302.1	BG296252	-0.64	-1.11	-0.18	0.03415	0.01496	0.20644
1447222_at	heat shock protein 12A	Hspa12a	Mm.86619.1	AI854582	-0.68	-1.11	-0.25	0.01935	0.01092	0.05581
1435770_at	thioredoxin domain containing 13	Txndc13	Mm.44400.1	BB215068	-0.68	-1.14	-0.22	0.03786	0.04575	0.26496
1440413_at	RIKEN cDNA A830006F12 gene	A830006F12R	Mm.168328.1	BB800596	-0.57	-1.17	0.03	0.09512	0.00233	0.81713
1430984_at	antizyme inhibitor 1	Azin1	Mm.196297.1	BI155585	-0.72	-1.26	-0.18	0.05041	0.04368	0.41814
1453787_at	thioredoxin domain containing 13	Txndc13	Mm.156901.1	AK015667	-0.78	-1.27	-0.28	0.02433	0.02602	0.07444
1431052_at	Rho GTPase activating protein 12	Arhgap12	Mm.101434.1	BF539472	-0.66	-1.27	-0.04	0.08076	0.03339	0.75645
1444001_at	RIKEN cDNA C230082I21 gene	C230082I21R	Mm.39545.1	AW488249	-0.94	-1.48	-0.40	0.02240	0.04672	0.06925
